# A Lamp2a-linked RNA secreted by ADSCs prevents ENO1–lactylation–glycolysis feedback and cell malignant behavior in triple-negative breast cancer

**DOI:** 10.1038/s41419-026-08517-3

**Published:** 2026-03-02

**Authors:** Shaoqiang Cheng, Bingshu Xia, Liru Li, Shu Zhao, Qihong Zhang, Xue Hui, Xiaolei Liu, WenJing Xiong, Wanzhi Chen, Yue Zhang

**Affiliations:** 1 Breast Cancer Diagnosis and Treatment Center, Harbin Medical University Cancer Hospital, Harbin, China; 2 Department of Thyroid Surgery, The Second Affiliated Hospital of Nanchang University, Nanchang, China

**Keywords:** Breast cancer, Mechanisms of disease

## Abstract

Triple-negative breast cancer (TNBC) is a subtype characterized by the absence of common BC receptors and is closely associated with a hypoxic tumor microenvironment. However, the mechanisms through which TNBCs adapt to hypoxia remain elusive. This study revealed elevated *ENO1* levels in various BC datasets and revealed ENO1 protein lactylation in BC samples through 4D label-free lactylation quantitative proteomics analysis. The results indicated that lactylation increases ENO1 protein stability and enzyme activity, which promotes glycolysis. Notably, as lactate levels increased, a positive feedback loop was established, further promoting lactylation of ENO1. This positive feedback mechanism enables TNBC cells to adapt more efficiently to hypoxia and enhances their malignant behaviors. Lactylation prevented the lysosomal degradation of ENO1. In this study, the characteristics of ENO1, an RNA-binding protein, were assessed to determine how to interfere with its lactylation; specifically, an RNA ligand that can be specifically bound by ENO1 was identified. The RNA ligand was found to be linked to the Lamp2a protein in adipose stem cells (ADSCs) after stable transfection with Lamp2a-TAT and TRA-ligand plasmids. ADSCs seeded in a polyglycolic acid scaffold secreted exosomes containing the Lamp2a-linked ligand. This RNA ligand binds to ENO1 after it enters TNBC cells and further induces the lysosomal degradation of ENO1 by the Lamp2a protein. Consequently, glycolysis, which is associated with malignant cell behaviors, is inhibited. Overall, this study elucidated the role of ENO1 lactylation-mediated glycolysis in TNBC adaptation to hypoxia and provides a strategy for targeting ENO1.

## Introduction

On the basis of the basis of the molecular characteristics of expressed proteins, breast cancer can be categorized into three main types: hormone receptor (HR)-positive breast cancer, HER2-positive breast cancer, and triple-negative breast cancer (TNBC), which does not express any of these three receptors—estrogen receptor (ER), progesterone receptor (PR), or HER2 [1]. Breast cancer is currently treated according to the molecular subtype, with effective therapeutic targets identified for some subtypes. Among traditional breast cancer subtypes, the luminal A (LumA) and luminal B (LumB) types account for approximately 60–70% of cases and are primarily treated with anti-estrogen endocrine therapy. HER2-positive breast cancer accounts for approximately 20% of cases and is treated primarily with anti-HER2 targeted therapy. TNBC is an important clinical subtype, accounting for 15–20% of cases. It is characterized by a high degree of malignancy, a poor prognosis, and a tendency for early relapse and metastasis. Owing to the lack of specific targets, current systemic treatment relies mainly on chemotherapy, making treatment challenging. Therefore, TNBC therapy requires continued exploration of new models and approaches to identify comprehensive and practical molecular targets for adaptable and effective treatments.

The glycolytic pathway, also known as anaerobic glycolysis, is a metabolic process that occurs under low-oxygen conditions, providing substantial energy when needed. Under hypoxic conditions, glucose or glycogen undergoes anaerobic glycolysis, converting pyruvate to lactate and generating ATP. Although less efficient than the tricarboxylic acid (TCA) cycle in ATP production, the glycolytic pathway offers unique advantages by providing diverse building blocks for glycogen, lipid, nucleotide, and protein synthesis [2]. Additionally, it helps fast-dividing cells, including many tumor cells, adapt to hypoxic or nutrient-deprived environments by reducing ROS-induced oxidative stress [3]. Lactate is the final product of glycolysis and can be transported into cells through monocarboxylate transporters (MCTs) on the cell membrane [4]. This transportation can lead to enzymatic or nonenzymatic lysine K-lactylation of proteins. Elevated lactate levels within and outside the cell can promote liver cancer cell proliferation and invasion through lactylation of target proteins [5].

Enolase 1 (ENO1) is a critical glycolytic enzyme responsible for catalyzing the conversion of 2-phosphoglycerate (2-PG) to phosphoenolpyruvate (PEP) during glycolysis in the cytoplasm of eukaryotic cells [6]. Given the high reliance of cancer cells on glycolysis for energy production, ENO1 has emerged as a promising target for cancer therapy [7]. Our current study revealed that TNBC is characterized by significantly elevated ENO1 lactylation levels due to the hypoxic tumor microenvironment. This increase in the ENO1 lactylation level increases ENO1 enzyme activity, promoting glycolysis while suppressing oxidative phosphorylation (OXPHOS) in TNBC cells. This forms a positive feedback regulatory loop, allowing TNBC cells to adapt to hypoxia or nutrient deficiency by promoting tumor cell proliferation and invasion while suppressing apoptosis. Conversely, suppression of ENO1 lactylation mediated by silencing of the lactylation writer EP300 or decreasing intra- and extracellular lactate levels substantially suppresses glycolysis, suppresses TNBC cell malignant behavior, and sensitizes TNBC cells to drugs in hypoxic and/or nutrient-deficient environments. Therefore, targeting ENO1 holds promise as a potential therapeutic strategy for disrupting metabolic processes and suppressing tumor growth in cancer patients.

## Materials and methods

### Proteomics and metabolomics

This study collected TNBC (*n* = 3) and ER-positive BC (*n* = 3) tissues for the Proteomics. The tissues were obtained from patients who underwent breast mass resection at Harbin Medical University Cancer Hospital (Harbin, China). The patients have never received chemotherapy, Endocrine therapy and Small-molecule therapy. The clinical study was approved by the ethics committee of Harbin Medical University Cancer Hospital. Informed consent was obtained from the patients for the publication of pathological tissue images. Proteomics were conducted with the help of Genechem Co., Ltd. (Shanghai, China). The tissue samples are preprocessed at low temperatures, then lysed on ice using a buffer containing 8 M urea, 50 mM Tris-HCl (pH 8.0–8.5), 10 mM NaF, and protease inhibitors. Gentle sonication (avoiding high temperatures) aids lysis, followed by centrifugation to collect soluble proteins. After quantification via BCA assay, proteins undergo reduction at 4 °C with 5–10 mM TCEP to break disulfide bonds and alkylation with 15–20 mM iodoacetamide to block free thiols, preventing reformation of disulfide bonds. A 3 kDa ultrafiltration column is used to exchange buffers with 50 mM ammonium bicarbonate, removing urea and interfering substances. Trypsin (enzyme:protein ratio 1:50) digests proteins at 37 °C for 16–18 h, with digestion terminated by formic acid; peptides are desalted using C18 columns. The enriched peptides were then analyzed by Liquid chromatography-tandem MS (LC-MS/MS), where peptides are separated by high-performance liquid chromatography (HPLC) and fragmented for sequence identification via database searching using MaxQuant. Data are then processed using bioinformatics tools for functional annotation, pathway analysis, and statistical validation.

4D label-free lactylation quantitative proteomics were also conducted by Genechem Co., Ltd. 4D label-free lactylation proteomics integrates advanced mass spectrometry (MS) with pan-specific antibody enrichment to comprehensively profile protein lactylation. Briefly, proteins are extracted from MDA-MB-231 cells with EP300-knockdown or not. Lactylated peptides are enriched using anti-pan-Kla antibodies and then analyzed via 4D proteomics (trapped ion mobility spectrometry [TIMS] coupled with parallel accumulation-serial fragmentation [PASEF] on a timsTOF Pro platform), which enhances peptide identification through four-dimensional separation (retention time, *m*/*z*, ion mobility, and intensity). Data-independent acquisition (DIA) was employed for label-free quantification, followed by MaxQuant database searching to identify lactylation sites and quantify differential abundance.

Metabolomic analysis was conducted with the help of Oebiotech Co., Ltd. (Shanghai, China). Metabolites were extracted from MDA-MB-231 cells with ENO1-knockdown or not, using organic solvents (methanol/acetonitrile/water mixtures), followed by centrifugation and filtration to remove proteins and particulates. LC-MS was adopted with reversed-phase (RP) chromatography for hydrophobic metabolites (e.g., lipids) and hydrophilic interaction chromatography (HILIC) for polar compounds (e.g., sugars, amino acids). Data acquisition modes include full-scan (untargeted metabolomics). Raw data are processed using XCMS software for peak alignment, annotation (via HMDB databases), and statistical analysis.

### Bioinformatics analysis

The transcriptome datasets utilized in our study included GSE65194, GSE103091, and GSE76275. The GSE65194 dataset encompasses transcriptome data for 130 breast cancer samples, comprising 41 TNBC, 30 Her2, 30 LumB, and 29 LumA breast cancer samples, 11 normal breast tissue samples, and 14 TNBC cell lines. The raw CEL files were downloaded from the Gene Expression Omnibus (GEO), and the data were preprocessed via the RMA function within the affy package. Clinical information for the 130 tumor samples included age, height, weight, tumor size, local recurrence, metastasis events, and death events (total survival time). The GSE103091 dataset contains data for 238 TNBC samples sourced from the GEO database. The raw CEL files were processed via the affy package’s RMA function. The clinical data for the 107 tumor samples included age, metastasis (MFS), and death events (total survival time). The GSE76275 dataset comprises multiple subseries, including gene expression data for 198 TNBC tumors and 67 non-TNBC tumors. The raw CEL files were downloaded from the GEO database and preprocessed via the affy package’s RMA function. The clinical data included age, height/weight, tumor–node–metastasis (TNM) grade, tumor differentiation degree, and size/volume. All three breast cancer microarray datasets were obtained via analysis of Affymetrix chips with the probe annotation platform GPL570. For multiple probes for the same gene, we selected the probe with the highest median expression. The tumor sample expression data from GSE65194, GSE103091, and GSE76275 were combined to create a matrix with 633 samples and 20,188 genes (156 non-TNBC samples and 477 TNBC samples). We addressed batch effects introduced by the three datasets using the ComBat function in R’s sva package. Principal component analysis (PCA) was performed both before and after the removal of batch effects. We performed an intersection analysis to identify common significantly upregulated and downregulated genes in the “merged GSE data”, TCGA-BRCA, and METABRIC datasets, resulting in the identification of 598 common upregulated genes and 526 common downregulated genes.

Gene function enrichment analysis of the upregulated (598 genes) and downregulated (526 genes) genes identified from the intersection analysis of the basal vs. other subtypes was conducted using the R package “clusterProfiler”. This analysis focused on Gene Ontology (GO) biological processes (BPs). Survival risk score analysis for the intersection genes was performed with the METABRIC database for Breast Cancer (METABRIC 2016), which includes clinical information for 1,094 tumor tissue samples. Survival risk score analysis for TNBC patients was performed according to the “surv_cox” function in the R package “tinyarray.” This analysis was performed to evaluate prognostic risk in terms of overall survival (OS) according to the expression of the 598 significantly upregulated genes and 526 significantly downregulated genes in breast cancer samples.

### Stable isotope-resolved metabolomics

Glucose ^13^C-metabolic flux analysis (^13^C-MFA) employs isotope tracing with LC-MS to quantify metabolic pathway fluxes in MDA-MB-231 cells with ENO1-knockdown or not. MDA-MB-231 cells were cultured with ^13^C-labeled glucose to allow isotopic incorporation into metabolic intermediates. Metabolites of TCA cycle intermediates were analyzed via LC-MS. High-resolution mass spectrometry distinguishes isotopologue distributions (M0, M + 1…M + 6) with <1% abundance sensitivity. Data processing and ion annotation based on accurate mass were performed in TraceFinder 5.0 (Thermo Fisher) and Xcalibur 4.0 (Thermo Fisher). The correction used both a general applicable correction matrix based on Eqn and an isotopic correction matrix that was generated by treating a control group of unlabeled samples.

### Cell culture and treatments

A panel of breast cancer cell lines including TNBC cell (MDA-MB-231, MDA-MB-468, BT549 and Hs578T), luminal breast cancer cells (MCF-7 and T47-D) and HER2-positive breast cancer cells (SKBR3 and BT474), normal human mammary epithelial cells (MCF-10A), the human renal epithelial cell line 293 T and adipose stem cells (ADSCs) were purchased from the Chinese Academy of Sciences (Shanghai, China). The cells were cultured in Dulbecco’s modified Eagle’s medium (DMEM) or RPMI-1640 medium (Zhong Qiao Xin Zhou Biotechnology Co., Ltd., Shanghai, China). These culture media were supplemented with 10% fetal bovine serum (FBS) (Zhong Qiao Xin Zhou Biotechnology Co., Ltd., Shanghai, China) and penicillin (100 IU/ml)/streptomycin (100 μg/ml) (Zhong Qiao Xin Zhou Biotechnology Co., Ltd., Shanghai, China). The cell cultures were maintained in a humidified incubator at 37 °C under a 5% CO_2_ atmosphere. The hypoxia experiments were conducted using a modular incubator chamber flushed with a certified gas mixture. The conditions have been explicitly stated as: 1% O₂, 5% CO₂, balanced with N₂, at 37 °C. Sodium L-lactate (#L7022) and sodium oxamate (#565-73-1) were obtained from Millipore Sigma (USA). Cells were treated with 20 mM sodium L-lactate or 20 mM sodium oxamate for 48 h, unless otherwise noted for specific assays. These compounds were used to promote and inhibit lactylation in cells, respectively. Cycloheximide (#66-81-9) was also obtained from Millipore Sigma (USA). MG132 (HY-13259), bafilomycin A1 (HY-100558), and C646 (HY-13823) were purchased from MedChemExpress (USA). ENOblock (AP-III-a4, S7443) was purchased from Selleck (Shanghai, China). A concentration of 10 µM ENOblock was identified as effective, which resulted in an approximately 50% reduction in ENO1 enzymatic activity.

The ENO1 knockdown was achieved using lentiviral transduction of short hairpin RNAs (shRNAs) targeting the human ENO1 mRNA sequence. The specific target sequences for the shRNAs (5’-CGTACCGCTTCCTTAGAACTT-3’). For the rescue experiments, we utilized a custom-designed, shRNA-resistant ENO1 cDNA construct. This was generated by introducing silent mutations into the shRNA target sequence within the ENO1 coding sequence, thereby making the transcript resistant to degradation while encoding the wild-type and mutant proteins.

### Measurements of oxygen consumption rate (OCR) and extracellular acidification rate (ECAR)

OCR analysis was performed using a Seahorse XF Analyzer. Cells are seeded in specialized microplates and equilibrated in a CO_2_-free, buffered medium prior to real-time measurement of extracellular oxygen tension via solid-state fluorescent probes. The assay employs sequential injections of mitochondrial modulators—oligomycin (ATP synthase inhibitor), FCCP (uncoupler), and rotenone/antimycin A (complex I/III inhibitors)—to interrogate specific respiratory parameters: basal respiration, ATP-linked respiration, maximal respiratory capacity, proton leak, and non-mitochondrial oxygen consumption. Results are analyzed using Wave software, with respiratory parameters expressed as pmol O_2_/min/μg protein or per 10^4^ cells, enabling quantitative comparison of mitochondrial dysfunction across experimental conditions.

ECAR was measured using the Seahorse XF Analyzer as well. The assay employed sequential injections of metabolic modulators—typically glucose (glycolytic substrate), oligomycin (ATP synthase inhibitor to force glycolytic dependency), and 2-deoxyglucose (2-DG, hexokinase competitive inhibitor)—to quantify glycolytic parameters: basal acidification, glycolytic capacity, and glycolytic reserve. Proton efflux was measured via solid-state pH sensors, and data were analyzed using Wave software.

### Construction of RNA ligands that specifically target ENO1

Studies have revealed that the ENO1 protein binds to two specific RNA regions: “CCCAGRC” (the letter R represents G or A) and “TTTTTTBTTTTTT” (the letter B denotes T, G, or C) [8]. In this study, we synthesized three RNA ligands targeting ENO1. Ligands 1 and 2 were designed by incorporating each of the two specific regions, respectively, into scrambled RNA scaffolds. In contrast, Ligand 3 contained neither of the known regions but instead included a potential binding site predicted by the RNA-protein interaction analysis website catRAPID (http://service.tartaglialab.com/page/catrapid_group_old). Detailed sequences of all ligands are provided in Supplementary Tables 1 and 2. RNA immunoprecipitation was conducted to select the ligand with the strongest affinity for ENO1. This RNA ligand was found to link lysosomal-associated membrane protein 2a (Lamp2a) protein via the interaction between the TAT peptide and HIV-1 transactivation response element (TAR) RNA via a previously reported method [8]. Briefly, the human Lamp2a protein, KFERQ peptide, and TAT peptide sequences were cloned and inserted into the pEGFP-1 plasmid to generate the Lamp2a protein linked with the TAT peptide. The sequences of the ligand and the TAR were also inserted into the plasmid to generate an RNA ligand with the TAR. The lentivirus-encapsulated plasmids were transduced into ADSCs to stably express the Lamp2a-TAT protein and TAR-ligand RNA. RNA immunoprecipitation was conducted to determine whether Lamp2 is linked to ligands in ADSCs.

### Biotinylated RNA pull-down assay

To further validate the binding specificity between the RNA ligand and ENO1, a competitive biotinylated RNA pull-down assay was performed. Briefly, the ligand 2 sequence and a scrambled control RNA sequence were synthesized and labeled with biotin at the 3’ end. MDA-MB-231 and MDA-MB-468 cell lysates were incubated with the biotinylated RNAs (2 µg) for 2 h at 4 °C to allow for RNA-protein binding. To test specificity, a 50-fold molar excess of unlabeled (non-biotinylated) ligand 2 or unlabeled scrambled RNA was added to the reaction mixture as a specific or non-specific competitor, respectively, prior to the addition of the biotinylated RNA. Streptavidin-coated magnetic beads (Pierce™ Streptavidin Magnetic Beads) were then added to capture the biotinylated RNA and any bound proteins. After extensive washing, the bound proteins were eluted and subjected to Western blot analysis. The presence of ENO1 in the pull-down material was detected using a specific anti-ENO1 antibody. The input lysate (10%) was analyzed in parallel to confirm ENO1 levels.

As indicated by Huppertz et al. [9], the mutation of ENO1 at K343 remarkably decreased the RNA-binding ability. To provide definitive genetic evidence that ligand 2 binds specifically to the canonical RNA-binding domain of ENO1, we employed a rescue-based pull-down assay in ENO1-knockdown (ENO1-KD) cells. We generated ENO1-KD MDA-MB-231 and MDA-MB-468 cells using shRNA and reconstituted them by transiently transfecting plasmids encoding either wild-type ENO1 (WT) or the K343R mutant. The biotinylated ligand 2 and a biotinylated scrambled control RNA (2 µg each) were incubated with the lysates for 2 h at 4 °C. Streptavidin magnetic beads were used to capture the RNA-protein complexes. After thorough washing, the bound proteins were eluted and analyzed by Western blot using an anti-ENO1 antibody to detect which ENO1 variants were pulled down by the ligand.

### In vivo orthotopic tumor formation assay

A total of 80 female BALB/c nude mice (aged 4–6 weeks) were included in the studies, with 5 mice in each group. The mice were randomly assigned to each group by researchers who were blinded to the treatment groups. TNBC cells with ENO1 knockdown or ENO1 mutation were engrafted into female BALB/c nude mice via subcutaneous and tail vein injection, and cell proliferation and metastasis were assessed in vivo. To inhibit ENO1 activity in the orthotopic tumor, ENOblock (10 mg/kg) was administered via subcutaneous injection every other day for a duration of almost 4 weeks. ADSCs stably expressing Lamp2a-TAT protein and TAR-ligand RNA were seeded onto sterilized polyglycolic acid (PGA) scaffolds. The PGA scaffolds with adhered ADSCs were implanted subcutaneously in the backs of the nude mice, with the subcutaneous injection of TNBC cells at the same location. Tumor volume was calculated using measurements obtained with digital calipers every other day for approximately 4 weeks following the appearance of the tumors. These nude mice were procured from and maintained at the Laboratory Animal Center of Harbin Medical University Cancer Hospital (China). All the mice were housed in a specific pathogen-free (SPF) environment with a 12-h light/12-h dark cycle, with temperatures maintained between 21–24 °C and humidity levels of 40–60%. All procedures related to the transplantation of tumor cells into mice were conducted in accordance with the guidelines and regulations established by the Institutional Animal Care and Use Committee of Harbin Medical University Cancer Hospital. After the animal studies, all the mice were humanely euthanized through carbon dioxide inhalation. The tumor dimensions were measured every 3 days for six consecutive measurements, and the tumor volume was computed via the formula *V* = *a* × *b*^2^ × 0.52.

Other detailed materials and methods are shown in the Supplementary files.

## Results

### TNBC tumors exhibit significantly elevated glycolytic activity compared to other types of BC tumors, as evidenced by proteomic and functional metabolic analyses

**Fig. 1:**
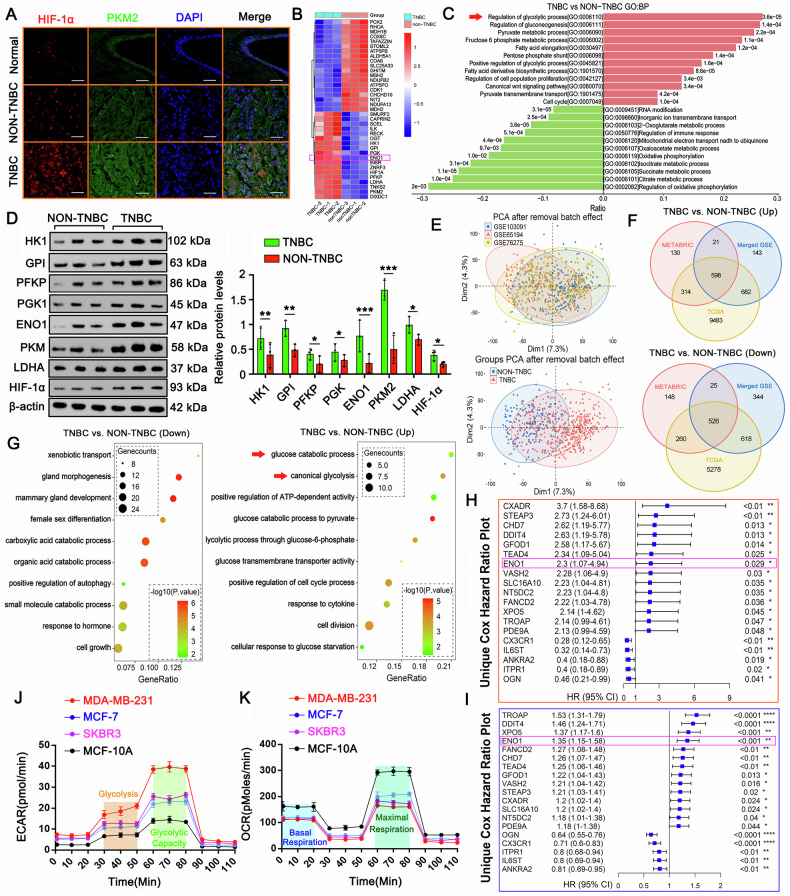
TNBC exhibits enhanced glycolytic activity compared to ER+ BC and normal mammary tissue. **A** Immunofluorescence analysis of HIF-1α and PKM2 expression in normal mammary tissue, ER+ BC (representing non-TNBC tumors), and TNBC tissues. TNBC shows significantly higher expression of both glycolytic regulators compared to ER+ BC and normal tissue. Scale bar: 50 μm. **B** Heatmap of significantly differentially expressed proteins (DEPs) between TNBC and ER+ BC, highlighting metabolic alterations. **C** Gene Ontology (GO) enrichment analysis of upregulated DEPs in TNBC, revealing strong associations with glycolysis, gluconeogenesis, and pyruvate metabolism. **D** Western blot validation of key glycolytic enzymes (HK1, GPI, PFKP, PGK1, ENO1, PKM2, HIF-1α) in TNBC versus ER+ BC. β-actin serves as a loading control. **E** Principal component analysis (PCA) plot of 633 breast tumor samples from integrated GSE datasets after batch-effect correction. **F** Venn diagram illustrating the number of commonly upregulated and downregulated genes in TNBC across multiple datasets (GSE, TCGA-BRCA, METABRIC). **G** GO-BP enrichment analyses of integrated datasets (GSE65194, GSE103091, GSE76275, TCGA-BRCA, METABRIC) in TNBC versus non-TNBC, demonstrating significant enrichment of glycolysis-related gene sets (FDR < 0.05). **H**, **I** the relationship between patient survival time and DEGs using a Cox proportional hazards model and clinical survival and prognosis information provided in the GSE103091 dataset and METABRIC database. **J**, **K** OCR and ECAR were tested in TNBC cell line MDA-MB-231, the Luminal cell line MCF-7, the HER2-positive cell line SKBR3, and the non-tumorigenic human mammary epithelial cell line MCF-10A. Data represent mean ± SEM (*n* = 3 for Proteomics and Western blot assays; *n* = 5 for OCR and ECAR assays); *p < 0.05, **p < 0.01, ***p < 0.001 (unpaired Student’s *t* test for comparisons involving two treatment groups, or a one-way ANOVA for comparisons involving more than two groups).

Glycolysis is a hallmark metabolic feature of most tumors. In this study, we assessed glycolytic activity in normal mammary gland tissue, estrogen receptor-positive breast cancer (ER+ BC, representing non-TNBC tumors), and TNBC tissues by evaluating the expression levels of two well-known glycolysis regulators: hypoxia-inducible factor 1-alpha (HIF-1α) and pyruvate kinase M2 (PKM2). Immunofluorescence analysis revealed significantly higher expression of HIF-1α and PKM2 in ER+ BC compared to normal mammary tissue. Notably, TNBC exhibited even greater upregulation of both markers than ER+ BC (Fig. 1A). To further investigate the molecular differences between TNBC and ER+ BC, we performed quantitative proteomic profiling (Supplementary Table 4). Figure 1B shows a heatmap of the most significantly differentially expressed proteins (DEPs). Gene Ontology (GO) biological process enrichment analysis of upregulated DEPs in TNBC (vs. ER+ BC) identified strong associations with glycolytic processes, gluconeogenesis, and pyruvate metabolism (Fig. 1C). Most of these processes were directly or indirectly linked to glycolysis. Key glycolytic enzymes, including HK1, GPI, PFKP, PGK1, ENO1, PKM2, and HIF-1α, were significantly elevated in TNBC. Western blot analysis confirmed their upregulation in TNBC relative to ER+ BC (Fig. 1D). Strikingly, ENO1 and PKM2 demonstrated the most pronounced overexpression in TNBC tissues. These findings collectively indicate that TNBC tumors exhibit substantially enhanced glycolytic activity compared to ER+ BC, suggesting a critical role of metabolic reprogramming in TNBC aggressiveness.

To further confirm the conclusion, we analyzed the databases, including three GSE datasets (GSE65194 [10–12], GSE103091 [13, 14], and GSE76275 [15, 16]), TCGA-BRCA [17], and METABRIC [18]. The GSE65194 dataset includes information on 41 TNBC and 89 non-TNBC samples, including 30 Her2-positive, 30 LumB, and 29 LumA breast cancer tissue samples. The GSE103091 dataset includes information on a total of 238 TNBC samples, and the GSE76275 is a SuperSeries consisting of information on 198 TNBC tumors in SubSeries GSE76124 and 67 non-TNBC tumors in SubSeries GSE76274. We subsequently constructed an integrated expression matrix comprising data on 633 breast tumor samples after eliminating batch effects (Fig. 1E). To increase the accuracy of classifying the integrated breast cancer samples from different studies, we reclassified these mixed breast tumor samples on the basis of the analysis of microarray 50 (PAM50) gene signature [19]. After reclassification, there were 349 TNBC samples and 284 samples of other types of breast cancer. Subsequent differential gene expression analysis using limma revealed 1398 significantly upregulated genes (*p*. adj <0.05, logFC >0.5) and 1468 downregulated genes (*p*. adj <0.05, logFC < −0.5) between TNBC and non-TNBC samples. Similarly, we retrieved data for 1217 TCGA-BRCA samples and 1904 breast cancer samples from USCS Xena [17] and METABRIC [18, 20], respectively. We then conducted PAM50 classification and differential gene expression analyses between the TNBC and non-TNBC groups using the limma package. A Venn diagram highlighted 598 commonly upregulated and 526 commonly downregulated genes across all the datasets analyzed (Fig. 1F).

Next, we performed (GO-BP) enrichment analyses, which revealed that the genes downregulated in TNBC compared with other types of breast tumors were associated with mammary gland development and morphogenesis, whereas the upregulated genes were linked to glucose metabolism via glycolysis (Fig. 1G). Additionally, we assessed the relationship between patient survival time and DEGs using a Cox proportional hazards model and clinical survival and prognosis information provided in the GSE103091 dataset (Fig. 1H) and METABRIC database (Fig. 1I). Our results consistently revealed 15 upregulated genes associated with a high hazard ratio (HR) and 5 downregulated genes associated with a low HR in TNBC. Notably, ENO1 was among the upregulated genes correlated with a high HR, which is a critical enzyme responsible for converting 2-PG to PEP during glycolysis.

For an in vitro study, we selected four representative cell lines: the triple-negative breast cancer (TNBC) cell line MDA-MB-231, the luminal breast cancer cell line MCF-7, the HER2-positive (HER2+) breast cancer cell line SKBR3, and the non-tumorigenic human mammary epithelial cell line MCF-10A as a normal control. Cellular energy metabolism was analyzed using the Seahorse XF Analyzer to measure oxygen consumption rate (OCR, reflecting mitochondrial respiration) and extracellular acidification rate (ECAR, an indicator of glycolytic flux). MDA-MB-231 (TNBC) exhibited significantly higher ECAR and glycolytic capacity compared to the other cell lines (Fig. 1J), suggesting a strong reliance on glycolysis in TNBC. MDA-MB-231, MCF-7, and SKBR3 all displayed reduced basal and maximal respiration relative to MCF-10A (Fig. 1K). Collectively, these findings demonstrate a pronounced glycolytic shift, particularly in TNBC.

### Integrated analysis reveals ENO1 as a master regulator of metabolic reprogramming in TNBC

**Fig. 2:**
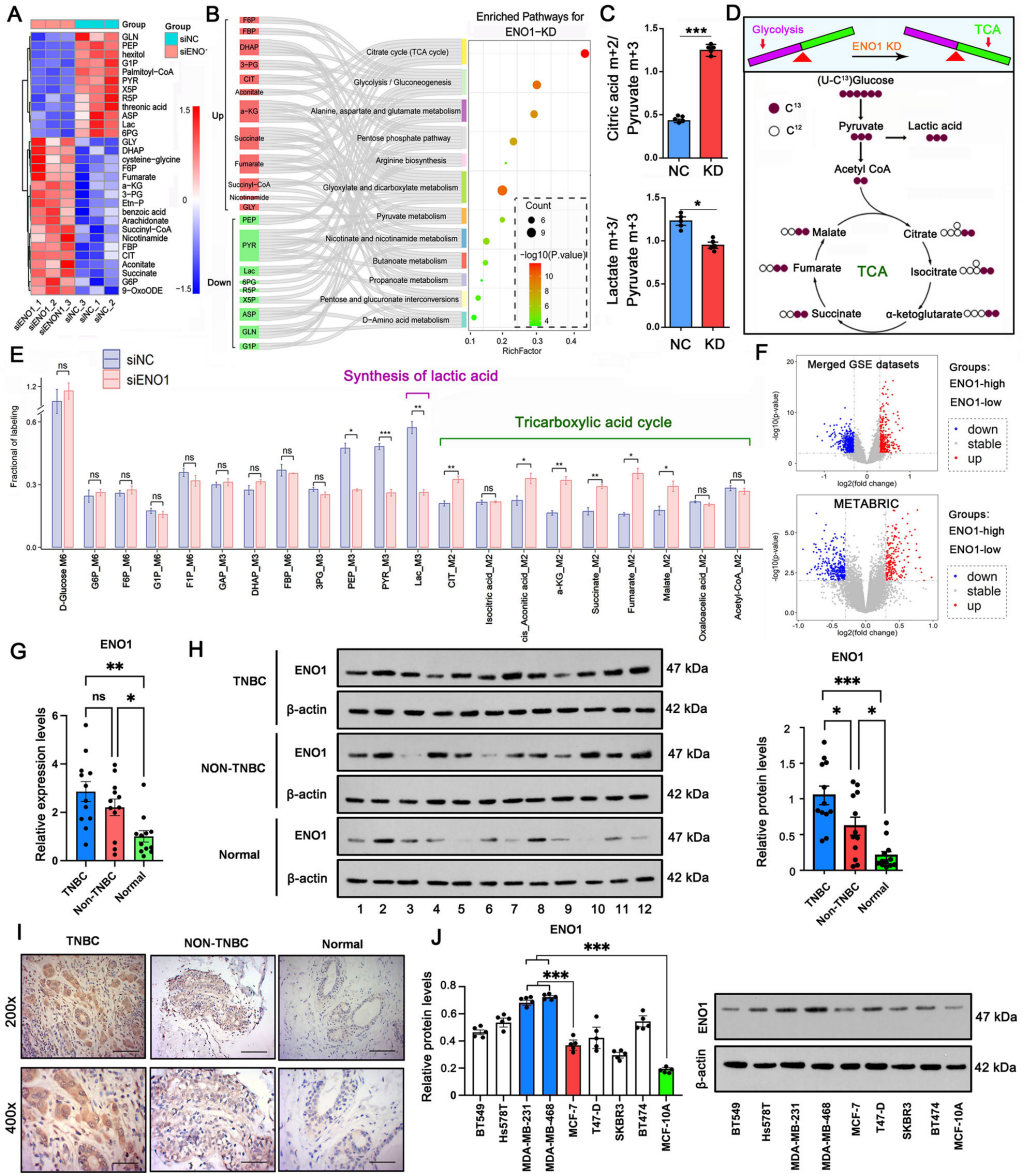
ENO1 is a master regulator of metabolic reprogramming in TNBC. **A** Metabolomic profiling of MDA-MB-231 cells following ENO1 knockdown shows accumulation of upstream glycolytic intermediates (G6P, F6P, FBP, DHAP, 3-PG) and decreased lactate, confirming ENO1’s catalytic role in converting 2-PG to PEP. **B** Pathway analysis reveals ENO1 depletion disrupts glycolysis, TCA cycle, and amino acid metabolism. **C** ^13^C-MFA demonstrates reduced glycolytic flux ( ↓ m + 3 lactate/pyruvate) and enhanced oxidative metabolism ( ↑ m + 2 citrate/pyruvate) upon ENO1 knockdown. **D** Schematic of ENO1’s gatekeeper function in maintaining glycolytic dominance while suppressing mitochondrial respiration. In addition, a Schematic representation of ^13^C-labeled glucose metabolism. **E** Key metabolite changes post-ENO1 knockdown, showing coordinated TCA cycle activation (citrate, α-KG, succinate, fumarate) with glycolytic suppression. **F** Volcano plots of DEGs between ENO1-high and ENO1-low TNBC groups in integrated GSE datasets (Up) and METABRIC cohort (Down), highlighting metabolic-associated genes. **G** Comparison of *ENO1* mRNA levels among TNBC and non-TNBC tissues and normal mammary gland tissues via qPCR. **H** Comparison of ENO1 protein levels among TNBC and non-TNBC tissues and normal mammary gland tissues via Western blot analysis. **I** Comparison of ENO1 protein levels among TNBC and non-TNBC tissues and normal mammary gland tissues by immunohistochemistry. The magnification (×200, Scale bar = 120 μM; ×400, Scale bar = 50 μM). **J** Determination of ENO1 protein levels in various subtypes of breast cancer and normal mammary epithelial cell lines via Western blot analysis. Data represent mean ± SEM (*n* = 3 for Metabolomics assays, *n* = 5 for ^13^C-MFA and other cellular assays, *n* = 12 for clinical studies); *p < 0.05, **p < 0.01, ***p < 0.001 (unpaired Student’s *t* test for comparisons involving two treatment groups, or a one-way ANOVA for comparisons involving more than two groups). Ns nonsignificant difference.

Our study implied ENO1 as a critical driver of glycolytic reprogramming in triple-negative breast cancer. Metabolomic profiling following ENO1 knockdown in MDA-MB-231 cells revealed significant accumulation of upstream glycolytic intermediates, such as Glucose-6-Phosphate(G6P), Fructose-6-Phosphate(F6P), Fructose-1,6-Bisphosphate(FBP), Dihydroxyacetone Phosphate (DHAP), 3-Phosphoglycerate(3-PG), which is in accordance with ENO1’s essential role in catalyzing the 2-PG to PEP conversion (Fig. 2A and Supplementary Table 5). Pathway analysis demonstrated profound dysregulation of core metabolic processes, including glycolysis, TCA cycle, and amino acid metabolism (Fig. 2B). Notably, ENO1 depletion triggered a metabolic shift from glycolysis to oxidative metabolism, characterized by increased TCA cycle intermediates (citrate, α-KG, succinate) and decreased lactate production. Stable isotope-resolved metabolomics (^13^C-MFA) validated this transition, showing reduced glycolytic flux (decreased m + 3 lactate/pyruvate ratio) and enhanced oxidative metabolism (increased m + 2 citrate/pyruvate ratio) (Fig. 2C). Figure 2D shows the schematic of ENO1’s gatekeeper function in maintaining glycolytic dominance while suppressing mitochondrial respiration. In addition, Fig. 2D shows a schematic representation of ^13^C-labeled glucose metabolism. The coordinated elevation of multiple TCA cycle metabolites (citrate, α-KG, succinate, fumarate) was in accompany with lactate reduction after ENO1 knockdown (Fig. 2E). These findings collectively demonstrate that ENO1 serves as a metabolic gatekeeper in TNBC, maintaining glycolytic dominance while suppressing mitochondrial respiration.

Data from three GSE datasets and METABRIC databases were used for the analysis of ENO1 functions in TNBC. We stratified TNBC samples into two groups (the ENO1-high and ENO1-low groups) on the basis of the median ENO1 expression level in each of the breast cancer datasets we analyzed. We then identified DEGs between these two groups via the limma method and subsequent Clusterprofiler method. This analysis revealed 323 genes whose expression was significantly upregulated (*p* adj <0.01, logFC >0.3) and 471 genes whose expression was significantly downregulated (*p* adj <0.01, logFC < −0.3) in our integrated GSE datasets (Fig. 2F). Similarly, in the METABRIC 2016 dataset, we identified 228 significantly upregulated genes (*p* adj <0.01, logFC >0.3) and 295 significantly downregulated genes (*p* adj <0.01, logFC < −0.3) (Fig. 2F). As expected, a subset of upregulated DEGs in the high-ENO1 TNBC group exhibited concordant enrichment in both GO-BP terms and Kyoto Encyclopedia of Genes and Genomes (KEGG) signaling pathways related to glucose metabolism and glycolysis (Supplementary Fig. 1A). This finding suggests increased glycolytic flux in these subgroups of TNBC patients. Importantly, the expression level of ENO1 was strongly negatively associated with both OS and metastasis-free survival (MFS); specifically, patients with high ENO1 mRNA levels had poorer OS and MFS across the different analyzed breast cancer datasets (Supplementary Fig. 1B). This negative correlation became more evident when comparing the high- and low-ENO1 expression groups among TNBC patients.

We next interrogated ENO1 expression through multiple approaches: qPCR (Fig. 2G), Western blot analysis (Fig. 2H), and immunohistochemistry (IHC) (Fig. 2I). In patient-derived tissues, ENO1 expression was significantly upregulated at both the mRNA and protein levels in TNBC and non-TNBC samples relative to normal controls. Strikingly, a comparative analysis between cancer subtypes showed that ENO1 protein, but not its mRNA, was more abundant in TNBC than in non-TNBC. This suggests that ENO1 expression is also controlled at the post-transcriptional level in TNBC. This pattern was corroborated in a panel of breast cancer cell lines representing major subtypes (TNBC, luminal, HER2+) and normal mammary epithelial cells. Consistent with the patient data, the highest ENO1 protein levels were observed in the basal-like TNBC lines MDA-MB-231 and MDA-MB-468 (Fig. 2J), which also exhibited superior ENO1 protein levels to the ER-positive line MCF-7. Consequently, we selected these two cell lines for the remainder of our studies.

### ENO1 promotes the viability, invasion, and colony formation of TNBC through acting as a master regulator of glycolysis

To elucidate the role of ENO1 in TNBC cells, we generated cell lines with ENO1 knockdown or overexpression from the same parental cells (MDA-MB-231 and MDA-MB-468) (Supplementary Fig. 2A). ENO1 silencing largely inhibited the viability (Supplementary Fig. 2B), invasion (Supplementary Fig. 2C), and colony formation (Supplementary Fig. 2D), whereas the overexpression of ENO1 induced the opposite effects in both cell lines. This knockdown and overexpression manipulation resulted in a decrease or an increase in enzymatic activity, respectively, compared with that in their parental control cells (Supplementary Fig. 2E). Considering the pivotal role of ENO1 as a glycolytic enzyme involved in the conversion of 2-PG to PEP, we investigated how changes in its expression impact glycolysis in TNBC cells [21]. As anticipated, alterations in ENO1 expression levels correspondingly influenced the levels of glycolytic metabolites such as pyruvate, PEP, and the end product of glycolysis, lactate. ENO1-knockdown cells have significantly lower levels of pyruvate, PEP, and lactate, whereas ENO1-overexpressing cells exhibited notable increases in the levels of these metabolites (Supplementary Fig. 2F–H). The conversion of glucose to lactate leads to the extrusion of protons into the extracellular medium, a phenomenon quantifiable through the measurement of the extracellular acidification rate (ECAR). Therefore, the ECAR serves as a direct indicator of glycolytic metabolism. The ECAR measurements revealed that ENO1 knockdown in both TNBC cell lines significantly decreased the proton efflux rate attributed to glycolysis (glycoPER) and the glycolytic capacity (Supplementary Fig. 2I). Moreover, both basal glycolysis and glycolytic capacity were notably increased in cells overexpressing ENO1.

To determine whether the glycolytic activity of ENO1 underlies the observed malignant phenotypes in TNBC cells, we treated cells with ENOblock (AP-III-a4), a specific enolase inhibitor [22, 23]. Treatment with 10 µM ENOblock, which reduced enolase activity by approximately 50%, resulted in a suppression of cell viability (Supplementary Fig. 3A, B). Concurrently, this treatment significantly abrogated glycolysis, as evidenced by decreased levels of pyruvate (Supplementary Fig. 3C), PEP (Supplementary Fig. 3D), and lactate (Supplementary Fig. 3E), along with reduced glycolytic proton efflux rate (glycoPER) and glycolytic capacity (Supplementary Fig. 3F). Furthermore, this concentration of ENOblock markedly inhibited cell invasion and colony formation (Supplementary Fig. 3G, H). These pharmacological findings strongly support the conclusion that ENO1-driven glycolysis is crucial for sustaining key aggressive behaviors in TNBC cells.

### Lactate-induced lactylation of ENO1 increases its enzymatic activity and stability

**Fig. 3:**
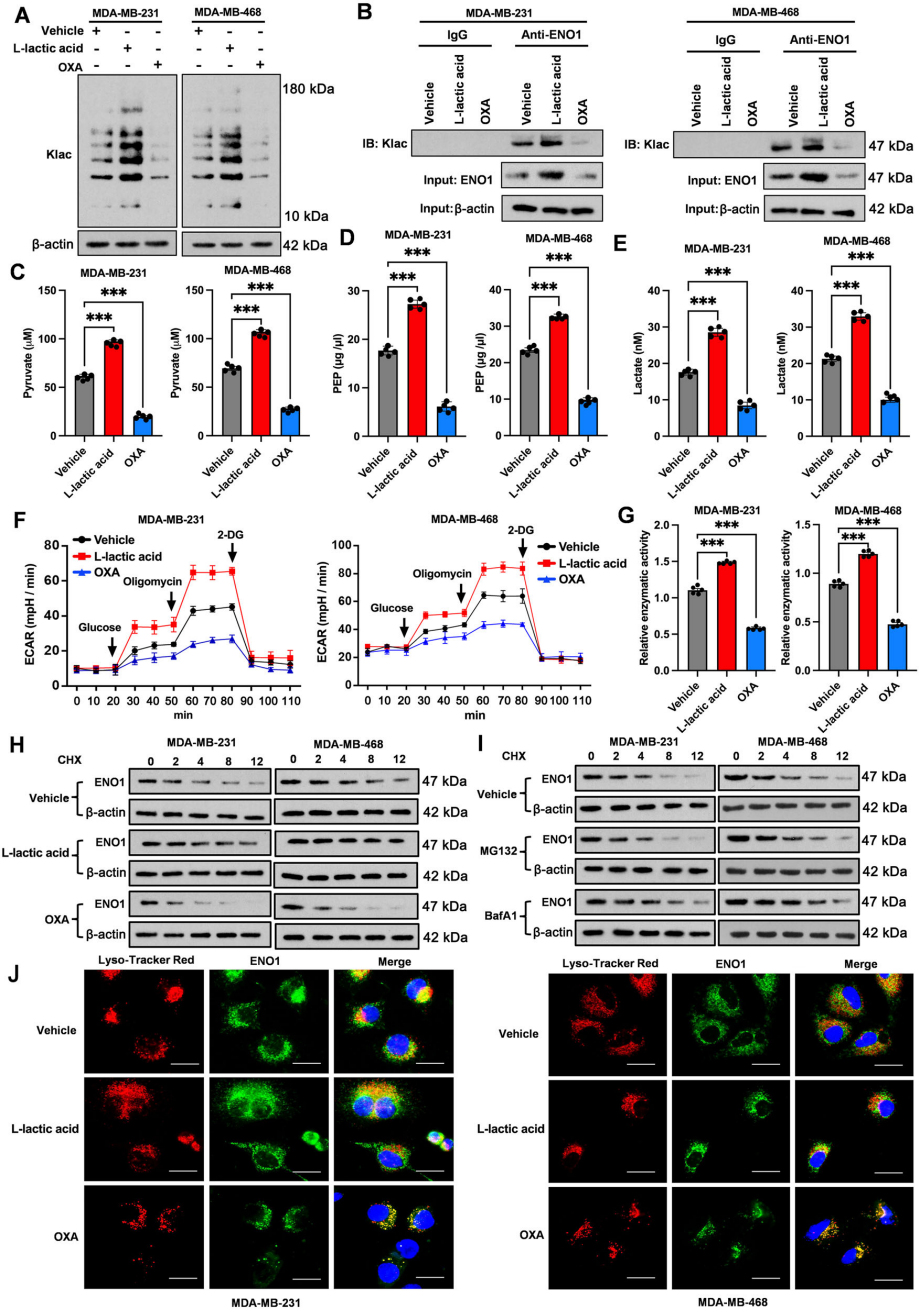
Effect of ENO1 lactylation on the glycolytic metabolism and ENO1 protein stability of TNBC cells. **A** Assessment of pan-lactylation in total protein extracts of vehicle-, L-lactate acid-, and oxamate (OXA)-treated MDA-MB-231 and MDA-MB-468 cell lines via Western blot analysis. **B** Evaluation of ENO1-specific lactylation in vehicle-, L-lactate acid-, and OXA-treated MDA-MB-231 and MDA-MB-468 cell lines by immunoprecipitation. Measurement of the intracellular pyruvate (**C**), PEP (**D**), and lactate (**E**) contents in the vehicle-, L-lactate acid-, and OXA-treated MDA-MB-231 and MDA-MB-468 cell lines. **F** ECAR measurement in vehicle-, L-lactate acid-, and OXA-treated MDA-MB-231 and MDA-MB-468 cell lines. **G** Determination of relative ENO1 enzymatic activity in vehicle-, L-lactate acid-, and OXA-treated MDA-MB-231 and MDA-MB-468 cell lines. **H** Protein stability assay. Cells were treated with cycloheximide (CHX) for the indicated times after pre-treatment with lactate or OXA. ENO1 protein levels were assessed by Western blot. **I** Determination of ENO1 protein stability in MDA-MB-231 and MDA-MB-468 cell lines in the absence or presence of MG132 or BafA1 when exposed to cycloheximide over various time intervals, as revealed by Western blot analysis. **J** Characterization of the intracellular colocalization of ENO1 with lysosomes via immunofluorescence. Scale bar: 5 μm. Data represent mean ± SEM (*n* = 5 for all assays); ***p < 0.001 (a one-way ANOVA for comparisons involving more than two groups).

Emerging evidence demonstrated that lactate, in addition to being a metabolic byproduct, can serve as a precursor for protein lactylation [24]. This finding raised the question of whether lactate accumulation has a feedback effect on promoting ENO1 lactylation, thus influencing its enzymatic activity and role in driving glycolysis. To explore this, we treated two TNBC cell lines with exogenous lactic acid or sodium oxamate, an inhibitor of lactate dehydrogenase known to reduce lactate production [25], and evaluated their impact on protein lactylation. Immunoblotting of whole-cell lysates with a pan-protein lactylation antibody revealed a prominent increase in overall protein lactylation in cells treated with lactic acid compared with that in control cells (Fig. 3A). In contrast, supplementation with sodium oxamate markedly reduced the levels of overall protein lactylation (Fig. 3A). More specifically, exogenous lactic acid substantially promoted the lactylation of the ENO1 protein, as assessed by immunoblotting for lactyl-lysine following immunoprecipitation of ENO1, whereas sodium oxamate suppressed this modification (Fig. 3B). Importantly, these treatments caused substantial changes in the total protein levels of ENO1 as shown in the input controls (Fig. 3B). Lactic acid increasing and sodium oxamate decreasing ENO1 protein levels. Furthermore, TNBC cells treated with exogenous lactic acid presented substantial increases in the levels of intracellular pyruvate (Fig. 3C), PEP (Fig. 3D), and lactate (Fig. 3E) and in the glycoPER and glycolytic capacity (Fig. 3F), indicating a significant increase in glycolysis. Conversely, TNBC cells treated with sodium oxamate presented significant reductions in these glycolytic products and ECAR values. These changes could be attributed to alterations in the enzymatic activity of ENO1, which increased notably upon the addition of exogenous lactic acid but decreased in the presence of sodium oxamate (Fig. 3G). While lactic acid treatment had a minimal effect on cell proliferation (Supplementary Fig. 4A), it promoted the invasion (Supplementary Fig. 4B) and colony formation (Supplementary Fig. 4C) of TNBC cells. In contrast, sodium oxamate inhibited all of these cellular behaviors.

The alterations in ENO1 enzymatic activity caused by lactic acid and sodium oxamate prompted us to investigate whether these treatments influence ENO1 protein stability. To address this question, we treated the manipulated cells with cycloheximide, a eukaryotic protein synthesis inhibitor [26], and then determined the ENO1 protein levels at various time points. Interestingly, lactic acid markedly delayed ENO1 protein degradation compared with that in the control cells, whereas sodium oxamate accelerated ENO1 protein degradation in the presence of cycloheximide (Fig. 3H). Notably, the rate of ENO1 protein degradation was decreased by BafA1 but not MG132 (Fig. 3I), indicating that ENO1 undergoes lysosomal autophagic degradation rather than ubiquitin‒proteasome system-mediated degradation. The immunofluorescence results, which demonstrated that lactic acid suppressed the colocalization of the ENO1 protein with lysosomes, whereas sodium oxamate increased it (Fig. 3J), further corroborated this finding.

### Hypoxia amplifies the ENO1 lactylation-glycolysis feedback loop

**Fig. 4:**
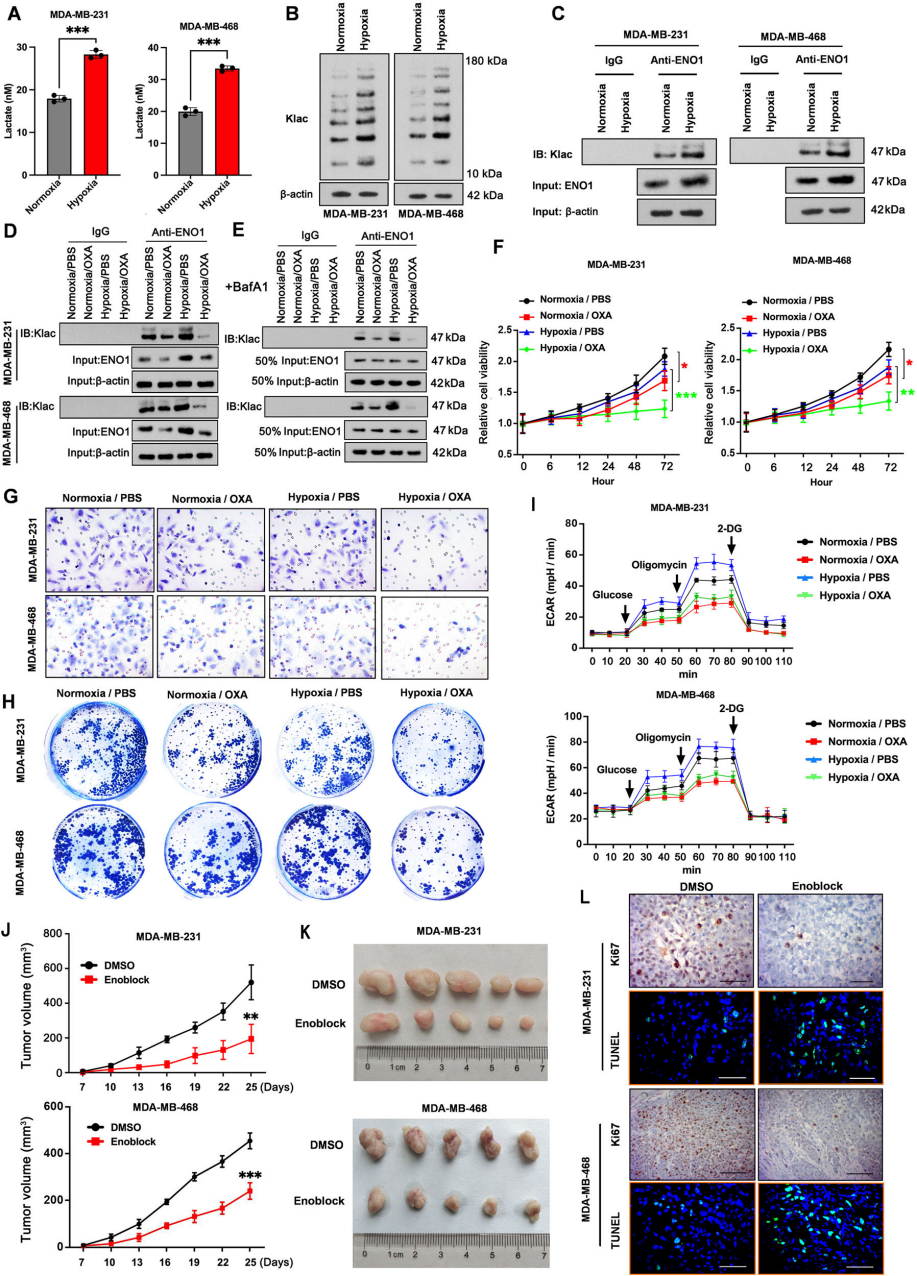
Hypoxia amplifies the ENO1 lactylation-glycolysis feedback loop. **A** Measurement of the intracellular lactate content in the MDA-MB-231 and MDA-MB-468 cell lines under normoxic and hypoxic conditions. **B** Assessment of pan-lactylation in total protein extracts of the MDA-MB-231 and MDA-MB-468 cell lines exposed to normoxic and hypoxic conditions, as determined by Western blot analysis. **C** Evaluation of ENO1-specific lactylation in MDA-MB-231 and MDA-MB-468 cell lines exposed to normoxic and hypoxic conditions, as revealed by immunoprecipitation. **D** Evaluation of ENO1-specific lactylation in MDA-MB-231 and MDA-MB-468 cell lines exposed to normoxic and hypoxic conditions in the absence or presence of OXA, as demonstrated by immunoprecipitation. **E** MDA-MB-231 and MDA-MB-468 cells were pre-treated with BafA1 to inhibit lysosomal degradation before being exposed to normoxia, hypoxia, or hypoxia with OXA. Evaluation of ENO1-specific lactylation in MDA-MB-231 and MDA-MB-468 cell lines was demonstrated by immunoprecipitation. Determination of cell viability (**F**), invasion (**G**), and colony formation ability (**H**) in the MDA-MB-231 and MDA-MB-468 cell lines exposed to normoxic and hypoxic conditions in the absence or presence of OXA. **I** ECAR of MDA-MB-231 and MDA-MB-468 cells exposed to normoxic and hypoxic conditions in the absence or presence of OXA. **J** Tumor growth curves of xenografts from MDA-MB-231 and MDA-MB-468 cells pre-treated with ENOblock or vehicle (DMSO). **K** Representative orthotopic tumors derived from DMSO- or ENOblock-pretreated MDA-MB-231 and MDA-MB-468 cells. **L** Evaluation of cell proliferation and apoptosis via Ki67 and TUNEL immunohistochemistry, respectively, of tumor sections derived from DMSO- or ENOblock-pretreated MDA-MB-231 and MDA-MB-468 cells. Scale bar: 50 μm. Data represent mean ± SEM (*n* = 5 for all assays); *p < 0.05, **p < 0.01, ***p < 0.001 (unpaired Student’s *t* test for comparisons involving two treatment groups, or a one-way ANOVA for comparisons involving more than two groups).

The glycolytic end product pyruvate is converted to lactate under low-oxygen stress, a state known as hypoxia. Indeed, both TNBC cell lines exposed to hypoxic conditions for 48 h presented significantly greater lactate production than did those cultured under normoxic conditions (Fig. 4A). Consequently, we questioned whether our earlier findings related to lactate-mediated lactylation would be more pronounced under hypoxia. As anticipated, immunoblotting with a pan-protein lactylation antibody revealed a marked increase in overall protein lactylation in both TNBC cell lines (Fig. 4B). This included specific lactylation of the ENO1 protein, as evidenced by the more abundant immunoblotting of lactyllysine in ENO1-specific precipitates (Fig. 4C). The immunoprecipitation results further demonstrated that inhibiting lactate production with sodium oxamate reduced ENO1 protein-specific lactylation, a phenomenon that was more pronounced in hypoxia-exposed tumor cells than in cells culture under normoxic conditions (Fig. 4D). To determine the specific effect of hypoxia on ENO1 lactylation independent of protein level changes, we performed an additional experiment based on our data indicate that ENO1 lactylation inhibits its lysosomal degradation, thereby increasing its stability. MDA-MB-231 and MDA-MB-468 cells were pre-treated with BafA1 to inhibit lysosomal degradation before being exposed to normoxia, hypoxia, or hypoxia with OXA. As anticipated, under BafA1 treatment, the total ENO1 protein levels (input) were equalized across the normoxic, hypoxic, and hypoxic+OXA conditions, as degradation was blocked. Crucially, under these conditions of equalized ENO1 input, the lactylation signal specifically precipitated by the ENO1 antibody remained strongly induced by hypoxia and suppressed by OXA (Fig. 4E). Treatment with sodium oxamate resulted in the suppression of viability (Fig. 4F), invasion (Fig. 4G), colony formation (Fig. 4H), as well as glycoPER and glycolytic capacity (Fig. 4I); the inhibitory effects of sodium oxamate on most parameters were more pronounced under hypoxic than those under normoxic conditions. Collectively, these results suggest that sodium oxamate-mediated inhibition of endogenous lactate production impairs the ability of TNBC cells to adapt effectively to hypoxia. As shown earlier in this study (Fig. 3), a positive feedback loop in which lactate reinforces glycolysis by promoting ENO1 lactylation. This, in turn, amplifies cellular lactate production in TNBC cells. ENO1 is an essential component of this feedback loop. Given these findings, we explored the possibility of inhibiting tumorigenesis by targeting ENO1 enzymatic activity in hypoxia-exposed TNBC cells. To investigate this, we added ENOblock to TNBC cells cultured under both normoxic and hypoxic conditions. Strikingly, when TNBC cells were exposed to hypoxia, treatment with ENOblock led to significant inhibition of glycolysis, and this effect was much more pronounced that in cells cultured under normoxic conditions (Supplementary Fig. 5A). This stronger inhibitory effect of ENOblock under hypoxia was also observed in the cell viability (Supplementary Fig. 5B), invasion (Supplementary Fig. 5C), and colony formation (Supplementary Fig. 5D). We further assessed the impact of the inhibition of ENO1 enzymatic activity on the in vivo tumorigenesis of TNBC cells. MDA-MB-231 and MDA-MB-468 cells were engrafted into female BALB/c nude mice via subcutaneous injection and subsequently administered ENOblock or vehicle every other day for almost 4 weeks. Notably, ENOblock strongly inhibited the orthotopic tumor growth of MDA-MB-231 and MDA-MB-468 cells (Fig. 4J). By the end of the treatment period, the orthotopic tumors in the ENOblock-treated mice were substantially smaller than those in the vehicle-treated recipients (Fig. 4K). This reduction in tumor size was attributed to the inhibition of MDA-MB-231 cell proliferation and the promotion of apoptosis by ENOblock. This finding was supported by the immunohistochemistry and TUNEL-staining results, which revealed a remarkable reduction in the proportion of Ki67-positive cells and an increase in the proportion of TUNEL-positive cells in the orthotopic tumors of the ENOblock-treated group (Fig. 4L).

### EP300 mediates lactate-induced lactylation of ENO1

**Fig. 5:**
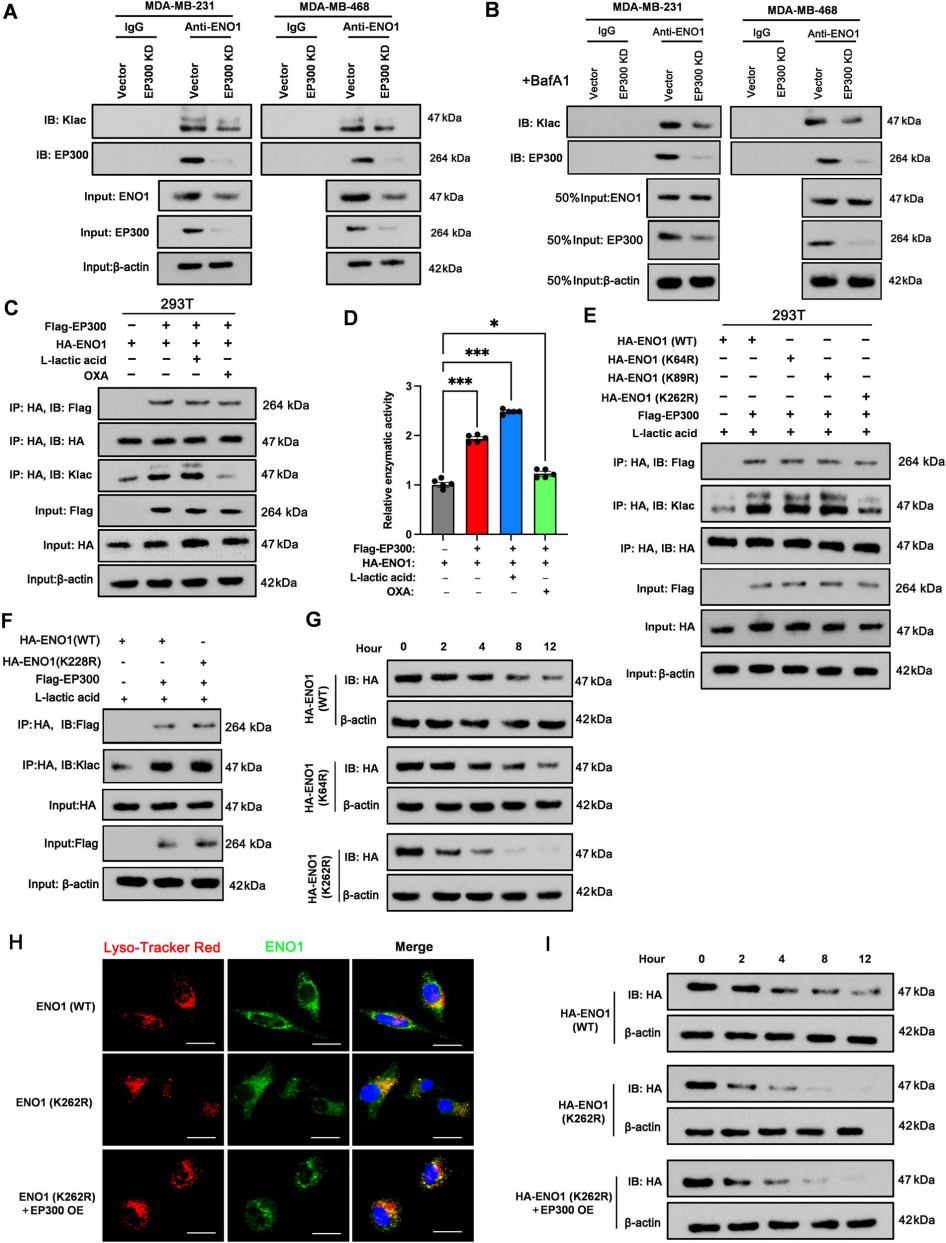
EP300 mediates lactylation of ENO1 at lysine 262. **A** Evaluation of ENO1-specific lactylation in wild-type or EP300-KD MDA-MB-231 and MDA-MB-468 cells, as determined by immunoprecipitation. **B** MDA-MB-231 and MDA-MB-468 cells were pre-treated with BafA1 to inhibit lysosomal degradation. Evaluation of ENO1-specific lactylation in wild-type or EP300-KD MDA-MB-231 and MDA-MB-468 cells, as determined by immunoprecipitation. **C** Immunoprecipitation analysis of ENO1 lactylation in 293T cells expressing HA-ENO1 with or without Flag-EP300, under lactate or OXA treatment. **D** ENO1 enzymatic activity in 293T cells from the experiment in (**C**). **E** Assessment of HA-tagged wild-type or K64-, K89-, and K262-mutated ENO1 lactylation in 293T cells with or without cotransfection of FLAG-tagged EP300 in the presence of L-lactate acid, as revealed by coimmunoprecipitation. **F** Immunoprecipitation analysis of lactylation on wild-type (WT) and K228-mutant HA-ENO1 proteins co-expressed with Flag-EP300. **G** Assessment of ENO1 protein stability in wild-type, K64-mutant and K262-mutant 293T cells exposed to CHX over various time intervals, as determined by Western blot analysis. **H** Characterization of the intracellular colocalization of ENO1 with lysosomes in wild-type ENO1-OE, K262-mutated ENO1-OE, and K262-mutated ENO1- and EP300-co-OE 293T cells via immunofluorescence. Scale bar: 5 μm. **I** Protein stability assay (CHX chase) for HA-ENO1-WT and HA-ENO1-K262R with or without EP300 co-expression. Data represent mean ± SEM (*n* = 5 for all assays); ***p < 0.001 (a one-way ANOVA for comparisons involving more than two groups).

Given the essential role of ENO1 lactylation in the adaptation of TNBC cells to hypoxia, we aimed to elucidate the molecular mechanism underlying lactate-induced lactylation of the ENO1 protein. A study demonstrated that EP300, a well-established histone acetyltransferase, can regulate ENO1 enzymatic activity [27]. Notably, our 4D label-free lactylation quantitative proteomics analysis revealed a significant decrease in lactylation at lysines 64, 89, and 262 of the ENO1 protein in EP300-knockdown MDA-MB-231 cells compared with control MDA-MB-231 cells (Table 1 and Supplementary Fig. 6). We therefore reasoned that EP300 could be a key writer enzyme for ENO1 lactylation. Consistent with this prediction, immunoprecipitation assays showed that EP300 knockdown resulted in a marked decrease in both ENO1 lactylation and total ENO1 protein levels compared to the control (Fig. 5A). To unequivocally determine whether the reduced lactylation is specifically due to the loss of EP300-mediated lactylation rather than merely a consequence of lower total ENO1 protein, we performed a critical experiment. Given our finding that lactylation stabilizes ENO1 by preventing its lysosomal degradation, we pharmacologically inhibited lysosomal function with BafA1. This intervention equalized total ENO1 levels (Input) between control and EP300-knockdown groups. Under these conditions, the lactylation signal specifically immunoprecipitated with ENO1 remained significantly lower in the EP300-knockdown cells than in the controls, confirming a direct role for EP300 in ENO1 lactylation (Fig. 5B). To definitively establish EP300’s function in ENO1 lactylation, we employed a reconstitution system in 293 T cells expressing exogenous Flag-EP300 and HA-ENO1 (Fig. 5C). This approach revealed that EP300 elevates HA-ENO1 levels, an effect potentiated by lactate and inhibited by sodium oxamate. Co-IP assays demonstrated a stable EP300-ENO1 interaction, insensitive to lactate or oxamate. Most importantly, after equalizing the amount of immunoprecipitated HA-ENO1, we detected a substantial increase in lactylation specifically on HA-ENO1 co-expressed with EP300. This lactylation signal was positively regulated by lactate and negatively by sodium oxamate, directly recapitulating our endogenous observations and confirming EP300’s central role. Moreover, cotransfection of HA-ENO1 with Flag-EP300 significantly increased ENO1 enzymatic activity in 293 T cells, and this effect was further amplified by lactic acid supplementation but attenuated by exogenous sodium oxamate treatment (Fig. 5D).

**Table 1:** Differential sites of ENO1 lysine K-lactylation between the EP300 Knockdown group and the control group.

P06733 position	Ctrl/KD ratio	Regulated type	Protein description	Gene name	Score	Charge	Modified sequence
P06733-K89	1.6008	Up	Alpha-enolase	ENO1	117.21	2	LNVTEQEK(1)IKD
P06733-K64	1.8494	Up	Alpha-enolase	ENO1	160.98	2	GVSK(1)AVEHINK
P06733-K262	2.1817	Up	Alpha-enolase	ENO1	179.43	2	YDLDFK(1)SPDDPSR

4D label-free lactylation quantitative proteomics analysis revealed a significant change in lactylation at lysines 64, 89, and 262 of the ENO1 protein in EP300-knockdown MDA-MB-231 cells compared with control MDA-MB-231 cells.

To further assess whether EP300 is essential for ENO1 lactylation, we treated both TNBC cell lines with varying concentrations of C646, a competitive and selective EP300 inhibitor [28], for 48 h. Immunoblotting with a lactylation-lysine antibody in ENO1-specific precipitates in the cell lysates revealed a dose-dependent reduction in ENO1 lactylation levels (Supplementary Fig. 7A). On the basis of these results, we selected 20 µM as the minimum concentration of C646 that exerts the maximum inhibitory effect on ENO1 lactylation for subsequent studies investigating the role of EP300 in TNBC cell glycolysis. Our results demonstrated that inhibiting EP300 with C646 significantly reduced pyruvate (Supplementary Fig. 7B), lactate (Supplementary Fig. 7C), and PEP (Supplementary Fig. 7D) levels, as well as the glycoPER and glycolytic capacity (Supplementary Fig. 7E), in both TNBC cell lines. These attenuated glycolytic phenotypes were associated with significant suppression of ENO1 enzymatic activity by C646 (Supplementary Fig. 7F). This suppression may result from enhanced lysosomal autophagic degradation, as indicated by the substantial increase in colocalization of the ENO1 protein with lysosomes (Supplementary Fig. 7G). Since we demonstrated that ENO1 protein stability is influenced by exogenous lactic acid and sodium oxamate and is linked to ENO1 enzymatic activity in TNBC cells, we were curious whether EP300 inhibition also impacts ENO1 protein stability. To address this question, we added cycloheximide to C646-treated TNBC cells and monitored ENO1 protein levels at various time points. The Western blotting results revealed that the inhibition of EP300 by C646 substantially accelerated the degradation of the ENO1 protein compared with that in control cells (Supplementary Fig. 7H). Importantly, either genetic knockdown or chemical inhibition of EP300 suppressed TNBC cell viability (Supplementary Fig. 7I), invasion (Supplementary Fig. 7J), and colony formation (Supplementary Fig. 7K). These inhibitory effects on tumorigenesis due to the loss of EP300 function were more pronounced under hypoxic conditions, under which glycolysis is the main cellular metabolism process (Supplementary Fig. 7I–K).

### The lysine 262 residue in ENO1 is indispensable for lactylation modification

Next, we delved deeper into the role of lysine residues (K64, K89, and K262) in determining ENO1 protein stability, which was markedly reduced upon EP300 knockdown. To investigate this, we cotransfected 293T cells with Flag-EP300 and either wild-type HA-ENO1 or point mutants, where each lysine was substituted with arginine (K-to-R) (Fig. 5E). In the presence of exogenous lactic acid, Flag-EP300 increased the protein levels of wild-type HA-ENO1 and its K64R/K89R mutants, but had a minimal effect on the K262R mutant. Importantly, after normalizing the amount of immunoprecipitated HA-ENO1, the lactylation levels showed a parallel pattern: it was enhanced for the wild-type and K64R/K89R mutants by EP300, but remained largely unaffected for the K262R mutant. This finding suggests that lactylation at K262, but not at K64 or K89, is the critical modification responsible for the EP300-dependent stabilization of ENO1. Du et al. identified SIRT1/SIRT3 as primary erasers of ENO1 K228 lactylation in HepG2 hepatocellular carcinoma cells [29]. But, it is unclear whether EP300 influences ENO1 K228 lactylation. We hence conducted additional experiments to specifically address this point. Results from co-immunoprecipitation assays demonstrated that EP300 overexpression enhances lactylation in both wild-type ENO1 and the K228R mutant. K228 mutation does not abolish EP300-mediated lactylation, indicating that K228 is not essential for EP300-induced lactylation in our system (Fig. 5F). In the transfected 293T cells, Western blotting analysis revealed that the degradation rate of HA-ENO1 was unaffected by the K64 mutation but accelerated upon the conversion of K262 to R (Fig. 5G). Furthermore, the K262 mutation promoted the lysosomal degradation of ENO1, as evidenced by its enhanced colocalization with lysosomes (Fig. 5H). Consequently, EP300 overexpression failed to rescue the K262-mutant ENO1 from degradation, a finding consistent across immunofluorescence (Fig. 5H) and Western blot analyses (Fig. 5I).

We also co-transfected TNBC cells with Flag-EP300 and either wild-type HA-ENO1 or its K262 mutant. ENO1 knockdown suppressed the viability, invasion, and colony formation of MDA-MB-231 cells under hypoxia (Supplementary Fig. 8A–C). The suppressive effects of ENO1 knockdown were rescued by transfection with wild-type HA-ENO1. In contrast, the K262 mutant failed to restore these malignant phenotypes, despite the co-expression of Flag-EP300. The knockdown of ENO1 significantly reduced glycolytic parameters, including pyruvate and lactate levels, ECAR metrics (glycoPER and glycolytic capacity), and cellular ENO1 enzymatic activity (Supplementary Fig. 8D–G). Reintroducing wild-type ENO1 fully restored all these parameters. In contrast, the K262 mutant was ineffective at reversing the metabolic defects caused by ENO1 knockdown despite the co-overexpression of EP300.

**Fig. 6:**
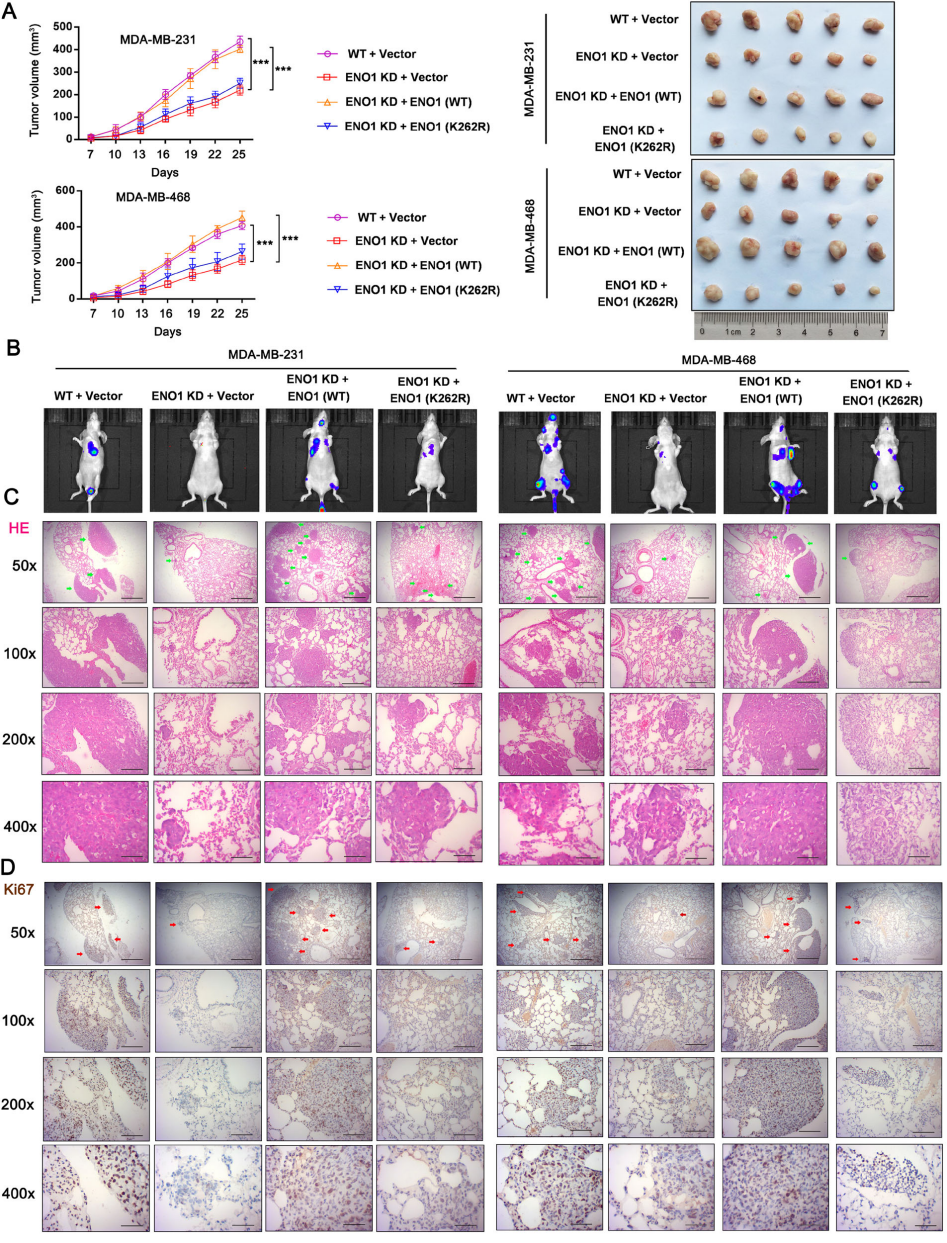
ENO1 K262 lactylation is critical for the growth and lung metastasis TNBC. **A** Subcutaneous tumor formation experiments in MDA-MB-231 and MDA-MB-468 cells with the knockdown of ENO1 and reconstitution with wild type ENO1 and the K262R mutant ENO1. Tumor growth curves of xenografts from MDA-MB-231 and MDA-MB-468 cells (left) and representative orthotopic tumors (right). **B** This study also conducted an additional in vivo study by tail vein injection of MDA-MB-231 and MDA-MB-468 cells. The in vivo imaging assay demonstrated the metastasis of TNBC. **C** H&E staining of lung tissues shows the number and size of metastatic nodules (green arrow). The magnification (×50, Scale bar = 500 μM; ×100, Scale bar = 250 μM, ×200, Scale bar = 120 μM; ×400, Scale bar = 50 μM). **D** Ki67 staining of lung tissues shows the number and size of metastatic nodules (red arrow). The magnification (×50, Scale bar = 500 μM; ×100, Scale bar = 250 μM, ×200, Scale bar = 120 μM; ×400, Scale bar = 50 μM). Data represent mean ± SEM (*n* = 5 for all assays); ***p < 0.001 (a one-way ANOVA for comparisons involving more than two groups).

The functional impairment of the K262 ENO1 mutant was further validated by in vivo assays. Subcutaneous tumor formation experiments in MDA-MB-231 and MDA-MB-468 cells revealed that knockdown of ENO1 significantly suppressed subcutaneous tumor growth in both MDA-MB-231 and MDA-MB-468 cells compared to the control (WT + Vector) group (Fig. 6A). Reconstitution with wild-type ENO1 (ENO1 KD + ENO1 WT group) effectively rescued tumor growth, with tumor volumes comparable to the control group. Crucially, reconstitution with the K262R mutant (ENO1 KD + ENO1 K262R group) failed to restore tumor growth. The tumor volumes in this group remained significantly smaller and were comparable to those in the ENO1 KD group. This study also conducted an additional in vivo study by tail vein injection of MDA-MB-231 and MDA-MB-468 cells. The in vivo imaging assay demonstrated that ENO1 knockdown suppressed metastasis of MDA-MB-231 and MDA-MB-468 cells specially to lung tissues (Fig. 6B). Restoration of wild-type but not K262-mutated ENO1 in ENO1-silenced cells resulted in lung metastasis effects comparable to those for wild-type MDA-MB-231 and MDA-MB-468 cells. Consistent with this, H&E staining of lung tissues confirmed that the number and size of metastatic nodules, which were reduced by ENO1 knockdown, were restored only by the wild-type protein (Fig. 6C). Finally, Ki67 staining indicated that the proliferative capacity of the cancer cells in the lung was compromised by ENO1 knockdown and rescued specifically by the wild-type ENO1, not the K262 mutant (Fig. 6D). These data provide direct evidence that lactylation at the K262 residue is indispensable for ENO1’s oncogenic function in promoting not only local tumor proliferation but also metastasis. This strengthens our conclusion that the lactylation-mediated stabilization and enhancement of ENO1 activity is a key mechanism driving TNBC progression.

### Establishment of a Lamp2a-linked RNA ligand to target the ENO1 protein in TNBC cells

ENO1 not only is a glycolytic enzyme but also functions as an RNA-binding protein. Studies have revealed the features of RNAs that bind to the ENO1 protein [9]. Exploiting these features, we designed three RNA ligands to bind ENO1 proteins. The three ligands were transfected into MDA-MB-231 and MDA-MB-468 cells. An RNA immunoprecipitation (RIP) assay with an anti-ENO1 antibody revealed that all three ligands can bind to the ENO1 protein, with ligand 2 having the highest affinity (Supplementary Fig. 9A). The binding of ligand 2 to the ENO1 protein was further corroborated by the immunofluorescence results, which revealed the colocalization of ligand 2 (red fluorescence) with the ENO1 protein (green fluorescence) (Supplementary Fig. 9B). To unequivocally demonstrate the direct and specific interaction between ligand 2 and ENO1, we employed a competitive biotinylated RNA pull-down assay. As shown in Supplementary Fig. 10A, the biotinylated ligand 2, but not the biotinylated scrambled control RNA, successfully pulled down ENO1 from TNBC cell lysates, confirming the direct binding observed in the RIP assays. Crucially, the inclusion of a 50-fold excess of unlabeled ligand 2 effectively competed for ENO1 binding, leading to a dramatic reduction in the amount of ENO1 co-precipitated with the biotinylated ligand 2. In contrast, the same excess of unlabeled scrambled RNA failed to compete for the interaction, as the signal for ENO1 remained strong. As indicated by Huppertz et al. [9], the mutation of ENO1 at K343 remarkably decreased the RNA-binding ability. We generated ENO1-KD MDA-MB-231 and MDA-MB-468 cells using shRNA and reconstituted them by transiently transfecting plasmids encoding either wild-type ENO1 (WT) or the K343R mutant. As anticipated, the biotinylated ligand 2, but not the scrambled RNA, efficiently pulled down ENO1 from the lysates of control cells and cells reconstituted with ENO1-WT (Supplementary Fig. 10B). The signal was absent in the ENO1-KD group, confirming the specificity of the pull-down for ENO1. Most importantly, the binding of ligand 2 to the ENO1-K343R mutant was severely diminished compared to its binding to the ENO1-WT. The results demonstrated that while ligand 2 efficiently bound to wild-type ENO1, its binding to the K343R mutant was severely diminished. Based on these results, ligand 2 was used for further study.

**Fig. 7:**
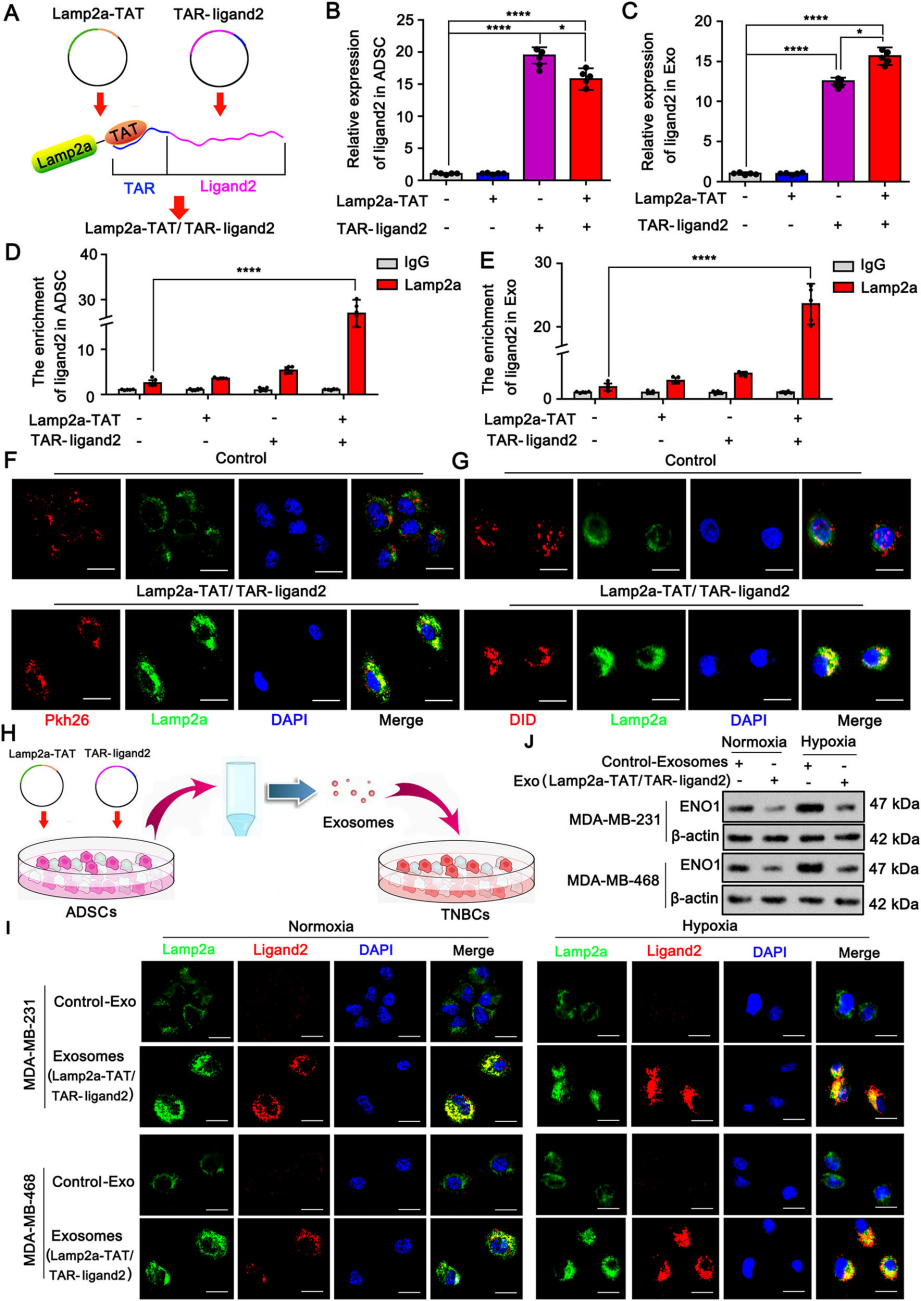
Generation of a Lamp2a-linked RNA ligand to target the ENO1 protein in TNBC cells. **A** Schematic diagram of the generation of the protein‒RNA complex Lamp2a-TAT/TRA-ligand 2. Two plasmids, Lamp2a-TAT and TRA-ligand 2, were established and transfected into ADSCs. Through binding between TAT and TRA, the produced Lamp2a-TAT protein can bind to TRA-ligand 2 RNA to generate a protein‒RNA complex, Lamp2a-TAT/TRA-ligand 2. A PCR assay was conducted to detect ligand 2 RNA in ADSCs (**B**) and secreted exosomes (**C**) after transfection with TRA-ligand 2 and Lamp2a-TAT, alone or in combination. ***p < 0.001 by one-way ANOVA. Using Lamp2a as the precipitate protein, a RIP assay was conducted to detect the enrichment of ligand 2 on the Lamp2a protein in ADSCs (**D**) and exosomes (**E**). Pkh26 (**F**) and DID (**G**) fluorescent dyes were used to assess exosome production in cells. Immunofluorescence analysis revealed that compared with expression of endogenous Lamp2a, cotransfection with Lamp2a-TAT and TRA-ligand 2 promoted Lamp2a in exosomes in control cells. **H** Schematic diagram of how ADSC-secreted exosomes were collected and added to TNBC cells. **I** FISH and immunofluorescence assays were used to detect ligand 2 and Lamp2a in TNBC cells treated with ADSC-secreted exosomes. Endogenous Lamp2a was knocked down in TNBC cells before they were treated with exosomes. **J** Western blot analysis revealed that, compared with control exosomes, exosomes containing Lamp2a-TAT/TRA-ligand 2 decreased ENO1 protein levels in TNBC cells under normoxia and hypoxia. Data represent mean ± SEM (*n* = 5 for all assays); ****p < 0.0001 (a one-way ANOVA for comparisons involving more than two groups).

This study revealed that ENO1 primarily undergoes lysosomal autophagic degradation rather than ubiquitin‒proteasome system-mediated degradation. However, lactylation prevents the degradation of ENO1, probably because the modification hinders the binding of ENO1 to proteins responsible for lysosomal autophagic degradation. To overcome this difficulty of degrading lactylated ENO1, we considered inducing the binding of ENO1 to Lamp2a, a key protein that mediates lysosomal autophagic degradation [30], through ligand 2. Since ligand 2 is an RNA, the binding of ENO1 to ligand 2 is not strongly influenced by lactylation. This assumption was corroborated in the following study. The interaction between Lamp2a and ligand 2 was assessed via a classical method published in a previous study [8]. As shown in Fig. 7A, two plasmids, Lamp2a-TAT and TRA-ligand 2, were constructed. Through binding between TAT and TRA, the produced Lamp2a-TAT protein can bind to TRA-ligand 2 RNA to generate a protein‒RNA complex, Lamp2a-TAT/TRA-ligand 2. In this study, we first generated Lamp2a-TAT/TRA-ligand 2 in ADSCs, rather than in TNBC cells, via transfection of these two plasmids. ADSCs hold great promise for cancer therapy because of their multiple characteristics, such as their continuous proliferation and ability to release extracellular vesicles [31]. Notably, ADSCs can be used to easily generate the indicated protein and RNA, which can be further preloaded in extracellular vesicles for various applications [31]. Through stable expression of Lamp2a-TAT and TRA-ligand 2, ADSCs continuously generated the Lamp2a-TAT/TRA-ligand 2 complex. Lamp2a is a positive marker of multivesicular bodies (MVBs), which can further develop into exosomes. Hence, the exosomes secreted by these ADSCs, which are taken up by the surrounding TNBC cells, carry the Lamp2a-TAT/TRA-ligand 2 complex. As indicated by the results of the PCR assay, the level of ligand 2 RNA was increased in ADSCs after transfection with TRA-ligand 2 alone or in combination with Lamp2a-TAT (Fig. 7B). Ligand 2 RNA was also increased in the exosomes secreted by the cells transfected with TRA-ligand 2 alone or in combination with Lamp2a-TAT (Fig. 7C). Ligand 2 levels in the exosomes were greater after transfection with both Lamp2a-TAT and TRA-ligand 2 than after transfection with TRA-ligand 2 alone, suggesting that Lamp2a is helpful, but not indispensable, for the entry of TRA-ligand 2 into exosomes. Using Lamp2a as the precipitate protein, a RIP assay confirmed the link between Lamp2a and ligand 2 in ADSCs after transfection with both Lamp2a-TAT and TRA-ligand 2 (Fig. 7D). Notably, ligand 2 was barely detected by the RIP assay in control cells or cells individually transfected with Lamp2a-TAT or TRA-ligand 2. These results indicate that the endogenous Lamp2a protein fails to bind with ligand 2 RNA. RIP assays also confirmed the interaction between Lamp2a-TAT and TRA-ligand 2 in exosomes (Fig. 7E). Pkh26 and DID fluorescent dyes were used to assess exosome production in cells. Compared to cells with endogenous Lamp2a expression, those with cotransfection of Lamp2a-TAT and TRA-ligand 2 exhibited increased Lamp2a levels in exosomes (Fig. 7F, G).

Next, ADSC-secreted exosomes were collected and added to TNBC cells for 48 h (Fig. 7H). Fluorescence in situ hybridization (FISH) and immunofluorescence assays were used to detect ligand 2 and Lamp2a in TNBC cells that were treated with ADSC-secreted exosomes. Endogenous Lamp2a was knocked down in TNBC cells before they were treated with exosomes. Lamp2a, but not ligand 2, was detected in TNBC cells treated with exosomes from control ADSCs (Fig. 7I). Much stronger Lamp2a fluorescence with ligand 2 fluorescence was detected after treatment with exosomes from ADSCs cotransfected with Lamp2a-TAT and TRA-ligand 2. This phenomenon was observed in TNBC cells under normoxic and hypoxic conditions. These results indicate that TNBC cells uptake the Lamp2a-TAT/TRA-ligand 2 complex secreted from ADSCs via exosomes. Compared with control exosomes, exosomes containing Lamp2a-TAT/TRA-ligand 2 decreased ENO1 protein levels in TNBC cells under normoxia and hypoxia (Fig. 7J).

### Lamp2a-linked RNA ligands induce ENO1 degradation in lysosomes, thereby suppressing the malignant behavior of TNBC cells

**Fig. 8:**
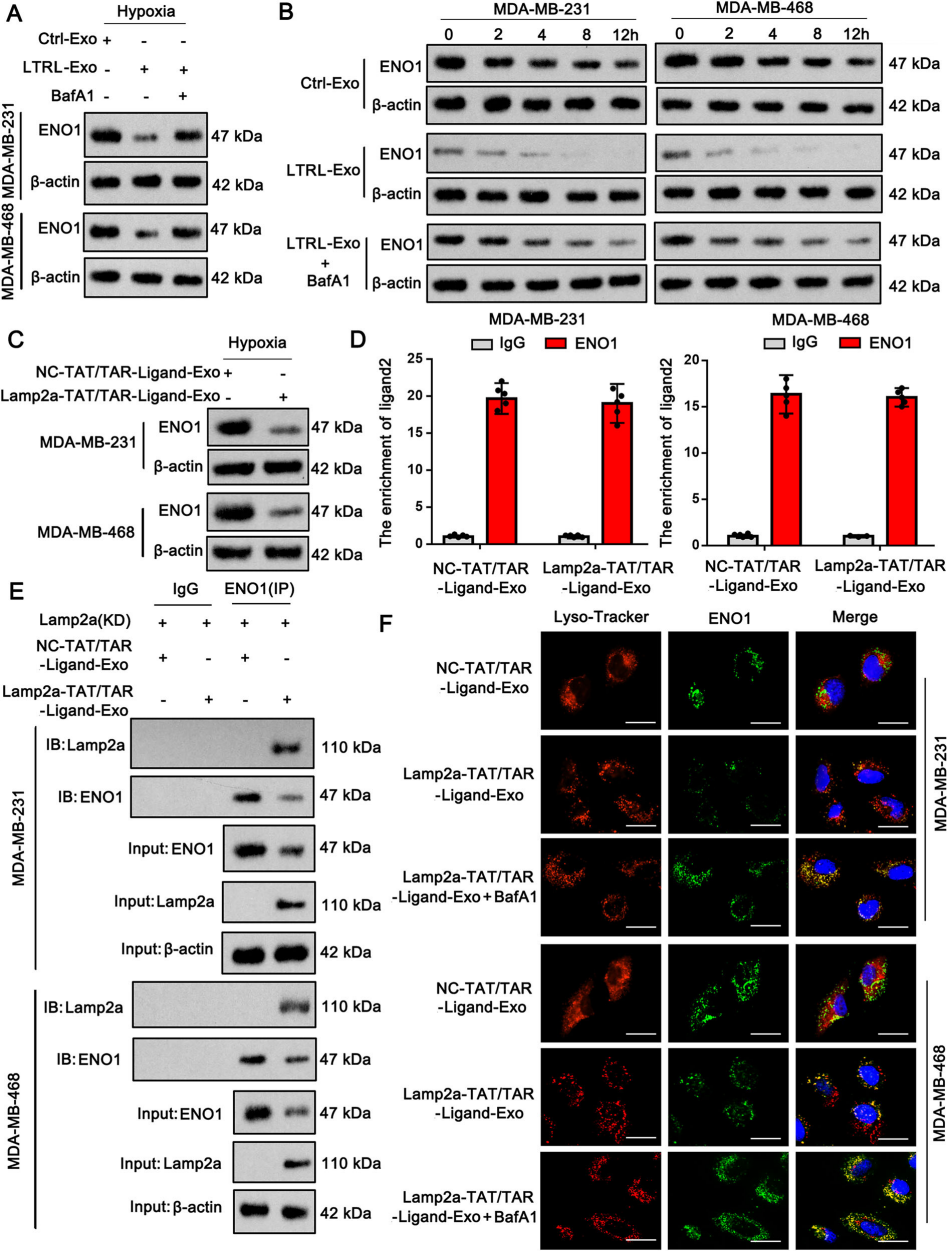
The Lamp2a-linked RNA ligand induces ENO1 degradation in lysosomes. **A** Western blot analysis was conducted to detect ENO1 in TNBC cells under hypoxia after treatment with BafA1 and exosomes containing Lamp2a-TAT/TRA-ligand 2. LTRL: Lamp2a-TAT/TRA-ligand 2. **B** A protein stability assay was conducted to determine ENO1 protein stability in TNBC cells under hypoxia after treatment with BafA1 and exosomes containing Lamp2a-TAT/TRA-ligand 2. **C** Lamp2a-deleted TAT and TRA-ligand 2 plasmids were transfected into ADSCs to generate NC-TAT/TRA-ligand 2. A Western blot analysis was conducted to detect ENO1 in TNBC cells under hypoxia after treatment with exosomes containing NC-TAT/TRA-ligand 2 or Lamp2a-TAT/TRA-ligand 2. **D** Both NC-TAT/TRA-ligand 2 and Lamp2a-TAT/TRA-ligand 2 were confirmed to bind to the ENO1 protein via a RIP assay. **E** In TNBC cells with endogenous Lamp2a knockdown, Lamp2a can be detected to bind the ENO1 protein via a co-IP assay after treatment with exosomes containing the Lamp2a-TAT/TRA-ligand 2 complex. **F** Immunofluorescence revealed that exosomes expressing the Lamp2a-TAT/TRA-ligand 2 complex reduced ENO1 protein levels and induced the colocalization of the ENO1 protein with lysosomes. Data represent mean ± SEM (*n* = 5 for all assays), unpaired Student’s *t* test for comparisons involving two treatment groups, or a one-way ANOVA for comparisons involving more than two groups.

BafA1 was added to TNBC cells to determine whether exosomes containing Lamp2a-TAT/TRA-ligand 2 indeed induce ENO1 degradation in lysosomes. Western blot analysis revealed that the reduction in ENO1 induced by exosomes containing Lamp2a-TAT/TRA-ligand 2 was reversed by BafA1 in TNBC cells under hypoxia (Fig. 8A). In the protein stability assay, compared with control exosomes, exosomes with Lamp2a-TAT/TRA-ligand 2 accelerated the degradation of ENO1. However, this effect was suppressed by BafA1 (Fig. 8B). To further assess whether Lamp2a in the protein‒RNA complex is the key factor that induces ENO1 degradation in lysosomes, we transfected Lamp2a-deleted TAT and TRA-ligand 2 plasmids into ADSCs to generate NC-TAT/TRA-ligand 2. Compared with exosomes with Lamp2a-TAT/TRA-ligand 2, those with NC-TAT/TRA-ligand 2 had a minor effect on the ENO1 protein level (Fig. 8C). Both NC-TAT/TRA-ligand 2 and Lamp2a-TAT/TRA-ligand 2 were confirmed to bind to the ENO1 protein via a RIP assay (Fig. 8D). In TNBC cells with endogenous Lamp2a knockdown, Lamp2a binding to the ENO1 protein was detected via a Co-IP assay after treatment with exosomes containing Lamp2a-TAT/TRA-ligand 2 (Fig. 8E). In contrast, Lamp2a did not bind the ENO1 protein after treatment with exosomes containing the NC-TAT/TRA-ligand 2. Exosomes with Lamp2a-TAT/TRA-ligand 2 reduced the ENO1 protein level and induced the colocalization of the ENO1 protein with lysosomes, as shown by immunofluorescence (Fig. 8F). BafA1 prevented the reduction in ENO1 protein, while most ENO1 proteins were observed in lysosomes. These data collectively indicate that Lamp2a-TAT/TRA-ligand 2 binds to ENO1 via ligand 2 and further induces ENO1 degradation in lysosomes.

Compared with control exosome treatment, treatment with exosomes containing Lamp2a-TAT/TRA-ligand 2 suppressed the viability (Supplementary Fig. 11A), invasion (Supplementary Fig. 11B) and colony formation (Supplementary Fig. 11C) of TNBC cells. In addition, ENO1 enzymatic activity, PEP levels, the glycoPER and lactate levels were decreased by Lamp2a-TAT/TRA-ligand 2 under hypoxia (Supplementary Fig. 11D–G). To functionally validate the on-target action of our engineered exosomes, we performed a comprehensive rescue assay in ENO1-knockdown (ENO1-KD) TNBC cells. MDA-MB-231 and MDA-MB-468 cells were first transfected with control or ENO1-specific shRNAs to establish ENO1-KD cell lines. These ENO1-KD cells were then transiently reconstituted with plasmids expressing either wild-type ENO1 (WT) or the RNA-binding-deficient K343R mutant. As expected, ENO1-KD + NC-Exo cells showed a significant decrease in cell viability, invasion, and colony formation compared to the KD-NC + NC-Exo control group, confirming the essential role of ENO1 in maintaining malignant behavior (Supplementary Fig. 10D, E). Reconstitution with either ENO1-WT or ENO1-K343R effectively restored these malignant phenotypes after treatment with control exosomes, indicating that both ENO1 variants can functionally compensate for the loss of endogenous ENO1. Critically, treatment with Lig2-Exo (exosomes containing Lamp2a-TAT/TRA-ligand 2) significantly inhibited the rescued malignant phenotypes in cells reconstituted with ENO1-WT, reducing the viability, invasion, and colony formation to levels comparable to the ENO1-KD group. This demonstrates that the functional exosome complex requires its RNA ligand to bind its target. In stark contrast, Lig2-Exo had no significant inhibitory effect on the malignant behaviors of cells reconstituted with the ENO1-K343R mutant. This demonstrates that the functional output of our therapeutic exosomes—their ability to suppress tumor cell malignancy—is strictly dependent on the capacity of their packaged ligand to bind ENO1. Since the K343R mutant cannot be bound by the ligand, the entire therapeutic mechanism (binding, Lamp2a-mediated trafficking, and degradation) is disrupted. The complete lack of effect in this genetic background provides powerful functional evidence that the action of our exosome-based therapy is exquisitely on-target and specific, ruling out major off-target mechanisms.

**Fig. 9:**
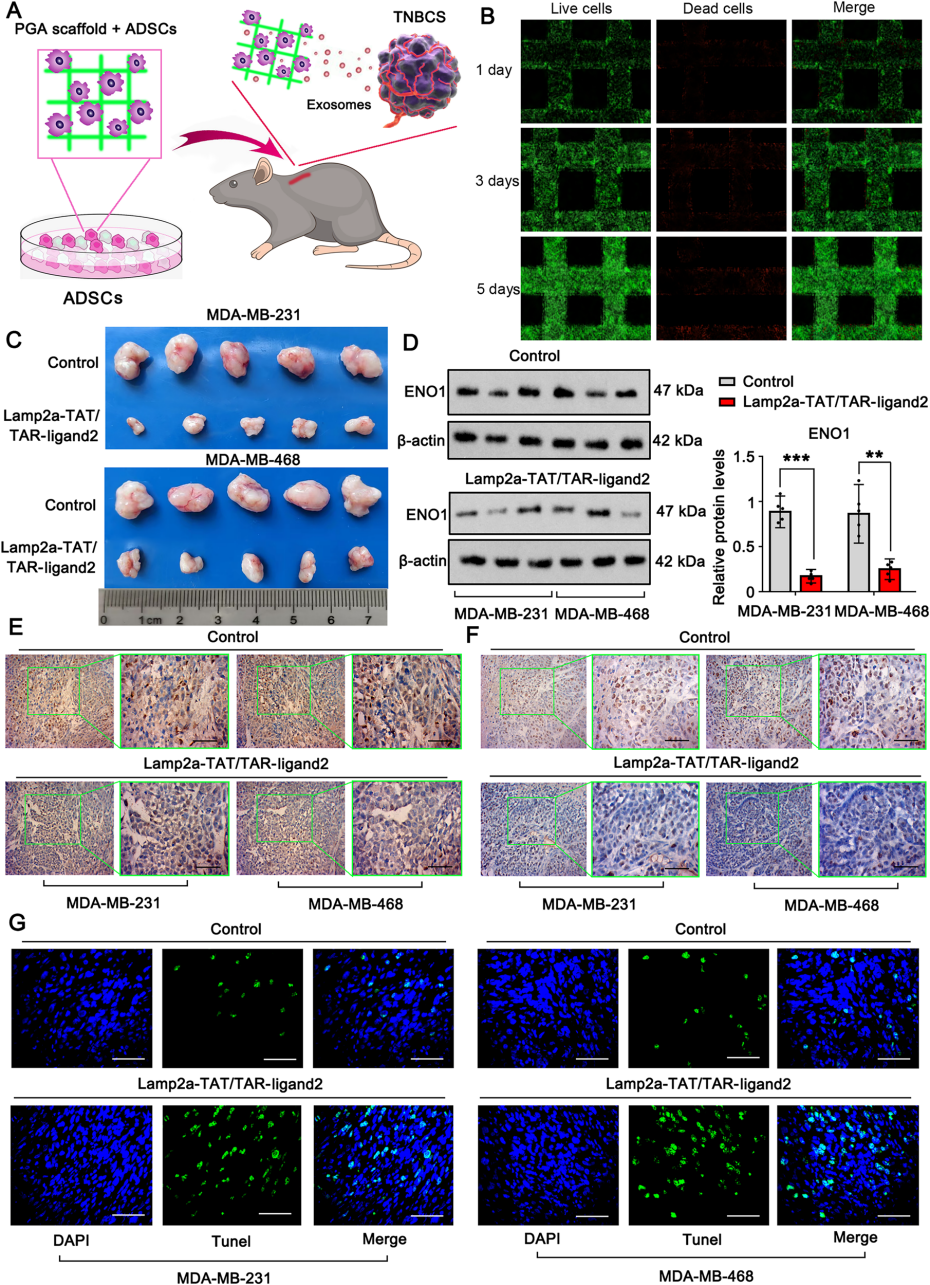
The Lamp2a-linked RNA ligand suppresses TNBC malignant behavior in vivo*.* **A** Schematic diagram of the in vivo study. ADSCs were initially seeded in the PGA scaffold in the culture medium. ADSCs together with the PGA scaffold were implanted subcutaneously into the backs of nude mice, and TNBC cells were subcutaneously injected at the same location. **B** Live/dead cell assays revealed that ADSCs that adhered to the PGA scaffold grew rapidly from day 1 to day 5, with minor cell death. **C** ADSCs with Lamp2a-TAT/TRA-ligand 2 suppressed the growth of xenografted TNBC tumors. ENO1 protein levels in TNBC tumors were decreased by ADSCs expressing Lamp2a-TAT/TRA-ligand 2, as indicated by Western blotting (**D**) and immunohistochemistry (**E**). Western blotting show three representative samples. Immunohistochemistry, Scale bar: 50 μm. **F**, **G** Evaluation of cell proliferation and apoptosis via Ki67 and TUNEL immunohistochemistry, respectively. Scale bar: 50 μm. Data represent mean ± SEM (*n* = 5 for all assays); **p < 0.01, ***p < 0.001 (unpaired Student’s *t* test for comparisons involving two treatment groups).

For in vivo studies, ADSCs were initially seeded in the PGA scaffold and culture medium. ADSCs have poor adhesion ability. The PGA scaffold was previously developed to improve the adhesion of ADSCs. ADSCs and the PGA scaffold were implanted subcutaneously into the backs of the nude mice, which received a subcutaneous injection of TNBC cells at the same location (Fig. 9A). Owing to the adhesion of ADSCs to the PGA scaffold, the ADSCs gathered near the TNBC cells so that the ADSC-secreted exosomes could easily reach and continuously impact the TNBC cells. Live/dead cell assays revealed that ADSCs that adhered to the PGA scaffold grew rapidly from day 1 to day 5, with minor cell death (Fig. 9B). ADSCs expressing Lamp2a-TAT/TRA-ligand 2 suppressed the growth of xenograft TNBC tumors (Fig. 9C). ENO1 protein levels in TNBC tumors were decreased by ADSCs expressing Lamp2a-TAT/TRA-ligand 2, as indicated by Western blotting and immunohistochemistry (Fig. 9D, E). The Ki67 level also decreased with increasing TUNEL staining (Fig. 9F, G).

## Discussion

Glycolysis is a multistep metabolic pathway that occurs in the cytosol; it involves the breakdown of one molecule of glucose to generate two net molecules of ATP, independent of molecular oxygen availability. When oxygen is limited, pyruvate in the cytosol is converted into L-lactate by lactate dehydrogenase A (LDHA), and NADH is oxidized to NAD^+^. Recent studies have revealed that lactate, in addition to being an energy source and metabolic byproduct, can induce an epigenetic modification, lactylation [23]. A global lactylome analysis of hepatitis B virus-related hepatocellular carcinoma tissues revealed that genes involved in metabolic pathways, including the TCA cycle and carbohydrate, amino acid, fatty acid, and nucleotide metabolism, are preferentially affected by histone lactylation [32]. Like many other cancers, TNBCs undergo metabolic reprogramming, shifting toward a highly glycolytic profile through the Warburg effect [33]. Emerging evidence has demonstrated that LDHA expression is significantly elevated in TNBC tumors compared with non-TNBC tumors and is associated with shorter overall and disease-free survival [34], as well as TNBC-related lactate acidosis [35]. Therefore, gaining a deeper understanding of the mechanisms underlying glycolysis, particularly lactate-related lactylation, may reveal new avenues for TNBC treatment and offer a promising strategy to prevent the aggressive progression of this type of tumor.

In a process two steps away from pyruvate production during glycolysis, 2-PG is converted to PEP in the presence of ENO1. ENO1 is well recognized for its role in promoting metastasis in various types of cancer, including gastric cancer [36], cervical cancer [37], pancreatic cancer [38, 39], and skin cutaneous melanoma [40]. Consistent with these findings, our current study revealed an association between ENO1 expression and the aggressiveness of breast cancer. Notably, ENO1 expression is significantly higher in more aggressive TNBC tumors than in non-TNBC tumors. In TNBC samples, we found that *ENO1* mRNA levels are lowest in the MSL subtype and highest in the BL subtype of TNBC. Interestingly, compared with other breast cancer subtypes, TNBC is more reliant on glycolysis, exhibiting increased glucose uptake and lactate secretion [41]. TNBCs can be further subclassified into three subclusters, MPS1, MPS2, and MPS3, on the basis of their metabolic features [42]. Among these subclusters, MPS2 is often referred to as the glycolytic subtype because of its significant upregulation of carbohydrate and nucleotide metabolism pathways. Notably, MPS2 TNBC is predominantly composed of classical basal-like cases and has the highest frequency of PAM50 basal subtype cases. Compared with the other two subtypes of TNBC, MPS2 patients also exhibit significantly worse relapse-free survival [42]. Our current study reveals a novel feedback loop in which ENO1-mediated glycolysis produces lactate, stabilizes the ENO1 protein through specific lysine residue lactylation, and further promotes glycolytic metabolism and the capacity of the cell to adapt to a hypoxic environment. Given the central role of glycolysis in tumor progression [43], exploring the global lactylome landscape in TNBC tumors would be intriguing.

TNBC invasiveness is strongly associated with the hypoxic microenvironment in which TNBC cells reside. Cancer cells often induce localized hypoxia to promote angiogenesis [44, 45]. Additionally, hypoxia activates the hypoxia-inducible factor (HIF)-mediated signaling pathway, leading to the transcription of genes associated with angiogenesis, glycolysis, and apoptosis and the infiltration of tumor-associated immune cells [46]. Consequently, ENO1, the key intermediate enzyme in glycolysis, has a particular important role in enabling TNBC cells to thrive in a hypoxic environment. One of the intriguing findings of this study is that ENO1 inactivation has a stronger inhibitory effect on various TNBC cell properties, including proliferation, invasion, and colony formation, under hypoxic conditions than under normoxic conditions. These findings indicate that glycolysis is the primary metabolic pathway providing energy and building blocks for cell division under hypoxia, which closely resembles the physiological tumor microenvironment. Therefore, interventions targeting ENO1 may effectively exploit the metabolic vulnerability of TNBC in the in vivo tumor microenvironment.

Lactylation can be achieved through either enzymatic or nonenzymatic transfer of the lactyl group from lactylcoenzyme A (lactyl-CoA) to lysine residues. Enzymatic lysine lactylation relies on the lysine acetyltransferase enzyme EP300. Indeed, the levels of lactylated ENO1 are substantially reduced in the absence of EP300. Importantly, the site of the lysine residue plays a crucial role in ENO1 lactylation, as demonstrated by our data, indicating that K262 is indispensable for this modification. A mutation at this residue markedly decreased the abundance of lactylated ENO1, even under conditions of EP300 overexpression. Du et al. Identified SIRT1/SIRT3 as primary erasers of ENO1 K228 lactylation in HepG2 hepatocellular carcinoma cells [29]. Our study showed that K228 mutation does not abolish EP300-mediated lactylation, indicating that this site is not essential for EP300-induced lactylation in our system. The differences between our findings and Du et al. primarily reflect distinct regulatory perspectives (EP300-mediated lactylation induction vs. SIRT1/SIRT3-mediated delactylation).

Our study identified K262 as the primary site for EP300-mediated lactylation, and mutation of this site (K262R) led to accelerated lysosomal degradation of ENO1 even under lactate-rich conditions. This suggests that the modification state of K262 is a critical determinant of ENO1 stability by diverting it from lysosomal degradation. An intriguing question arising from this finding is the precise molecular mechanism by which lactylation impedes lysosomal localization. While the exact pathway warrants further investigation, we propose a plausible model based on the nature of the lactylation modification. It is well-established that specific types of ubiquitin chain linkages direct proteins to distinct degradation pathways. In particular, K48-linked ubiquitination primarily targets substrates for proteasomal degradation, whereas K63-linked ubiquitination has been strongly implicated in directing protein cargo to the lysosome for degradation [47, 48]. We hypothesize that the addition of a lactyl group to the epsilon-amino group of K262 would sterically hinder the enzymatic addition of a ubiquitin chain, specifically the K63-linked chain, to the same residue. This competitive inhibition would disrupt the essential recognition signal required for the lysosomal trafficking machinery. Future studies aimed at identifying the specific lysosomal degradation pathway for ENO1 and mapping the exact interaction interfaces will be crucial to validate this hypothesis.

It is important to note that while C646 effectively inhibited lactylation, as a general EP300 inhibitor, it would also be expected to concurrently suppress other EP300-dependent acyl modifications, such as the previously reported 2-hydroxyisobutyrylation of ENO1 [28]. Therefore, future studies are warranted to further dissect the individual contributions of lactylation versus other EP300-mediated modifications, such as 2-hydroxyisobutyrylation, to ENO1’s function. This could involve the development of tools capable of selectively inhibiting a single type of acylation on ENO1.

In recent years, targeted protein degradation (TPD) technology, which depends on two major protein degradation systems, the ubiquitin proteasome system and lysosomal system, has developed rapidly, especially in the fields of cancer, chronic diseases and rare diseases [49]. This technology first induces the degradation of a targeted protein through a ligand that specifically binds to the target protein. The ligand can be an antibody and a nucleic acid aptamer (i.e., a short RNA or DNA oligonucleotide). The ligand is linked to proteins that induce degradation via the ubiquitin–proteasome system or lysosomal system. This study revealed that ENO1 in TNBC primarily undergoes lysosomal degradation rather than ubiquitin‒proteasome-mediated degradation. However, lactylation prevents the degradation of ENO1 in lysosomes, thereby increasing ENO1 levels in TNBC. ENO1 not only is a glycolytic enzyme but also functions as an RNA-binding protein [9]. Huppertz et al. reported that human ENO1 binds hundreds of mRNAs via specific binding regions, such as “TTTTTTBTTTTTT” (the letter B represents T, G or C) and “CCCAGRC” (the letter R represents G or A) [9]. Synthetic RNA ligands corresponding to these regions inhibit ENO1’s enzymatic activity [9]. Exploiting this feature of ENO1, we also designed RNA ligands to bind ENO1 proteins. The RNA ligands were linked to the Lamp2a protein. Lamp2a is a key protein that mediates autophagic lysosome degradation, as knockdown of Lamp2a notably impairs this process [30]. Chaperone-mediated autophagy (CMA) is a selective form of autophagy that targets proteins for lysosomal degradation [49]. To achieve this, a KFERQ motif (Lys-Phe-Glu-Arg-Gln) present on the target proteins is recognized by the intracellular chaperone protein HSPA8, and the complexes are then transported into the lysosome via Lamp2a [50]. Although ENO1 does not contain the KFERQ motif, we added the KFERQ peptide to Lamp2a-TAT so that Lamp2a-TAT/TRA-ligand-ENO1 complexes could be successfully transported to the lysosome for degradation.

In this study, ADSCs were induced to continually generate Lamp2a-TAT/TRA-ligand 2 complexes. Lamp2a regulates the loading of proteins into MVBs that fuse with the plasma membrane, leading to the release of mature exosomes [51]. Sutaria et al. linked miR-199a with Lamp2a to increase the level of miR-199a in exosomes [8]. In this study, Lamp2a-TAT/TRA-ligand 2 complexes were detected in exosomes released by ADSCs. Exosomes delivered these compounds to recipient TNBC cells for ENO1 degradation. Notably, ADSCs have poor adhesion ability. Untethered ADSCs can move far away from the TNBC cells, so ADSC-secreted exosomes could not be taken up by TNBC cells. Therefore, in this study, ADSCs were seeded in a PGA scaffold that was developed to facilitate the adhesion of stem cells [52]. ADSCs adhering to the PGA scaffold secreted exosomes that effectively inhibited the glycolysis and malignant behaviors of TNBC cells.

This new TPD technology involving ADSCs has several advantages for the treatment of TNBC. First, in theory, ADSCs can permanently secrete exosomes containing Lamp2a-TAT/TRA-ligand 2. Therefore, intervention at regular intervals, such as with other traditional methods, such as chemotherapy, targeted therapy with small molecules and immunotherapy, might be unnecessary. Second, a PGA scaffold with seeded ADSCs was implanted near TNBC cells in vivo. As this treatment is confined to tumor tissues, it prevents extensive side effects caused by the abovementioned systemic therapies. Third, ADSCs can be easily obtained from patients for subsequent genetic modification. Patients theoretically have no rejection response of autologous ADSCs. All these advantages should be further validated in more preclinical studies.

**Fig. 10:**
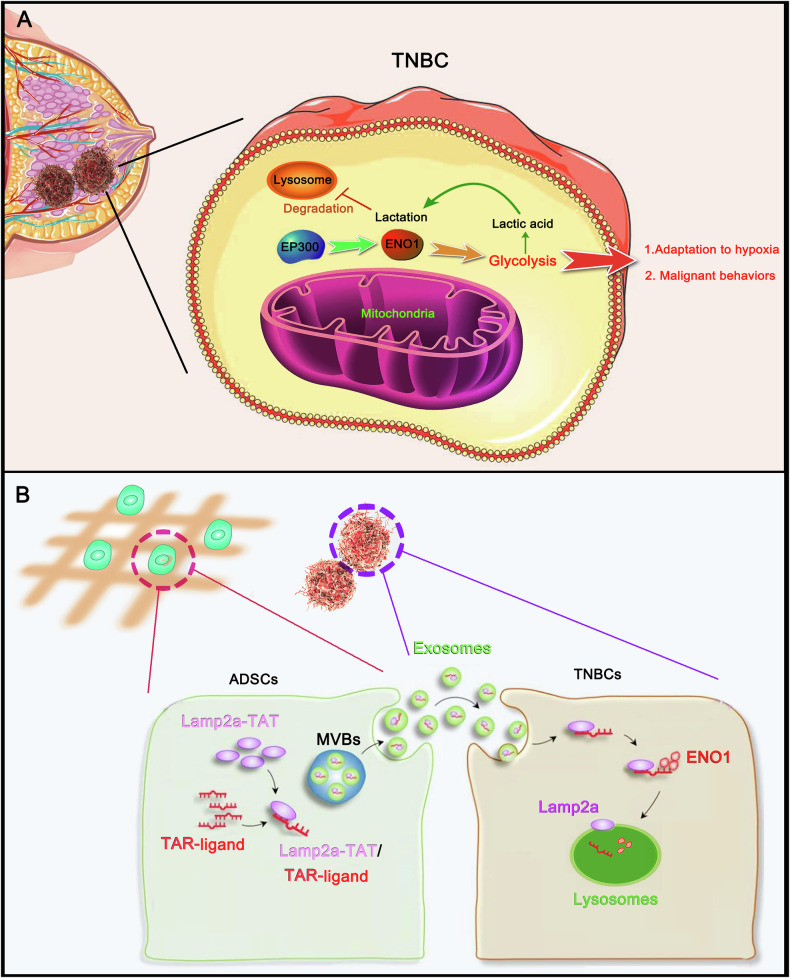
Schematic diagrams showing the effects of ENO1 lactylation on the malignant behaviors of TNBC cells and showing that the Lamp2a-linked RNA ligand induces ENO1 degradation in lysosomes. **A** Lactate-induced lysine lactylation of the ENO1 protein plays vital roles in the growth and metastasis of TNBC tumors. Lactylation increased ENO1 protein stability and enzyme activity, which intensified the glycolytic pathway. Notably, as lactate levels increased, a positive feedback loop was established, further promoting lactylation of ENO1. This positive feedback mechanism enables TNBC cells to adapt more efficiently to hypoxia and enhances their malignant behaviors. **B** A strategy for inducing ENO1 degradation based on the RNA-binding protein feature of ENO1. In this study, an RNA ligand that can be specifically bind ENO1 was identified. The RNA ligand was linked to the Lamp2a protein in adipose-derived stem cells (ADSCs) after stable transfection with Lamp2a-TAT and TRA-ligand plasmids. ADSCs seeded in the PGA scaffold secreted exosomes containing the Lamp2a-linked ligand. After the complex enters TNBC cells, it binds to ENO1 via the RNA ligand and further induces the lysosomal degradation of ENO1 by the Lamp2a protein, a key protein that mediates lysosomal degradation.

In summary, our study revealed that lactylation increased ENO1 protein stability and enzyme activity, which promoted glycolysis. The schematic diagrams are shown in Fig. 10A. Notably, as lactate levels increased, a positive feedback loop was established, further promoting lactylation of ENO1. This positive feedback mechanism enables TNBC cells to adapt more efficiently to hypoxia and enhances their malignant behaviors. We developed a TPD approach involving the expression of Lamp2a-TAT/TRA-ligand 2 in ADSCs adhering to a PGA scaffold. The schematic diagrams are shown in Fig. 10B. The TPD approach effectively inhibited glycolysis and malignant cell behaviors in TNBC cells by inducing ENO1 degradation in lysosomes. Therefore, this study provides crucial insights into the mechanism by which ENO1 lactylation mediates glycolysis to facilitate TNBC cell adaptation to hypoxic conditions and provides a strategy for targeting ENO1.

## Supplementary information

Supplementary materials, methods and Figures

Supplementary Table 1

Supplementary Table 2

Supplementary Table 3

Supplementary Table 4

Supplementary Table 5

Full WB

## Data Availability

The datasets generated/analyzed in the present study are available upon reasonable request from the corresponding author.

## References

[1] Rakha EA, Tse GM, Quinn CM. An update on the pathological classification of breast cancer. Histopathology. 2023;82:5–16. 36482272 10.1111/his.14786 PMC10108289

[2] Chandel NS. Glycolysis. Cold Spring Harb Perspect Biol. 2021;13:10. 10.1101/cshperspect.a040618 PMC8485748 34598925

[3] Ghanbari Movahed Z, Rastegari-Pouyani M, Mohammadi MH, Mansouri K. Cancer cells change their glucose metabolism to overcome increased ROS: one step from cancer cell to cancer stem cell?. Biomed Pharmacother. 2019;112:108690. 30798124 10.1016/j.biopha.2019.108690

[4] Rogatzki MJ, Ferguson BS, Goodwin ML, Gladden LB. Lactate is always the end product of glycolysis. Front Neurosci. 2015;9:22. 25774123 10.3389/fnins.2015.00022 PMC4343186

[5] Jin J, Bai L, Wang D, Ding W, Cao Z, Yan P, et al. SIRT3-dependent delactylation of cyclin E2 prevents hepatocellular carcinoma growth. EMBO Rep. 2023;24:e56052. 36896611 10.15252/embr.202256052 PMC10157311

[6] Pancholi V. Multifunctional alpha-enolase: its role in diseases. Cell Mol Life Sci. 2001;58:902–20. 11497239 10.1007/PL00000910 PMC11337373

[7] Qiao G, Wu A, Chen X, Tian Y, Lin X. Enolase 1, a moonlighting protein, as a potential target for cancer treatment. Int J Biol Sci. 2021;17:3981–92. 34671213 10.7150/ijbs.63556 PMC8495383

[8] Sutaria DS, Jiang J, Elgamal OA, Pomeroy SM, Badawi M, Zhu X, et al. Low active loading of cargo into engineered extracellular vesicles results in inefficient miRNA mimic delivery. J Extracell Vesicles. 2017;6:1333882. 28717424 10.1080/20013078.2017.1333882 PMC5505005

[9] Huppertz I, Perez-Perri JI, Mantas P, Sekaran T, Schwarzl T, Russo F, et al. Riboregulation of Enolase 1 activity controls glycolysis and embryonic stem cell differentiation. Mol Cell. 2022;82:2666–80. 35709751 10.1016/j.molcel.2022.05.019

[10] Maire V, Nemati F, Richardson M, Vincent-Salomon A, Tesson B, Rigaill G, et al. Polo-like kinase 1: a potential therapeutic option in combination with conventional chemotherapy for the management of patients with triple-negative breast cancer. Cancer Res. 2013;73:813–23. 23144294 10.1158/0008-5472.CAN-12-2633

[11] Maire V, Baldeyron C, Richardson M, Tesson B, Vincent-Salomon A, Gravier E, et al. TTK/hMPS1 is an attractive therapeutic target for triple-negative breast cancer. PLoS ONE. 2013;8:e63712. 23700430 10.1371/journal.pone.0063712 PMC3658982

[12] Maubant S, Tesson B, Maire V, Ye M, Rigaill G, Gentien D, et al. Transcriptome analysis of Wnt3a-treated triple-negative breast cancer cells. PLoS ONE. 2015;10:e0122333. 25848952 10.1371/journal.pone.0122333 PMC4388387

[13] Jezequel P, Loussouarn D, Guerin-Charbonnel C, Campion L, Vanier A, Gouraud W, et al. Gene-expression molecular subtyping of triple-negative breast cancer tumours: importance of immune response. Breast Cancer Res. 2015;17:43. 25887482 10.1186/s13058-015-0550-y PMC4389408

[14] Jezequel P, Kerdraon O, Hondermarck H, Guerin-Charbonnel C, Lasla H, Gouraud W, et al. Identification of three subtypes of triple-negative breast cancer with potential therapeutic implications. Breast Cancer Res. 2019;21:65. 31101122 10.1186/s13058-019-1148-6 PMC6525459

[15] Burstein MD, Tsimelzon A, Poage GM, Covington KR, Contreras A, Fuqua SA, et al. Comprehensive genomic analysis identifies novel subtypes and targets of triple-negative breast cancer. Clin Cancer Res. 2015;21:1688–98. 25208879 10.1158/1078-0432.CCR-14-0432 PMC4362882

[16] den Hollander P, Rawls K, Tsimelzon A, Shepherd J, Mazumdar A, Hill J, et al. Phosphatase PTP4A3 promotes triple-negative breast cancer growth and predicts poor patient survival. Cancer Res. 2016;76:1942–53. 26921331 10.1158/0008-5472.CAN-14-0673 PMC4873402

[17] Goldman MJ, Craft B, Hastie M, Repecka K, McDade F, Kamath A, et al. Visualizing and interpreting cancer genomics data via the Xena platform. Nat Biotechnol. 2020;38:675–8. 32444850 10.1038/s41587-020-0546-8 PMC7386072

[18] Curtis C, Shah SP, Chin SF, Turashvili G, Rueda OM, Dunning MJ, et al. The genomic and transcriptomic architecture of 2,000 breast tumours reveals novel subgroups. Nature. 2012;486:346–52. 22522925 10.1038/nature10983 PMC3440846

[19] Duffy MJ, Harbeck N, Nap M, Molina R, Nicolini A, Senkus E, et al. Clinical use of biomarkers in breast cancer: updated guidelines from the European Group on Tumor Markers (EGTM). Eur J Cancer. 2017;75:284–98. 28259011 10.1016/j.ejca.2017.01.017

[20] Alluri P, Newman LA. Basal-like and triple-negative breast cancers: searching for positives among many negatives. Surg Oncol Clin N Am. 2014;23:567–77. 24882351 10.1016/j.soc.2014.03.003 PMC4304394

[21] Lehmann BD, Bauer JA, Chen X, Sanders ME, Chakravarthy AB, Shyr Y, et al. Identification of human triple-negative breast cancer subtypes and preclinical models for selection of targeted therapies. J Clin Invest. 2011;121:2750–67. 21633166 10.1172/JCI45014 PMC3127435

[22] Yang T, Shu X, Zhang HW, Sun LX, Yu L, Liu J, et al. Enolase 1 regulates stem cell-like properties in gastric cancer cells by stimulating glycolysis. Cell Death Dis. 2020;11:870. 33067426 10.1038/s41419-020-03087-4 PMC7567818

[23] Chen W, Zhang Z, Chang C, Yang Z, Wang P, Fu H, et al. A bioenergetic shift is required for spermatogonial differentiation. Cell Discov. 2020;6:56. 32864161 10.1038/s41421-020-0183-x PMC7431567

[24] Zhang D, Tang Z, Huang H, Zhou G, Cui C, Weng Y, et al. Metabolic regulation of gene expression by histone lactylation. Nature. 2019;574:575–80. 31645732 10.1038/s41586-019-1678-1 PMC6818755

[25] Yang K, Fan M, Wang X, Xu J, Wang Y, Tu F, et al. Lactate promotes macrophage HMGB1 lactylation, acetylation, and exosomal release in polymicrobial sepsis. Cell Death Differ. 2022;29:133–46. 34363018 10.1038/s41418-021-00841-9 PMC8738735

[26] Schneider-Poetsch T, Ju J, Eyler DE, Dang Y, Bhat S, Merrick WC, et al. Inhibition of eukaryotic translation elongation by cycloheximide and lactimidomycin. Nat Chem Biol. 2010;6:209–17. 20118940 10.1038/nchembio.304 PMC2831214

[27] Huang H, Tang S, Ji M, Tang Z, Shimada M, Liu X, et al. p300-mediated lysine 2-hydroxyisobutyrylation regulates glycolysis. Mol Cell. 2018;70:663–78. 29775581 10.1016/j.molcel.2018.04.011 PMC6029451

[28] Bowers EM, Yan G, Mukherjee C, Orry A, Wang L, Holbert MA, et al. Virtual ligand screening of the p300/CBP histone acetyltransferase: identification of a selective small molecule inhibitor. Chem Biol. 2010;17:471–82. 20534345 10.1016/j.chembiol.2010.03.006 PMC2884008

[29] Du R, Gao Y, Yan C, Ren X, Qi S, Liu G, et al. Sirtuin 1/sirtuin 3 are robust lysine delactylases and sirtuin 1-mediated delactylation regulates glycolysis. iScience. 2024;27:110911. 39351192 10.1016/j.isci.2024.110911 PMC11440250

[30] Issa AR, Sun J, Petitgas C, Mesquita A, Dulac A, Robin M, et al. The lysosomal membrane protein LAMP2A promotes autophagic flux and prevents SNCA-induced Parkinson disease-like symptoms in the Drosophila brain. Autophagy. 2018;14:1898–910. 29989488 10.1080/15548627.2018.1491489 PMC6152503

[31] Hamilton G, Teufelsbauer M. Adipose-derived stromal/stem cells and extracellular vesicles for cancer therapy. Expert Opin Biol Ther. 2022;22:67–78. 34236014 10.1080/14712598.2021.1954156

[32] Yang Z, Yan C, Ma J, Peng P, Ren X, Cai S, et al. Lactylome analysis suggests lactylation-dependent mechanisms of metabolic adaptation in hepatocellular carcinoma. Nat Metab. 2023;5:61–79. 36593272 10.1038/s42255-022-00710-w

[33] Naik A, Decock J. Lactate metabolism and immune modulation in breast cancer: a focused review on triple negative breast tumors. Front Oncol. 2020;10:598626. 33324565 10.3389/fonc.2020.598626 PMC7725706

[34] Huang X, Li X, Xie X, Ye F, Chen B, Song C, et al. High expressions of LDHA and AMPK as prognostic biomarkers for breast cancer. Breast. 2016;30:39–46. 27598996 10.1016/j.breast.2016.08.014

[35] Warner E. Type B lactic acidosis and metastatic breast cancer. Breast Cancer Res Treat. 1992;24:75–9. 1463874 10.1007/BF01832361

[36] Qian X, Xu W, Xu J, Shi Q, Li J, Weng Y, et al. Enolase 1 stimulates glycolysis to promote chemoresistance in gastric cancer. Oncotarget. 2017;8:47691–708. 28548950 10.18632/oncotarget.17868 PMC5564598

[37] Gou Y, Li F, Huo X, Hao C, Yang X, Pei Y, et al. ENO1 monoclonal antibody inhibits invasion, proliferation and clone formation of cervical cancer cells. Am J Cancer Res. 2021;11:1946–61. 34094663 PMC8167678

[38] Principe M, Borgoni S, Cascione M, Chattaragada MS, Ferri-Borgogno S, Capello M, et al. Alpha-enolase (ENO1) controls alpha v/beta 3 integrin expression and regulates pancreatic cancer adhesion, invasion, and metastasis. J Hematol Oncol. 2017;10:16. 28086938 10.1186/s13045-016-0385-8 PMC5237223

[39] Li Y, Li Y, Luo J, Fu X, Liu P, Liu S, et al. FAM126A interacted with ENO1 mediates proliferation and metastasis in pancreatic cancer via PI3K/AKT signaling pathway. Cell Death Discov. 2022;8:248. 35513377 10.1038/s41420-022-01047-9 PMC9072533

[40] Zhang K, Tian R, Zhang W, Li Y, Zeng N, Liang Y, et al. alpha-Enolase inhibits apoptosis and promotes cell invasion and proliferation of skin cutaneous melanoma. Mol Biol Rep. 2022;49:8241–50. 35925486 10.1007/s11033-022-07540-9 PMC9463226

[41] Wang Z, Jiang Q, Dong C. Metabolic reprogramming in triple-negative breast cancer. Cancer Biol Med. 2020;17:44–59. 32296576 10.20892/j.issn.2095-3941.2019.0210 PMC7142847

[42] Gong Y, Ji P, Yang YS, Xie S, Yu TJ, Xiao Y, et al. Metabolic-pathway-based subtyping of triple-negative breast cancer reveals potential therapeutic targets. Cell Metab. 2021;33:51–64 e59. 33181091 10.1016/j.cmet.2020.10.012

[43] Zhou D, Duan Z, Li Z, Ge F, Wei R, Kong L. The significance of glycolysis in tumor progression and its relationship with the tumor microenvironment. Front Pharmacol. 2022;13:1091779. 36588722 10.3389/fphar.2022.1091779 PMC9795015

[44] Muz B, de la Puente P, Azab F, Azab AK. The role of hypoxia in cancer progression, angiogenesis, metastasis, and resistance to therapy. Hypoxia. 2015;3:83–92. 27774485 10.2147/HP.S93413 PMC5045092

[45] Zhang Y, Zhang H, Wang M, Schmid T, Xin Z, Kozhuharova L, et al. Hypoxia in breast cancer-scientific translation to therapeutic and diagnostic clinical applications. Front Oncol. 2021;11:652266. 33777815 10.3389/fonc.2021.652266 PMC7991906

[46] Srivastava N, Usmani SS, Subbarayan R, Saini R, Pandey PK. Hypoxia: syndicating triple negative breast cancer against various therapeutic regimens. Front Oncol. 2023;13:1199105. 37492478 10.3389/fonc.2023.1199105 PMC10363988

[47] Dores MR, Trejo J. Endo-lysosomal sorting of G-protein-coupled receptors by ubiquitin: diverse pathways for G-protein-coupled receptor destruction and beyond. Traffic. 2019;20:101–9. 10.1111/tra.12619. 30353650 10.1111/tra.12619 PMC6385894

[48] Berlin I, Sapmaz A, Stévenin V, Neefjes J. Ubiquitin and its relatives as wizards of the endolysosomal system. J Cell Sci. 2023;136:jcs260101. 36825571 10.1242/jcs.260101 PMC10022685

[49] Dale B, Cheng M, Park KS, Kaniskan HÜ, Xiong Y, Jin J. Advancing targeted protein degradation for cancer therapy. Nat Rev Cancer. 2021;21:638–54. 34131295 10.1038/s41568-021-00365-x PMC8463487

[50] Zhao X, Di Q, Yu J, Quan J, Xiao Y, Zhu H, et al. USP19 (ubiquitin specific peptidase 19) promotes TBK1 (TANK-binding kinase 1) degradation via chaperone-mediated autophagy. Autophagy. 2022;18:891–908. 34436957 10.1080/15548627.2021.1963155 PMC9037486

[51] Ferreira JV, da Rosa Soares A, Ramalho J, Máximo Carvalho C, Cardoso MH, Pintado P, et al. LAMP2A regulates the loading of proteins into exosomes. Sci Adv. 2022;8:eabm1140. 35333565 10.1126/sciadv.abm1140 PMC8956266

[52] Wang Y, Wang W, Wang X, Wang Y, Wang J, Fu Q, et al. Tissue-engineered sling with adipose-derived stem cells under static mechanical strain. Exp Ther Med. 2017;14:1337–42. 28810594 10.3892/etm.2017.4705 PMC5525904

