# Supplementary material for: A Lamp2a-linked RNA secreted by ADSCs prevents ENO1–lactylation–glycolysis feedback and cell malignant behavior in triple-negative breast cancer
Source: Cell Death Dis. 2026 Mar 2;17(1):288. doi: 10.1038/s41419-026-08517-3 (PMC13031273; doi:10.1038/s41419-026-08517-3)
Supplement: Supplementary file 1 — Supplementary materials, methods and Figures [file 41419_2026_8517_MOESM1_ESM.docx]

[Catalog](#_Toc22735)

[1 Materials and methods 1](#_Toc27330)

[1.1 Immunoprecipitation 1](#_Toc26758)

[1.2 Western blotting 2](#_Toc22869)

[1.3 Quantitative PCR (qPCR) 2](#_Toc31176)

[1.4 Immunofluorescence 3](#_Toc27710)

[1.5 Participants and Immunohistochemistry 3](#_Toc21535)

[1.6 Cell Counting Kit-8 (CCK8) assay 4](#_Toc28322)

[1.7 Transwell assay 4](#_Toc14548)

[1.8 Colony-formation assay 5](#_Toc7287)

[1.9 Lactate detection assay 5](#_Toc15509)

[1.10 Pyruvate Detection Assay 6](#_Toc1561)

[1.11 Phosphoenolpyruvate (PEP) Detection Assay 7](#_Toc19234)

[1.12 Enolase Activity Colorimetric Assay 8](#_Toc3487)

[1.13 RNA immunoprecipitation (RIP) 8](#_Toc26853)

[1.14 Exosome extraction 8](#_Toc28735)

[1.15 Fluorescence in situ hybridization (FISH) 9](#_Toc13346)

[1.16 Cell proliferation on scaffolds 9](#_Toc10839)

[1.17Terminal uridine nick-end labeling (TUNEL) assay 9](#_Toc21258)

[1.18 Statistical Analysis 10](#_Toc1067)

[2. Supplementary Figures 11](#_Toc12267)

2.1 Supplementary Figure 1 ....................................................................11 2.2 Supplementary Figure 2................................................................ 11

2.3 Supplementary Figure 3................................................................ 12

2.4 Supplementary Figure 4................................................................ 12

2.5 Supplementary Figure 5................................................................ 12

2.6 Supplementary Figure 6................................................................ 14

2.7 Supplementary Figure 7................................................................ 13

2.8 Supplementary Figure 8................................................................ 13

2.9 Supplementary Figure 9................................................................ 13

2.10 Supplementary Figure 10............................................................. 14

2.11 Supplementary Figure 11............................................................. 14

**1 Materials and methods**

**1.1 Immunoprecipitation**

Cells cultured in 10-cm dishes were initially rinsed with PBS subsequently lysed using 1ml of cell lysis buffer comprised 1% NP-40, 0.1% SDS, 50 mM DTT, 2 µg/ml Aprotinin, 2 µg /ml Leupeptin and 1 mM PMSF. The supernatant collected by centrifugation was added with 2 µg of the primary antibody and allowed to incubate at 4°C with gentle agitation overnight. The following day, the mixture was supplemented with 20 µl of resuspended Protein A+G Agarose beads and incubated at 4°C with gentle agitation for 2 hours. The resulting supernatant was carefully decanted after centrifugation at 2500 rpm for 5 minutes. The precipitate was subjected to five washes with the same cell lysis buffer and then resuspended in 20 µl of 1x SDS-PAGE electrophoresis sample loading buffer. Subsequently, the sample was heated in a boiling water bath for 5 minutes, rendering it suitable for subsequent SDS-PAGE electrophoresis and western blot analysis.

**1.2 Western blotting**

Total proteins were extracted from cells and tissues using the same RIPA buffer employed in the immunoprecipitation assay. Cell lysates or homogenized tissue slurry were incubated on ice for 30 minutes and then centrifuged at 10000 rpm for 15 minutes. The resulting supernatant, which contained the total protein, was collected and quantified using BCA Protein Assay Reagent (Bestbio, #BB-3401, China). Fifteen micrograms of each protein sample were loaded onto a 10% SDS-PAGE gel and separated through electrophoresis. Following the separation, the nylon membrane was blocked for non-specific binding by incubating it with 5% nonfat-dried milk for 1 hour at room temperature. The membrane was subsequently subjected to an overnight incubation with gentle agitation at 4°C in 5% nonfat-dried milk containing primary antibody. The primary antibodies used were shown in Supplementary Table 3. On the following day, the membrane was washed three times with PBS supplemented with 0.1% Tween20 (PBST), followed by a 1 hour-incubation with appropriate secondary antibodies at room temperature. Unbound secondary antibodies were removed by washing the membrane, and then HRP substrate (BestBio, #BB-3501, China) was added for chemiluminescent detection.

**1.3 Quantitative PCR (qPCR)**

Total RNA was extracted from both cells and tumors using TRIzol reagent (KeyGEN BioTECH, KGA1201, China) following the manufacturer’s instructions. Two micrograms of total RNA from each sample were utilized for cDNA synthesis with the First-strand cDNA synthesis kit from KeyGen BioTECH (KGA1316). Gene expression was then assessed by qPCR using Realtime PCR Master Mix (SYBR Green) from KeyGen BioTECH (KGA1339). The pimers employed for qPCR were shown in Supplementary Table2.

**1.4 Immunofluorescence**

For characterizing the intracellular localization of ENO1 and lysosomes, TNBC cells were cultured on coverslips until they reached 70% confluence. Lysosome staining was performed by adding pre-warmed medium containing 50 nM LysoTracker Red (Beyotime Co. Ltd., C1046, China) to the cells and incubating them at 37°C for 30 minutes. Subsequently, the cells were fixed with 4% paraformaldehyde at room temperature for 15 minutes, followed by permeabilization with 0.1% Tritox X-100 (Sigma-Aldrich, USA) at room temperature for 15 minutes. After a brief rinse with PBST, the cells were further incubated with PBS supplemented with 1% BSA (Cell Signaling Technology, USA) at room temperature for 1 hour. Following this, the cells were subjected to an overnight incubation with a primary antibody against ENO1 (Ptgcn, #11204-1-AP, 1:200) and lamp2 (Abcam, ab199946, 1:500) at 4°C. Unbound antibodies were removed by washing with PBS, and the cells were subsequently incubated with an Alexa Fluor 488-conjugated secondary antibody (1:1000, Thermo Fisher, USA) in the dark for 1 hour. After washing away unbound secondary antibodies, the coverslips were mounted using VECTASHIELD^®^ HardSet^TM^ Antifade Mounting Medium with DAPI (Vector Laboratories, H-1500-10). The fluorescence was observed using Nikon confocal microscope (Japan).

**1.5 Participants and Immunohistochemistry**

Normal mammary gland tissue samples (n=12) were collected from patients with breast hyperplasia. TNBC (n=12) and ER-positive (n=12) tissues were obtained from patients who underwent breast mass resection at Harbin Medical University Cancer Hospital (Harbin, China). The patients with TNBC and ER-positive BC have never received the chemotherapy, Endocrine therapy and Small molecule therapy. A series of 5 μm sections were prepared from formalin-fixed, paraffin-embedded specimens. For immunohistochemical analysis, the sections underwent deparaffinized in xylene and rehydrated in graded ethanol series (80–100%). Antigen retrieval was achieved by microwaving the sections in citrate buffer (pH 6.0) for 30 minutes, and non-specific peroxidase reactions were blocked using hydrogen peroxide. Following a brief rinse with PBS, the sections were incubated overnight 4°C with primary antibodies against HIF-1α (1:200, Abcam, ab51608), PKM2(1:200, Abcam, ab150377), Ki67 (1:200, Proteintech, 27309-1-AP) and ENO1 (1:200, Proteintech, 11204-1-AP). The following day, the sections were washed three times with PBS and then incubated at 37°C with biotin-conjugated goat anti-rabbit polyclonal antibody (1:1000, Abcam, ab6720) for 30 minutes. Visualization was achieved by incubating the sections with Alexa Fluor Fluorescent Dyes or diaminobenzidine (ZSGB-BIO, ZLI-9019) with hematoxylin. To detect apoptosis in the sections, the One Step TUNEL Apoptosis Assay Kit (Beyotime, C1088) was utilized following manufacture’s instruction.

**1.6 Cell Counting Kit-8 (CCK8) assay**

Cell proliferation was assessed using CCK-8 kit from Beyotime (C0038) following the manufacture’s instruction. Briefly, TNBC cells were seeded at a density of 10^-5^ cells per well in a 96-well plate and cultured in 100 µl of growth medium with either DMSO or drugs. Wells will no cells but the same volume of medium were used as blank controls. At the end of the treatment, 10 µl of CCK-8 reagent was added to each well, followed by incubating the plate at 37°C for 1 hour. The cell number, indicated by the resulting color change, was quantified in a cell plate reader at 450 nm.

**1.7 Transwell assay**

The cell invasion assay utilized Transwell 24-well plates with 5µm pore size, provided by Corning, and a Matrigel basement membrane acquired from BD. To prepare the Matrigel coating, it was mixed at a 1:8 dilution with serum-free medium and applied to the upper chamber of the Transwell plate. After incubating the Matrigel at 37°C for 30 minutes, the Transwell plate was transferred to a 6-well plate containing 1mL of medium with fetal bovine serum (FBS) in the lower chamber. A total of 5×104 cells suspended in serum-free medium were introduced into the upper chamber. Following a specified incubation duration, the cells that successfully invaded through the Matrigel and migrated to the lower chamber were fixed using 95% ethanol and subsequently stained with hematoxylin and eosin. The invaded cells were then quantified through microscopic examination.

**1.8 Colony-formation assay**

The colony-formation assay was executed to evaluate the cells' capacity to generate colonies. Cells in their logarithmic growth phase were enzymatically dissociated and suspended in DMEM medium. Subsequently, these cells were distributed into 3 cm dishes at a density of 500 cells per dish, with or without prior treatment with sorafenib. The dishes were then maintained under incubation for 2-3 weeks at 37°C in an environment containing 5% CO_2_ and humidity. After this incubation period, the colonies that formed were both counted and photographed. The colony-formation rate was determined by dividing the number of colonies by the initial number of cells seeded and multiplying the result by 100%.

**1.9 Lactate detection assay**

The intracellular L-lactate acid contents were determined using Lactate Detection Kit from Biovision (#K607-100) following the manufacturer’s instruction. Briefly, cells were homogenized in a volumes of lactate assay buffer that was four times their weight. The resulting mixture was then centrifuged at 13,000xg for 10 minutes to eliminate any insoluble materials. Afterwards, the samples underwent deproteinization using a spin filter with a molecular weight cutoff of 10 kDa to remove lactate dehydrogenase. Subsequently, a colorimetric detection was performed using a plate reader set at a wavelength of 570 nm. For absolute quantification, lactate standards for the colorimetric detection were prepared by diluting 10 µl of the 100 nmole/µl lactate standard with 990 µl of lactate assay buffer to generate a 1 nmole/µl lactate standard. These standards were then distributed into a 96-well plate, resulting in standards containing 0, 2, 4, 6, 8, and 10 nmole/well. Lactate assay buffer was added to each well to reach a final volume to 50 µl.

**1.10 Pyruvate Detection Assay**

The intracellular pyruvate levels were assessed using the Pyruvate Detection Kit from Biovision (#K609-100) following the manufacturer’s instructions. Briefly, cells were detached from the culture plate with trypsin and collected by centrifugation. After discarding of the culture medium, the cells were washed three time with ice-cod PBS. The washed cells were then resuspended in ice-cold PBS and subjected to physical disruption using ultrasound sonication, followed by three freeze/thaw cycles. The resulting cell slurry was subsequently centrifuged at 1500xg for 10 minutes at 4°C. The supernatant obtained was further processed for deproteinization by adding perchloric acid (PCA) to a final concentration of 1 M, followed by incubation on ice for 5 minutes. The mixture was then subjected to centrifugation at 13,000rpm for 2 minutes at 4°C to remove precipitated proteins. To neutralize excess PCA in the resulting supernatant, 34% of the volume of ice-cold 2 M KOH was added, and the mixture was further clarified by centrifugation at 13,000rpm for 15 minutes at 4°C. The resulting samples were mixed with 50 µl reaction mixture containing pyruvate enzyme mix and probe, and the incubation was carried out at room temperature for 30 minutes while protecting the samples from light. Subsequently, a colorimetric detection was performed using a plate reader set at a wavelength of 570 nm.

**1.11 Phosphoenolpyruvate (PEP) Detection Assay**

Intracellular PEP levels were determined using the PEP Colorimetric Kit from Biovision (#K365-100) in accordance with the manufacturer’s instructions. Briefly, cells were rapidly frozen in liquid nitrogen and then thoroughly pulverized using a pre-chilled pestle at -80°C. The powdered samples were transferred to a 1.5-ml Eppendorf tube, and 100 µl of ice cold perchloric acid was added, followed by vigorous vortexing to ensure thorough mixing. The mixture was carefully neutralized by adding small aliquots (~10 µl per aliquot) of KHCO_3_ while vortexing. The neutralized mixture was then centrifuge at 12,000g for 3 minutes. Subsequently, up to 50 µl sample was added per well in a 96-well plate, and the volume was adjusted to 50 µl with Assay Buffer. The resulting sample was combined with a 50 µl reaction mixture containing PEP probe, converter, and developer, followed by incubation at room temperature for 1 hour, with protection from light. Finally, a colorimetric detection was conducted using a plate reader set at a wavelength of 570 nm.

**1.12 Enolase Activity Colorimetric Assay**

The ENO1 enzymatic activity was determined using Enolase Activity Colorimetric Assay Kit provided by BioVision (#K691-100). Following the manufacturer’s instructions, one million cells were homogenized with 100 µl ice-cold Enolase Assay Buffer while maintaining the samples on ice. The lysates were subsequently centrifuged at 10,000xg for 5 min at 4°C. Subsequently, 50 µl of the resulting supernatant was added to each well, add the volume was adjusted to a total of 50 µl using Assay Buffer. To create a standard curve for H_2_O_2_, a 10 mM H_2_O_2_ standard was prepared by adding 4 µl of 0.88 M H_2_O_2_ standard to 348 µl of double-distilled H_2_O (ddH_2_O). This 10 mM H_2_O_2_ standard was further diluted to 1 mM by combining 100 µl of the 10 mM H_2_O_2_ with 900 µl of ddH_2_O. Subsequently, 0, 2, 4, 6, 8, and 10 µl of the diluted 1 mM H_2_O_2_ standard were added to a series of wells in a 96-well plate to generate H_2_O_2_ standards at concentrations of 0, 2, 4, 6, 8 and 10 nmol per well. The volume in each well was adjusted to 50 µl with ddH_2_O. The samples and standards in each well were mixed with a 50 µl reaction mixture that contained Enolase Substrate Mix, Converter, developer, and OxiRed^TM^ Probe. The measurement was conducted using a plate reader set at a wavelength of 570 nm in kinetic mode for 20-60 minutes at 25°C.

## **1.13 RNA immunoprecipitation (RIP)**

The RIP was performed using the RIP kit (Thermo Fisher Scientific, MA, USA). The cell supernatants were treated at room temperature for 30 minutes with protein A/G magnetic beads conjugated with normal rabbit IgG (Catalog # PP64B) or rabbit anti human-ENO1 and lamp2 antibodies. Positive control was input, whereas normal rabbit IgG served as the negative control. The RNA-binding Protein-RNA (RIP) complexes were incubated at 4°C overnight. Quantification of the RNA ligands was then performed using qRT-PCR.

**1.14 Exosome extraction**

Cell culture medium was sequential centrifugation at 2000xg and 10,000xg, respectively at 4°C for 20 min. The supernatant was diluted with 5 ml ice-cold PBS and passed through a 0.2 µm filter. The pass-through was further centrifuged at 120,000xg at 4°C for 2 h. Carefully aspirate supernatant with the bottom 1 ml liquid containing precipitate undisturbed and repeat the dilution with 5 ml ice-cold PBS and centrifuge once. The precipitates containing exosomes were resuspended in 200 µl ice-cold PBS.

## **1.15 Fluorescence in situ hybridization (FISH)**

The Cy3-labeled RNA that specifically targeting to the ligand2 was designed and synthesized by RiboBio company (Guangzhou, China). The FISH experiments were implemented using a FISH kit (RiboBio, Guangzhou, China) according to the manufacturer's instructions.

**1.16 Cell proliferation on scaffolds**

ADSCs were seeded onto sterilized Polyglycolic acid(PGA) scaffolds kept in 24-well plates with a cell density of 4×10^4^ per sample. All cells-scaffolds were cultured at 37°C in a 95% humidity and 5% CO_2_ atmosphere. Cell proliferation and death were evaluated by double-color fluorescence staining with a LIVE/DEAD Viability/Cytotoxicity assay (BestBio, Shanghai, China) at day 1, 3 and 5. Based on the manufacturer’s instruction, the scaffolds were submerged in LIVE/DEAD staining solution at 37°C for 2 h, and then photographed with a cell imaging reader (Cytation5, BioTek, USA) to distinguish living cells (green) and dead cells (red).

**1.17 Terminal uridine nick-end labeling (TUNEL) assay**

Tissue sections were stained by TUNEL (BestBio, Shanghai, China) according to the manufacture instruction. Fluorescence was observed under a fluorescence microscope (Eclipse 80i; Nikon Instruments, Melville, New, USA).

**1.18 Statistical Analysis**

The data are depicted as the mean ± SEM. Statistical analysis was conducted using either an unpaired Student’s *t*-test for comparisons involving two treatment groups, or a One-way ANOVA for comparisons involving more than two groups. Homogeneity of variance was verified prior to ANOVA testing; group variances were comparable across all experimental conditions, ensuring validity of the applied statistical comparisons. Prism GraphPad software was employed for these analyses. A *p*-value below 0.05 was regarded as indicative of statistical significance.

**2.Supplementary Figures**

**
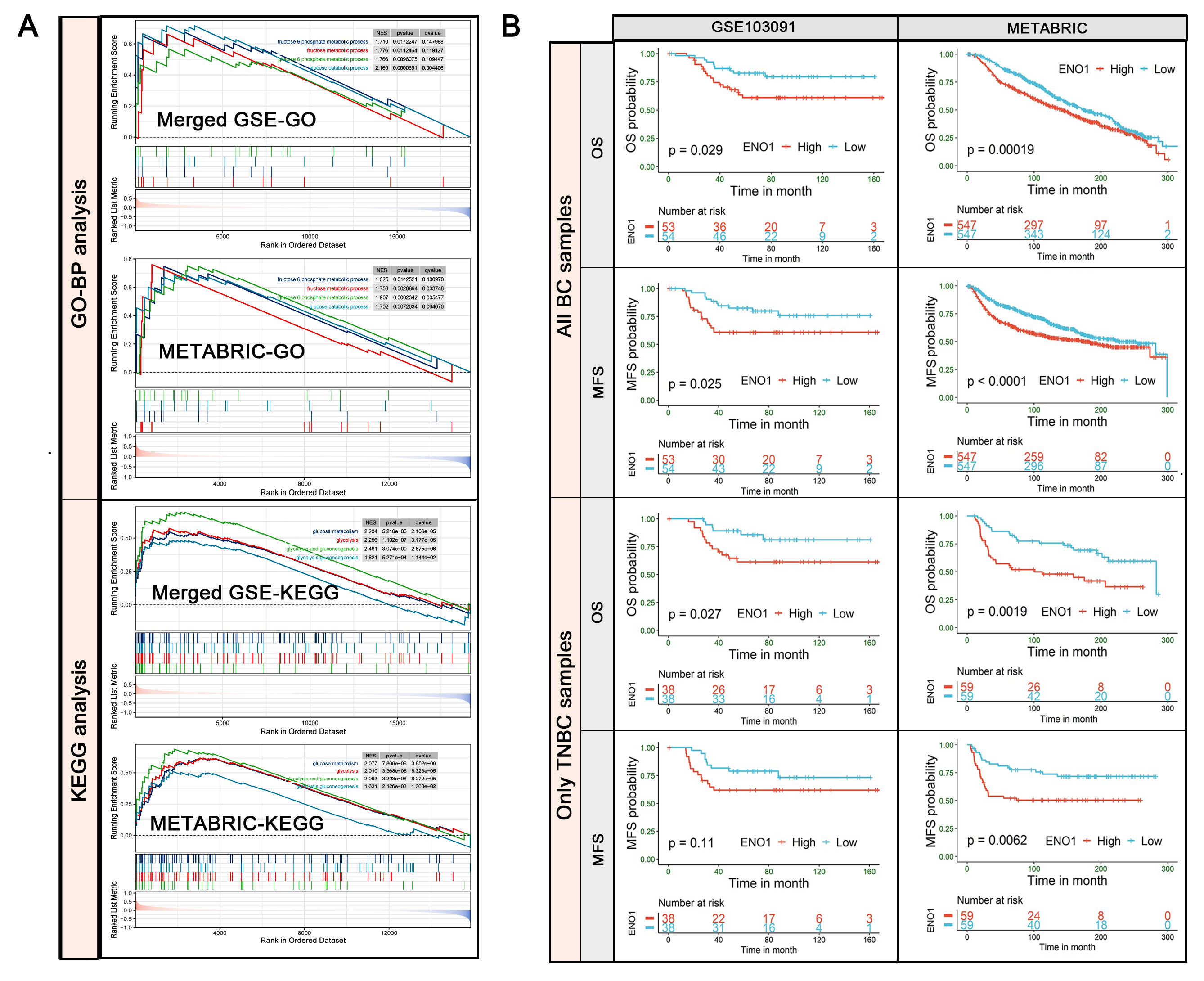
Supplementary Figure 1. Bioinformatic analysis of ENO1-high TNBC subgroups and their association with patient survival.**

**(A)** Gene Ontology (GO) biological process and Kyoto Encyclopedia of Genes and Genomes (KEGG) pathway enrichment analysis of genes upregulated in ENO1-high TNBC tumors from the integrated datasets.

**(B)** Kaplan-Meier survival curves comparing overall survival (OS) and metastasis-free survival (MFS) between breast cancer patients with high and low ENO1 mRNA expression levels across multiple datasets (GSE103091 and METABRIC).

**
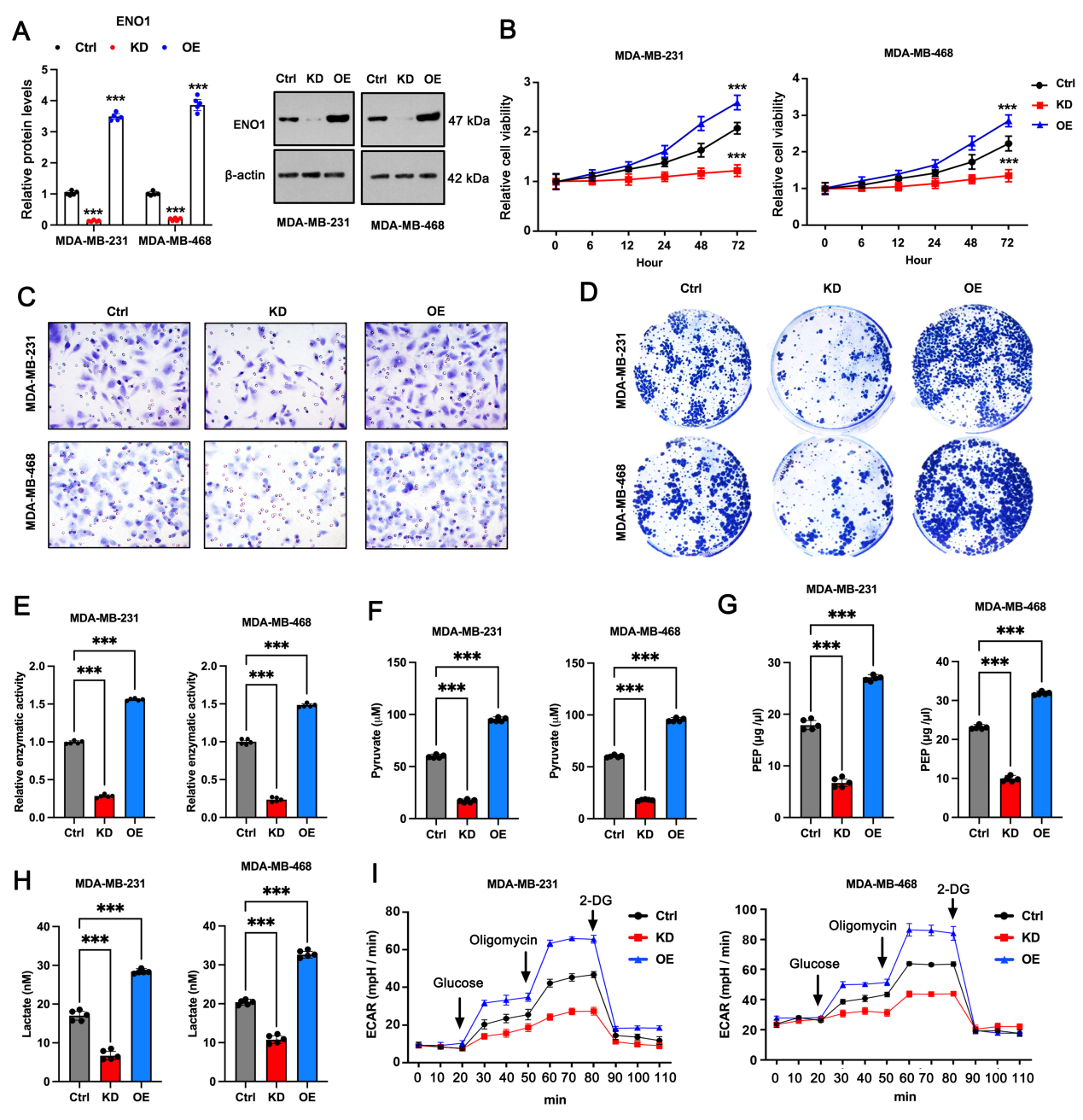
Supplementary Figure 2. Validation of ENO1 knockdown and overexpression and their impact on glycolysis.**

**(A)** Western blot analysis confirming ENO1 protein levels in control (Ctrl), ENO1-knockdown (KD), and ENO1-overexpressing (OE) MDA-MB-231 and MDA-MB-468 cells.

**(B-D)**​Functional assays showing the effect of ENO1 manipulation on cell viability (B), invasion (C), and colony formation (D).

**(E)**​Measurement of ENO1 enzymatic activity in Ctrl, KD, and OE cells.

**(F-H)**​Quantification of intracellular pyruvate (F), phosphoenolpyruvate (PEP, G), and lactate (H) levels.

**(I)**​Extracellular acidification rate (ECAR) measurements and derived glycolytic parameters (glycolytic proton efflux rate, GlycoPER; and glycolytic capacity).

Data represent mean ± SEM (n=5 for all assays); ****p* < 0.001 (a One-way ANOVA for comparisons involving more than two groups).

**
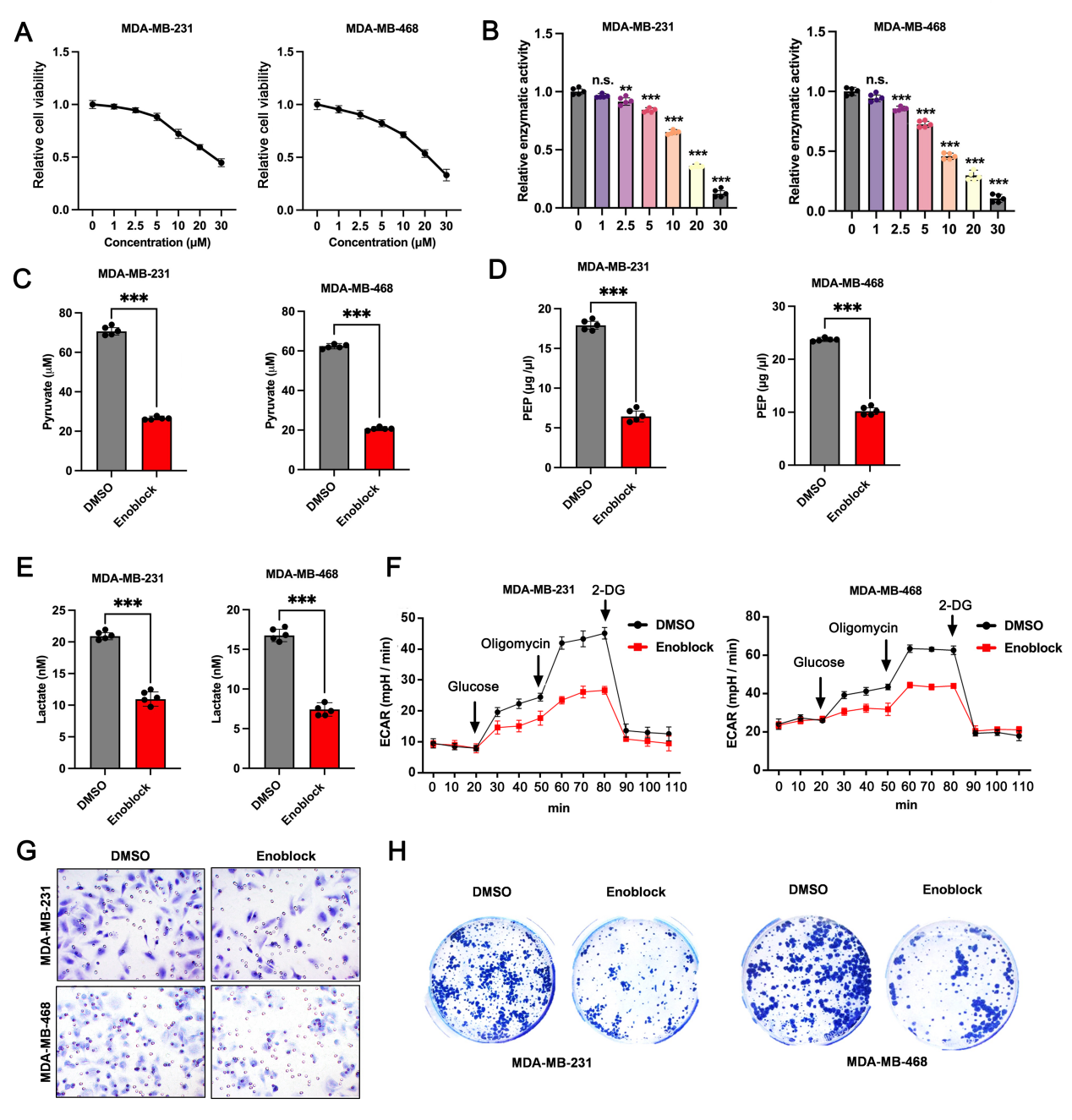
Supplementary Figure 3. Pharmacological inhibition of ENO1 with ENOblock suppresses glycolysis and malignant behaviors.**

**(A, B)**​Cell growth curves of MDA-MB-231 and MDA-MB-468 cells treated with DMSO (vehicle) or various concentrations of ENOblock (A). ENO1 activity in MDA-MB-231 and MDA-MB-468 cells treated with DMSO (vehicle) or various concentrations of ENOblock (B).

**(C-E)​**Levels of pyruvate (C), PEP (D), and lactate (E) in cells after ENOblock treatment.

**(F)​**ECAR measurements showing glycolytic flux after ENOblock treatment.

**(G, H)​**ENOblock treatment inhibits cell invasion (G) and colony formation (H).

Data represent mean ± SEM (n=5 for all assays); ***p* < 0.01, ****p* < 0.001 (unpaired Student’s t-test for comparisons involving two treatment groups, or a One-way ANOVA for comparisons involving more than two groups).

**
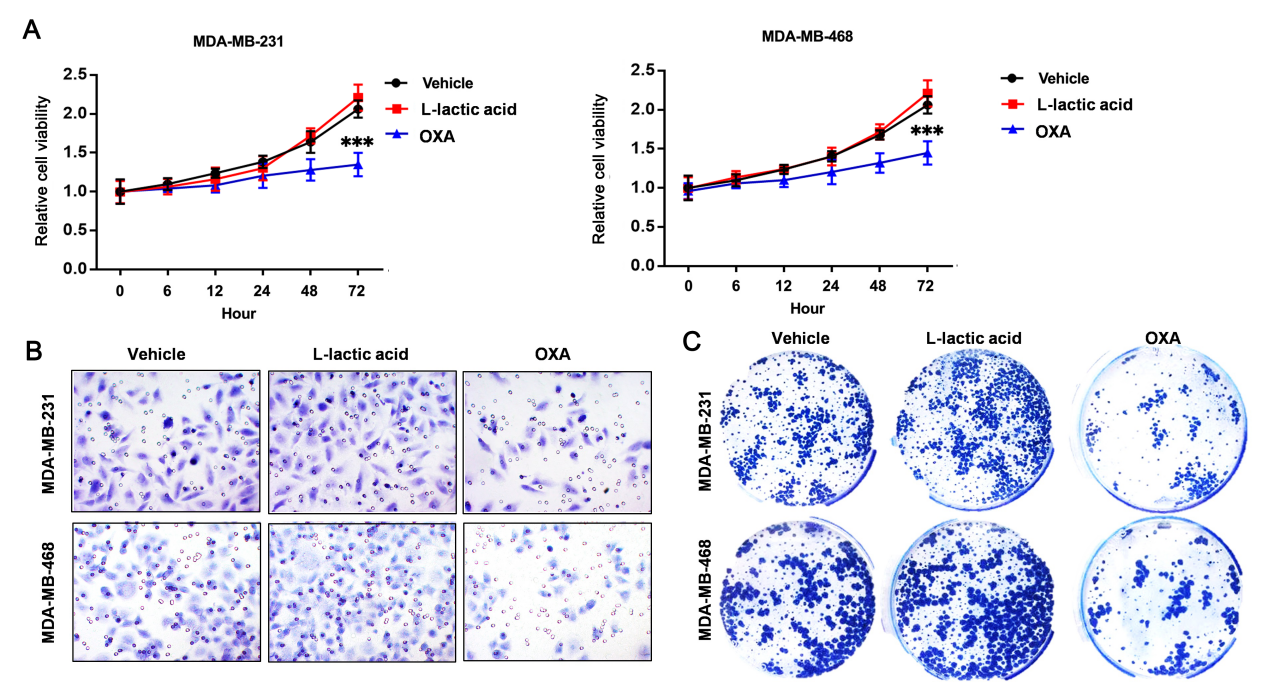
Supplementary Figure 4. Effects of lactate and oxamate on TNBC cell behaviors.**

**(A-C)** Impact of sodium L-lactate and sodium oxamate (OXA) treatment on cell viability (A), invasion (B), and colony formation (C).

Data represent mean ± SEM (n=5 for all assays); ****p* < 0.001 (a One-way ANOVA for comparisons involving more than two groups).

**
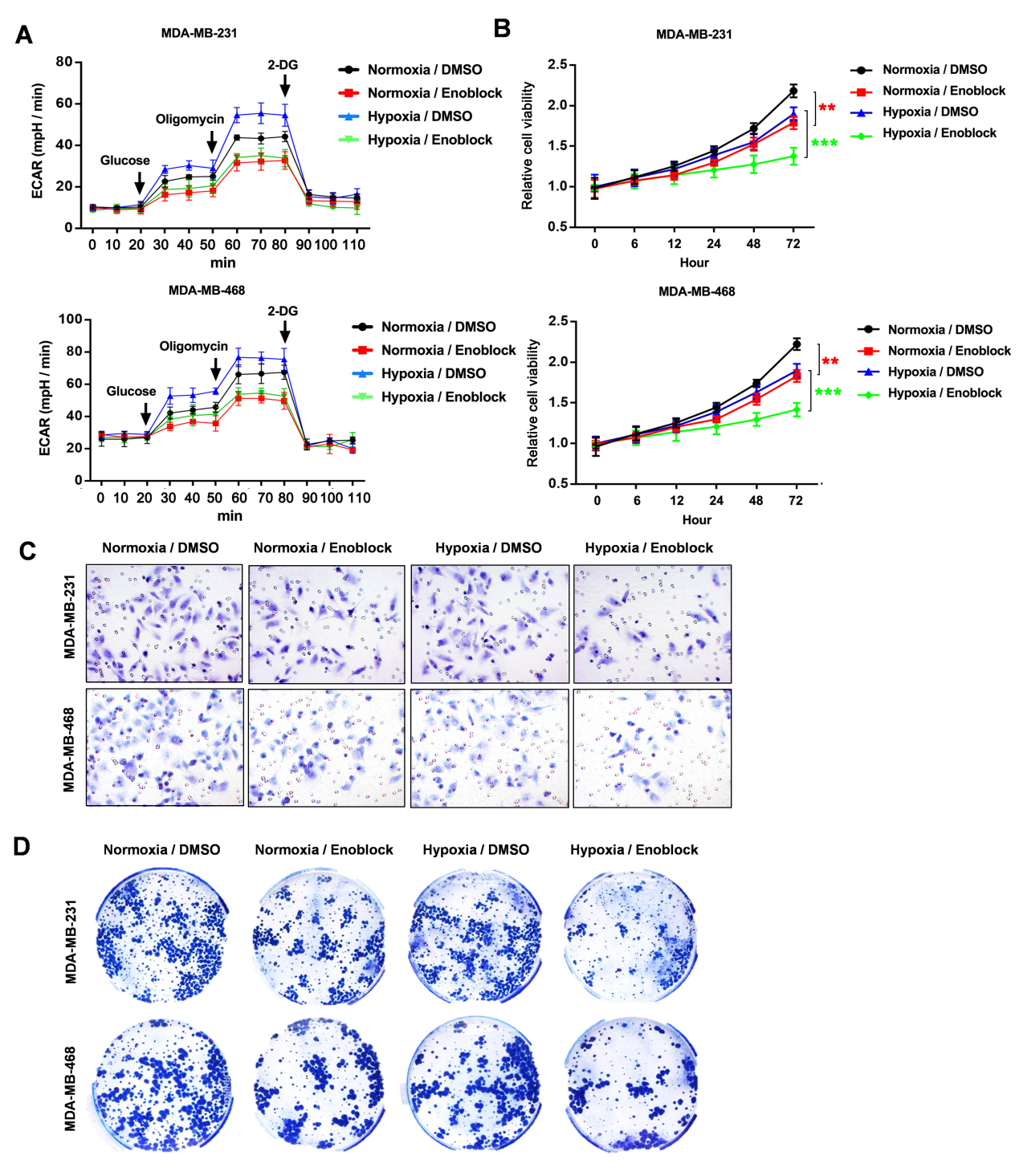
Supplementary Figure 5. The inhibitory effect of ENOblock is enhanced under hypoxic conditions.**

**(A)** ECAR measurements showing the inhibition of glycolysis by ENOblock under normoxia and hypoxia.

**(B-D)** ENOblock treatment under normoxia and hypoxia inhibits cell viability (B), invasion (C), and colony formation (D) in TNBC cells.

Data represent mean ± SEM (n=5 for all assays); **p* < 0.05, ***p* < 0.01, ****p* < 0.001 (a One-way ANOVA for comparisons involving more than two groups).

**
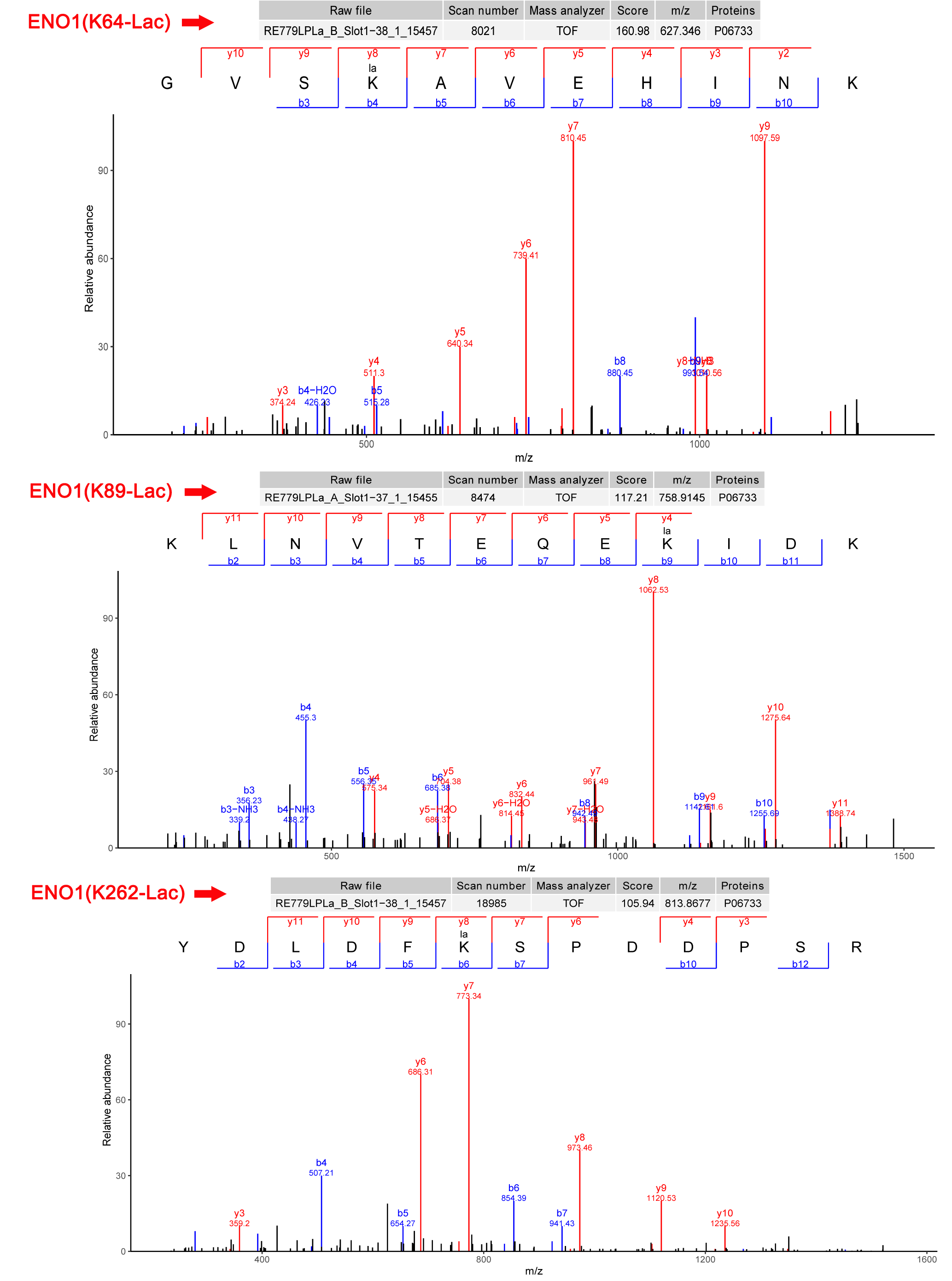
Supplementary Figure 6. Proteomic analysis of ENO1 lactylation sites regulated by EP300.**

Summary data from 4D label-free lactylation proteomics showing specific lactylation sites on ENO1 (K64, K89, K262) that are decreased upon EP300 knockdown in MDA-MB-231 cells.

**
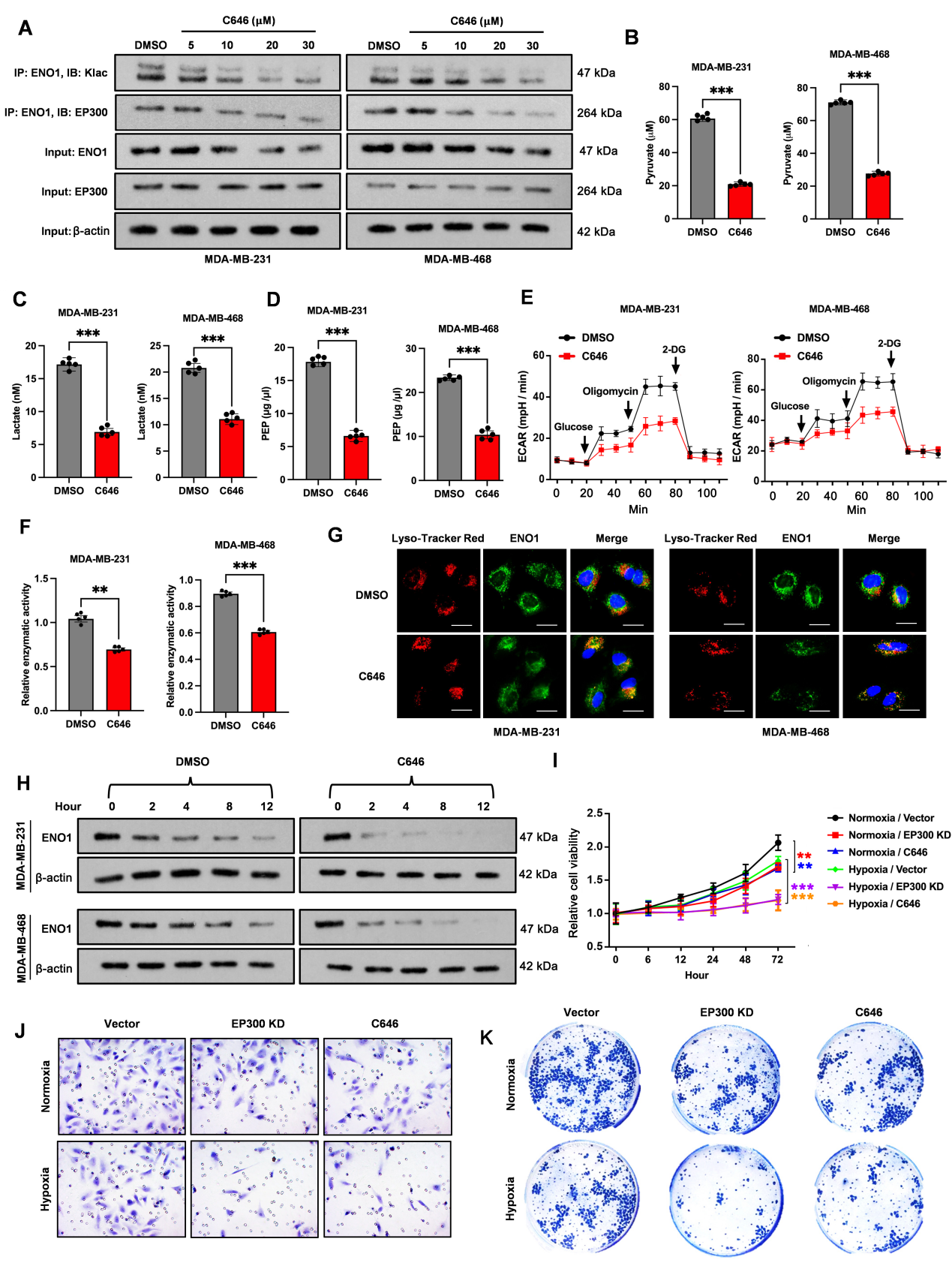
Supplementary Figure 7. Inhibition of EP300 by C646 suppresses ENO1 lactylation, glycolysis, and malignancy.**

**(A)**​Immunoprecipitation and immunoblotting analysis of ENO1 lactylation levels in cells treated with increasing doses of the EP300 inhibitor C646.

**(B-E)**​C646 treatment reduces pyruvate (B), lactate (C), and PEP (D) levels, as well as ECAR parameters (E).

**(F)**​C646 treatment suppresses ENO1 enzymatic activity.

**(G)**​Immunofluorescence showing increased co-localization of ENO1 (green) with lysosomes (red) upon C646 treatment. Scale bar: 5 µm.

**(H)**​Protein stability assay (CHX chase) demonstrating accelerated ENO1 degradation after C646 treatment.

**(I-K)**​EP300 knockdown or C646 inhibition suppresses cell viability (I), invasion (J), and colony formation (K) under normoxic and hypoxic conditions.

Data represent mean ± SEM (n=5 for all assays). ***p* < 0.01, ****p* < 0.001 (unpaired Student’s t-test for comparisons involving two treatment groups, or a One-way ANOVA for comparisons involving more than two groups).

**
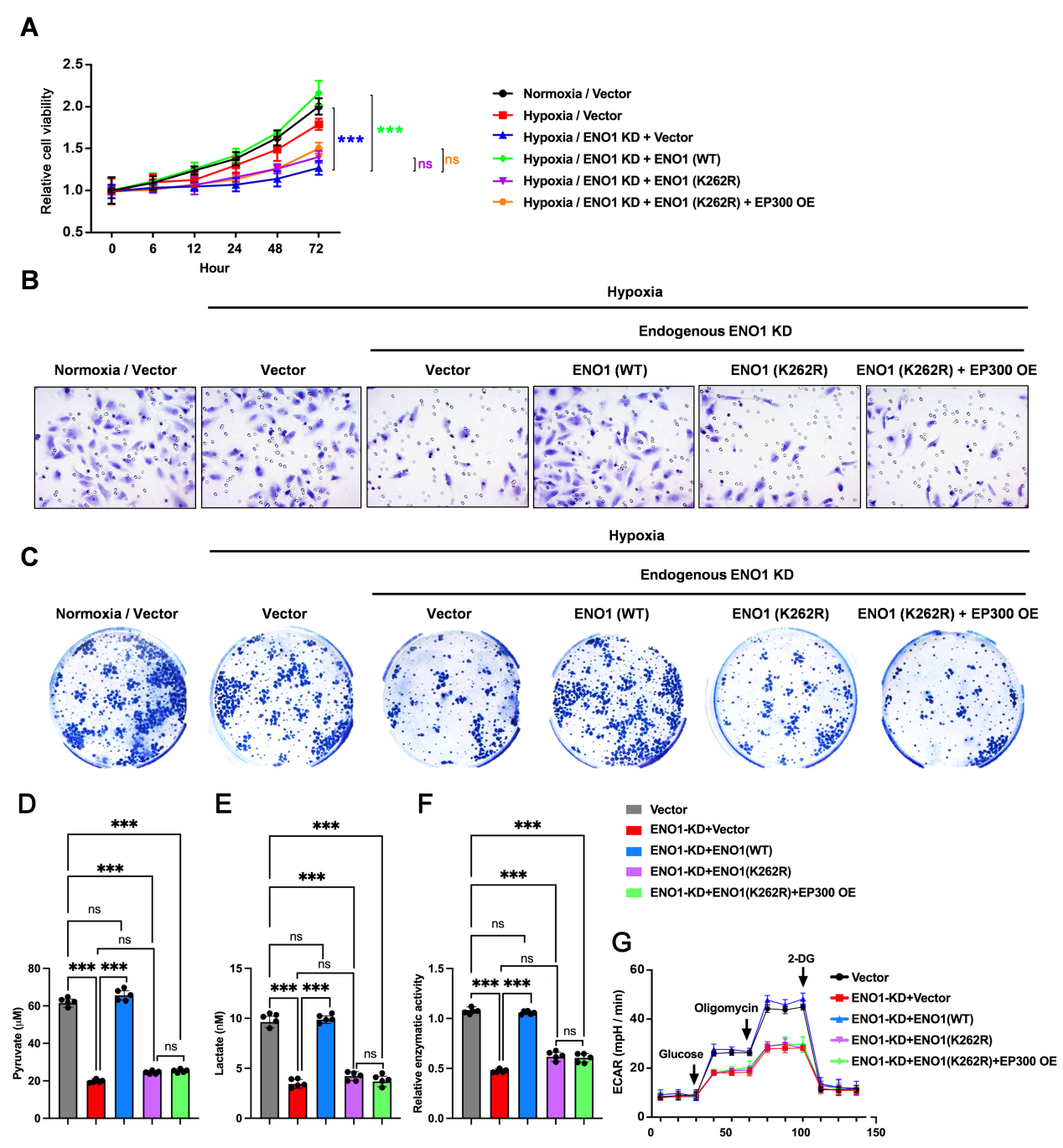
Supplementary Figure 8. The K262R mutant fails to rescue the phenotypic and metabolic defects caused by ENO1 knockdown.**

**(A-C)​**Rescue experiments: Effects on cell viability (A), invasion (B), and colony formation (C) in ENO1-knockdown MDA-MB-231 cells reconstituted with wild-type (WT) or K262R mutant ENO1, with or without EP300 co-expression.

**(D-G)​** Rescue experiments: Effects on pyruvate levels (D), lactate levels (E), ENO1 enzymatic activity(F), and ECAR parameters (G) in the same cell models.

Data represent mean ± SEM (n=5 for all assays). ****p* < 0.001 (a One-way ANOVA for comparisons involving more than two groups).

**
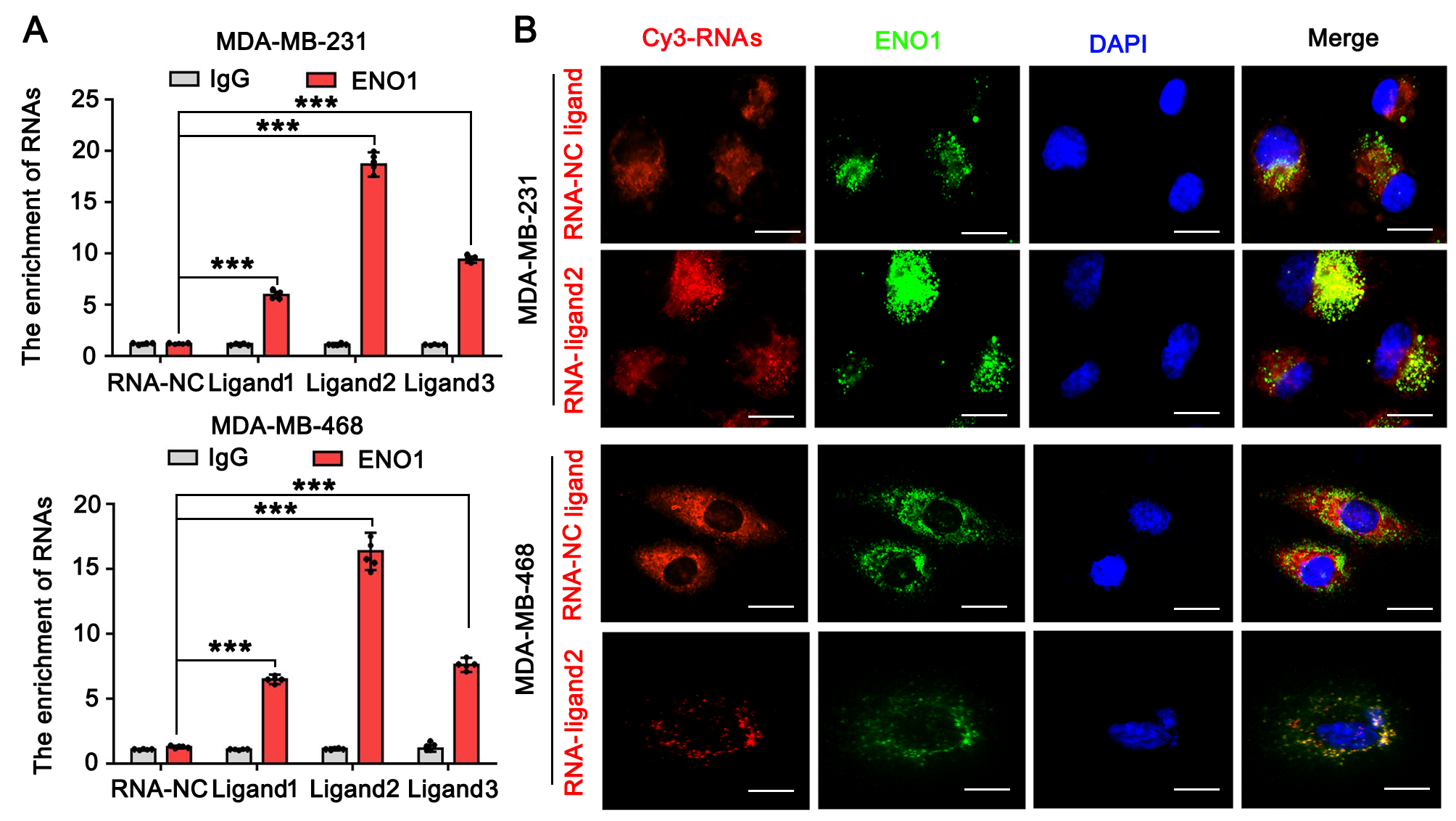
Supplementary Figure 9. Screening and validation of RNA ligands targeting ENO1.**

**(A)​**RNA immunoprecipitation (RIP) assay using an anti-ENO1 antibody to evaluate the binding affinity of three candidate RNA ligands (Ligand 1, 2, 3) to ENO1.

**(B)**​Immunofluorescence showing co-localization of the selected Ligand 2 (red) with the ENO1 protein (green) in TNBC cells. Scale bar: 5 µm.

Data represent mean ± SEM (n=5 for all assays). ****p* < 0.001 (a One-way ANOVA for comparisons involving more than two groups).

**
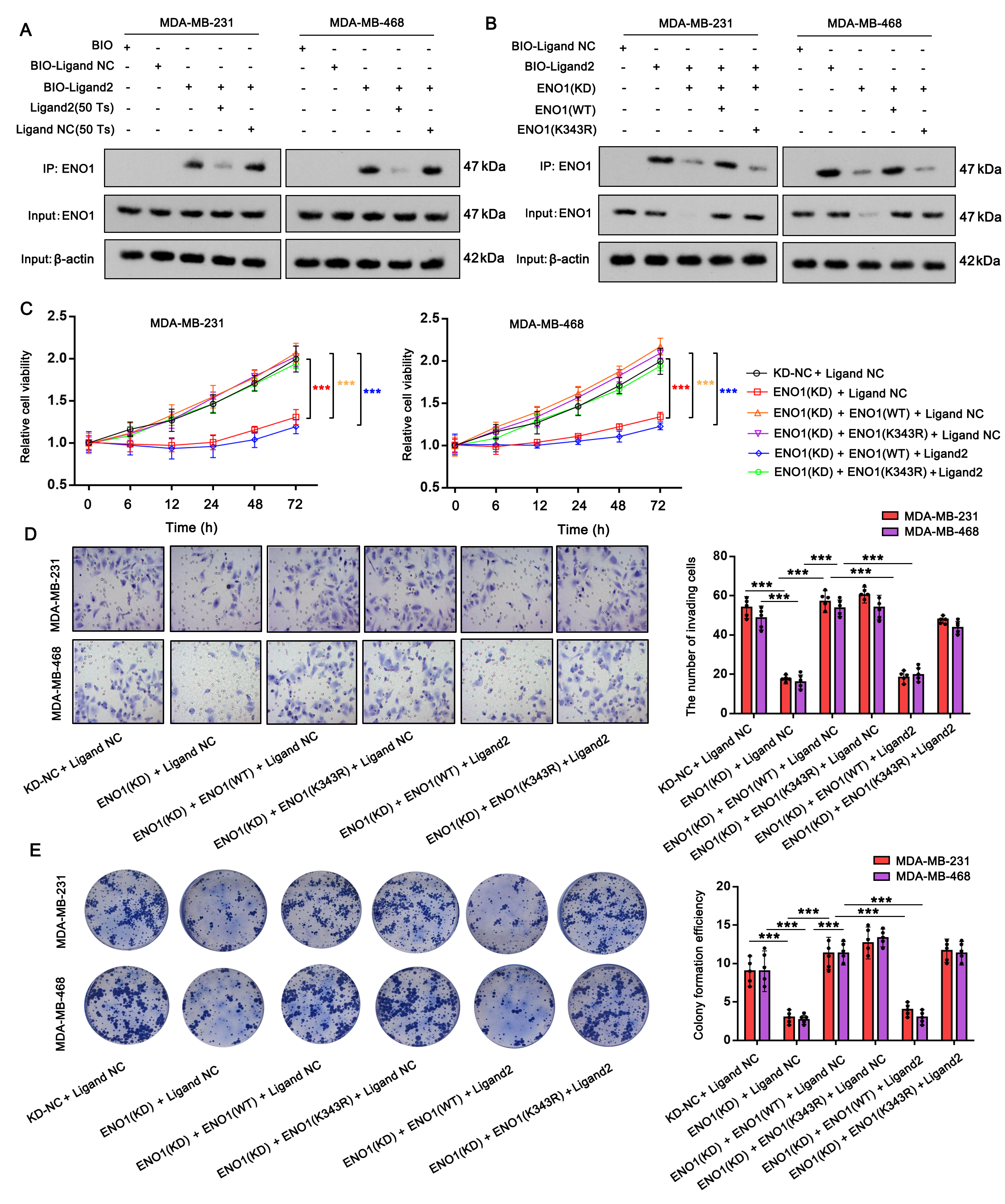
Supplementary Figure 10. Specificity of Ligand 2 binding to ENO1's RNA-binding domain.**

**(A)**​Competitive biotinylated RNA pull-down assay. The ligand 2 sequence and a scrambled control RNA sequence were synthesized and labeled with biotin at the 3' end. MDA-MB-231 and MDA-MB-468 cell lysates were incubated with the biotinylated RNAs for 2 hours at 4°C to allow for RNA-protein binding. To test specificity, a 50-fold excess of unlabeled (non-biotinylated) ligand 2 or unlabeled scrambled RNA was added to the reaction mixture as a specific or non-specific competitor, respectively, prior to the addition of the biotinylated RNA. Biotinylated Ligand 2, but not a scrambled control RNA, pulls down ENO1. The interaction is competed by an excess of unlabeled Ligand 2 but not by unlabeled scrambled RNA. 50 Ts：50 times; NC control: A scrambled RNA scaffold lacking the specific binding regions.

**(B)**​Biotinylated RNA pull-down assay in ENO1-knockdown cells reconstituted with wild-type ENO1 (WT) or the RNA-binding-deficient K343R mutant. Ligand 2 efficiently pulls down ENO1-WT but not the ENO1-K343R mutant.

**(C-E)** On-target validation rescue assay. ENO1-knockdown cells were reconstituted with ENO1-WT (binds ligand) or ENO1-K343R (cannot bind ligand). Lig2-Exo specifically inhibits malignancy only in cells reconstituted with ENO1-WT, but not in cells with ENO1-K343R, demonstrating on-target action. Assays show viability (C), invasion (D), and colony formation (E).

Data represent mean ± SEM (n=5 for all assays). ****p* < 0.001 (a One-way ANOVA for comparisons involving more than two groups).

**
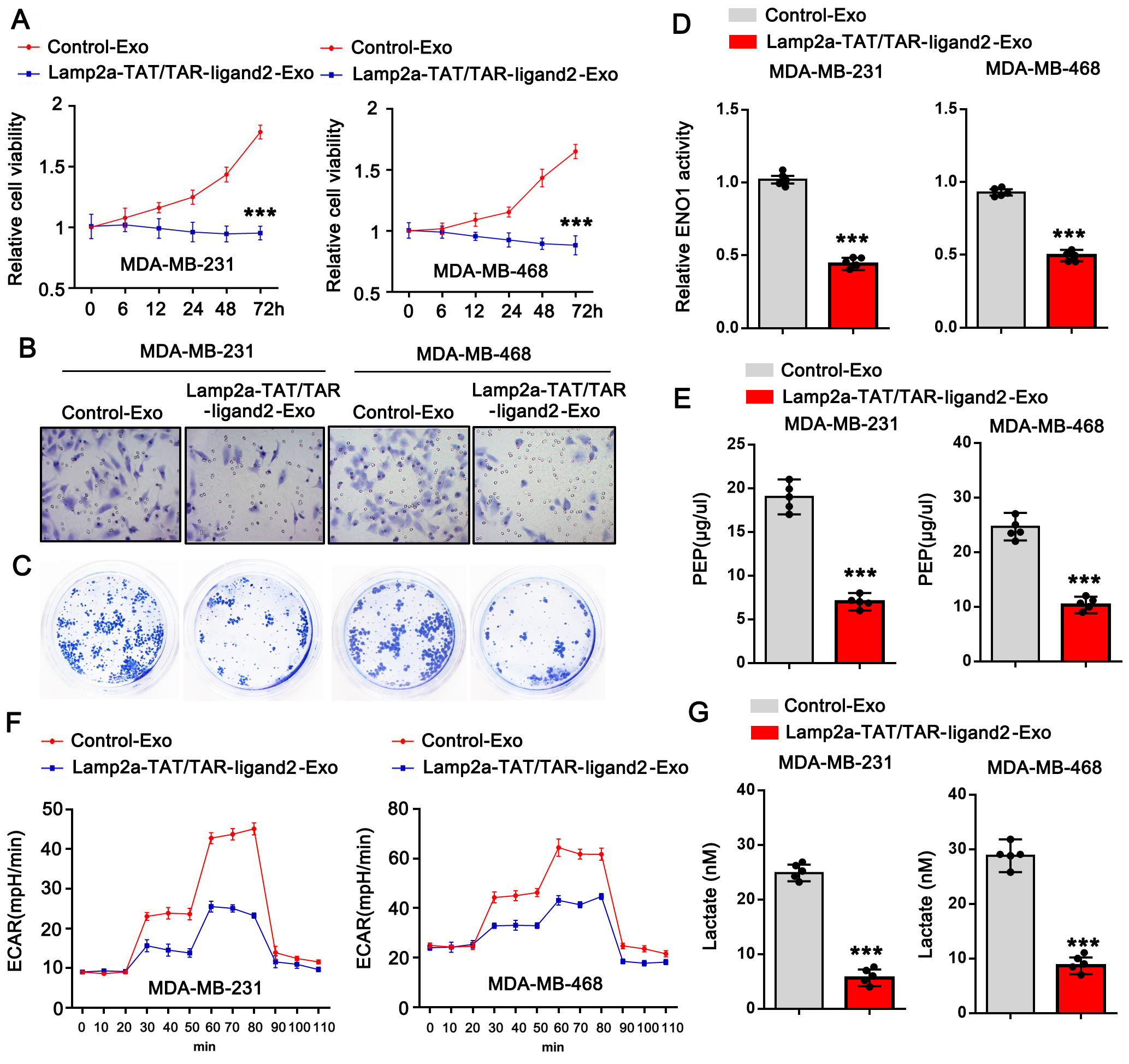
Supplementary Figure 11 Engineered exosomes containing Lamp2a-TAT/TRA-ligand 2 suppress TNBC cell malignancy via on-target degradation of ENO1.**

**(A-C)**​Treatment with exosomes containing Lamp2a-TAT/TRA-ligand 2 (Lig2-Exo) suppresses the viability (A), invasion (B), and colony formation (C) of TNBC cells under hypoxia.

**(D-G)**​Lig2-Exo treatment reduces ENO1 enzymatic activity (D), PEP levels (E), ECAR parameters (F), and lactate levels (G) under hypoxia.

Data represent mean ± SEM (n=5 for all assays). ****p* < 0.001 (unpaired Student’s t-test for comparisons involving two treatment groups).
