# Supplementary material for: A Lamp2a-linked RNA secreted by ADSCs prevents ENO1–lactylation–glycolysis feedback and cell malignant behavior in triple-negative breast cancer
Source: Cell Death Dis. 2026 Mar 2;17(1):288. doi: 10.1038/s41419-026-08517-3 (PMC13031273; doi:10.1038/s41419-026-08517-3)
Supplement: Supplementary file 2 — Supplementary Table 1 [file 41419_2026_8517_MOESM2_ESM.docx]

**Supplementary Table 1. The RNA ligands that is specifically bound by ENO1**

|  | Sequence(5'-3') |
| --- | --- |
| NC | CCACCCCCTCAATCTGTCGGGGAGCCCCTGCCCTTCACCTAGCTCCCTTGGCCAGGAGCTGTGGCCTTGGTGAAGCTGCCCTCCTCTTCTCCCCTCACACTACAGCCCTGGTGGGGGAGAAGGGGGTGGGTGCTGCTTGTGGTTTAGTCAACAGGCTTCAATCTGGAATCAGAAAGCGGTGGATTCTGGCAAATGGTCCTTGTGCCCTCCCCACTCATCCCTGGTCTGGTCCCCTGTTGCCTATAGCCCTTTACCCTGAG |
| Ligand1 | CTTCCTCTTCTTCTTCTTCCTCCTCATCTTCCTCCTCCTCGTCGTCTTCCTCCCCTTCCCCTGCTAAGCCTGGCCCTCAGGCCTTGCCCAAACCTGCAAGCCCCAGACAGCCACCCCCTGGCGAGCGGAGGTGAGTGCTGTCTTGCCTGAGTTGAAAGGTGGGTGGGGGAGTGACTTGTCCAGAGAAGGGGCCCTGGGGTGTGAGCTCCCCGCTGGGTGTCTCACGTGGCCTTGGGCATCTGGTTGTGGGGGAGGA |
| Ligand2 | CCACCCCCTCAATCTGTCGGGGAGCCCCTGCCCTTCACCTAGCTCCCTTGGCCAGGAGCTGTGGCCTTGGTGAAGCTGCCCTCCTCTTCTCCCCTCACACTACAGCCCTGGTGGGGGAGAAGGGGGTGGGTGCTGCTTGTGGTTTAGTCTTTTTTTTTTTTTTTTTTTTAACAGGCTTCAATCTGGAATCAGAAAGCGGTGGATTCTGGCAAATGGTCCTTGTGCCCTCCCCACTCATCCCTGGTCTGGTCCCCTGTTGCCTATAGCCCTTTACCCTGAG |
| Ligand3 | GACGGCCTGGCCGAGGACATCGATAAAGGCGAGGTGTCCGCCCGTCAGGAGCTCAAGCAGCGGGCGCGCTACCTGGCCGAGAAGTACGAGTGGGACGTGGCTGAGGCCCGCAAGATCTGGTGCTTTGGGCCCGACGGCACCGGCCCCAACATCCTCACCGACATCACCA |
