# Supplementary material for: A Lamp2a-linked RNA secreted by ADSCs prevents ENO1–lactylation–glycolysis feedback and cell malignant behavior in triple-negative breast cancer
Source: Cell Death Dis. 2026 Mar 2;17(1):288. doi: 10.1038/s41419-026-08517-3 (PMC13031273; doi:10.1038/s41419-026-08517-3)
Supplement: Supplementary file 3 — Supplementary Table 2 [file 41419_2026_8517_MOESM3_ESM.docx]

**Supplementary Table 2. The primers for the PCR assay.**

|  | Direction | Primer (5'-3') |
| --- | --- | --- |
| ENO1 | F | AAAGCTGGTGCCGTTGAGAA |
|  | R | GGTTGTGGTAAACCTCTGCTC |
| β-actin | F | CATGTACGTTGCTATCCAGGC |
|  | R | CTCCTTAATGTCACGCACGAT |
| Ligand1 | F | TCATCTTCCTCCTCCTCGTC |
|  | R | CCCCTTCTCTGGACAAGTCA |
| Ligand2 | F | GAAGCTGCCCTCCTCTTCTC |
|  | R | ACAAGGACCATTTGCCAGAA |
| Ligand3 | F | CATCGATAAAGGCGAGGTGT |
|  | R | GGTGATGTCGGTGAGGATGT |
