# Supplementary material for: A Lamp2a-linked RNA secreted by ADSCs prevents ENO1–lactylation–glycolysis feedback and cell malignant behavior in triple-negative breast cancer
Source: Cell Death Dis. 2026 Mar 2;17(1):288. doi: 10.1038/s41419-026-08517-3 (PMC13031273; doi:10.1038/s41419-026-08517-3)
Supplement: Supplementary file 4 — Supplementary Table 3 [file 41419_2026_8517_MOESM4_ESM.docx]

**Supplementary Table3. The detail information of primary antibodies for Western blot assay.**

| Name | Company | Catalog number | Dilution |
| --- | --- | --- | --- |
| Lactyllysine | PTM Bio | PTM-1401 | 1:200 |
| ENO1 | Ptgcn | 67187-1-Ig | 1:200 |
| EP300 | Boster | M00117-1 | 1:500 |
| lamp2 | Abcam | AB199946 | 1:500 |
| HA | Ptgcn | 51064-2-AP | 1:500 |
| FLAG | Ptgcn | 66008-4-Ig | 1:500 |
| HK1 | Ptgcn | 68419-1-Ig | 1:200 |
| GPI | Boster | M00108-2 | 1:200 |
| PFKP | Ptgcn | 68129-1-Ig | 1:200 |
| PGK1 | Ptgcn | 68035-1-Ig | 1:200 |
| PKM2 | Boster | M01173-1 | 1:200 |
| HIF-1α | Ptgcn | 66730-1-Ig | 1:200 |
| β-ACTIN | Ptgcn | 66009-1-Ig | 1:500 |
