# Supplementary material for: A Lamp2a-linked RNA secreted by ADSCs prevents ENO1–lactylation–glycolysis feedback and cell malignant behavior in triple-negative breast cancer
Source: Cell Death Dis. 2026 Mar 2;17(1):288. doi: 10.1038/s41419-026-08517-3 (PMC13031273; doi:10.1038/s41419-026-08517-3)
Supplement: Supplementary file 5 — Supplementary Table 4 [file 41419_2026_8517_MOESM5_ESM.docx]

**Supplementary Table 4. Proteomics demonstrated significantly differentially expressed proteins (DEPs) between TNBC and ER+ BC**

| **Symbol** | **nonTNBC-1** | **nonTNBC-2** | **nonTNBC-3** | **TNBC-1** | **TNBC-2** | **TNBC-3** |
| --- | --- | --- | --- | --- | --- | --- |
| COX8C | 12.3591 | 12.7336 | 11.8519 | 8.9375 | 9.3387 | 8.3798 |
| HIF1A | 6.6445 | 6.7394 | 6.5428 | 9.2103 | 9.1781 | 9.2418 |
| STOML2 | 9.3583 | 9.8625 | 8.5765 | 6.2208 | 6.3024 | 6.1344 |
| LDHA | 10.6718 | 10.3133 | 10.9587 | 12.7105 | 12.8629 | 12.5401 |
| ENO1 | 6.1115 | 6.1766 | 6.0434 | 9.7379 | 9.4624 | 9.9692 |
| RHOA | 12.4399 | 12.6713 | 12.1642 | 9.1567 | 9.4777 | 8.7432 |
| GPI | 6.6502 | 7.1327 | 5.92 | 9.4919 | 9.4537 | 9.5291 |
| NDUFA12 | 12.1453 | 11.6929 | 12.4892 | 7.7697 | 7.9153 | 7.6078 |
| PGK | 9.3239 | 8.8089 | 9.7027 | 12.0381 | 11.4783 | 12.4404 |
| ALDH5A1 | 12.4599 | 12.9201 | 11.7802 | 8.8092 | 8.8206 | 8.7977 |
| GHITM | 11.9705 | 12.4051 | 11.3455 | 9.2444 | 8.7772 | 9.5968 |
| ATP5PB | 12.3298 | 12.7037 | 11.8237 | 9.0511 | 8.9947 | 9.1053 |
| RECK | 10.3021 | 10.458 | 10.1274 | 12.5182 | 13.0724 | 11.6066 |
| DIXDC1 | 8.2658 | 7.8604 | 8.582 | 12.368 | 12.7016 | 11.9332 |
| MSH2 | 12.4686 | 12.6049 | 12.3181 | 9.298 | 8.9272 | 9.5928 |
| ILK | 10.0511 | 10.3861 | 9.614 | 12.7421 | 13.278 | 11.88 |
| INSR | 6.4221 | 6.7173 | 6.0506 | 9.3592 | 9.3465 | 9.3718 |
| MDH2 | 11.4394 | 11.267 | 11.5935 | 7.1563 | 7.3408 | 6.9446 |
| NDUFB2 | 11.6303 | 11.826 | 11.4038 | 6.7676 | 6.409 | 7.0546 |
| TNKS2 | 7.3027 | 7.1631 | 7.43 | 11.558 | 11.8455 | 11.1986 |
| SCEL | 9.8368 | 9.7639 | 9.9062 | 11.8917 | 12.4074 | 11.0814 |
| ATP5PO | 12.1057 | 12.0642 | 12.146 | 9.75 | 9.3906 | 10.0375 |
| HK1 | 10.0337 | 10.4061 | 9.5305 | 12.3235 | 12.2742 | 12.3712 |
| MDH1B | 11.841 | 12.3136 | 11.1335 | 5.628 | 6.1252 | 4.8632 |
| SMURF2 | 9.8897 | 10.3902 | 9.1169 | 11.9748 | 12.3687 | 11.4312 |
| PKM2 | 8.2513 | 7.855 | 8.5619 | 12.4318 | 12.5873 | 12.2575 |
| OGT | 6.6359 | 7.1131 | 5.9179 | 8.789 | 8.3612 | 9.1186 |
| PFKP | 6.6135 | 6.7587 | 6.4521 | 9.5764 | 9.6678 | 9.4789 |
| TAFAZZIN | 11.3863 | 11.8699 | 10.6535 | 6.4285 | 6.8458 | 5.8391 |
| COA6 | 11.2991 | 11.6041 | 10.9119 | 7.0198 | 7.1428 | 6.8853 |
| SLC25A33 | 12.7637 | 13.0937 | 12.335 | 8.8522 | 8.7152 | 8.9772 |
| CDK1 | 11.6227 | 11.4938 | 11.7411 | 7.0126 | 6.7079 | 7.2641 |
| ZNRF3 | 8.8933 | 9.1912 | 8.5174 | 12.3779 | 12.2843 | 12.4659 |
| CHCHD10 | 11.7192 | 11.1657 | 12.1183 | 9.6205 | 9.5059 | 9.7266 |
| CAPRIN2 | 9.9924 | 10.3045 | 9.5935 | 12.076 | 12.3537 | 11.7316 |
| NIT2 | 12.7947 | 12.6207 | 12.9501 | 9.3562 | 9.8638 | 8.5663 |
| PCK2 | 12.0275 | 12.6017 | 11.0592 | 9.1112 | 9.6164 | 8.3271 |
| TTC37 | 7.4689 | 7.3229 | 6.4866 | 3.2167 | 3.8376 | 2.9909 |
| SRPX | 13.3765 | 14.8186 | 15.4326 | 7.1873 | 6.0289 | 7.5134 |
| RSPH1 | 15.2224 | 13.7874 | 13.559 | 3.2619 | 3.3887 | 3.0693 |
| CASP8AP2 | 4.7908 | 4.4685 | 4.7884 | 9.1126 | 9.7165 | 10.2571 |
| GOLM1 | 11.9653 | 14.2146 | 11.9194 | 5.8257 | 6.3998 | 5.3793 |
| RIMS3 | 5.12 | 4.167 | 4.8008 | 15.5483 | 13.6717 | 13.9183 |
| PIM1 | 6.7782 | 6.0272 | 7.5789 | 13.644 | 14.9553 | 15.5256 |
| IFT74 | 12.1152 | 10.4835 | 12.663 | 9.7098 | 10.307 | 10.3739 |
| C3orf80 | 11.5147 | 11.7644 | 12.4978 | 7.1055 | 7.3121 | 5.7057 |
| ERICH5 | 4.7607 | 3.8669 | 4.0579 | 10.1532 | 10.6603 | 11.8274 |
| CNTNAP2 | 9.8829 | 11.1148 | 10.68 | 6.4034 | 6.629 | 5.8994 |
| PRC1 | 13.0693 | 14.6523 | 14.0076 | 15.6182 | 18.3483 | 18.2162 |
| SLC22A5 | 14.3533 | 15.1844 | 12.4935 | 9.107 | 7.4142 | 10.0933 |
| CTSC | 12.0329 | 14.0897 | 13.2892 | 15.1914 | 15.2539 | 16.9628 |
| GPAM | 15.4089 | 15.0591 | 17.0888 | 4.2527 | 4.1842 | 4.5551 |
| LAMP5 | 15.5854 | 16.7047 | 13.7989 | 8.513 | 8.2894 | 8.8621 |
| NEK11 | 10.5439 | 11.2144 | 12.3163 | 4.2695 | 4.6132 | 4.0788 |
| CMYA5 | 9.155 | 10.2999 | 10.4567 | 6.7791 | 6.2892 | 6.5706 |
| GPRC5A | 6.7561 | 6.9016 | 7.0811 | 4.4285 | 4.5469 | 3.7422 |
| WWP1 | 12.4274 | 10.623 | 11.0009 | 4.1741 | 4.2297 | 3.6689 |
| VSNL1 | 5.7453 | 6.5188 | 5.8048 | 9.4371 | 8.6974 | 9.4306 |
| MAP4K4 | 9.6224 | 10.3583 | 9.0789 | 15.9444 | 17.5531 | 14.2836 |
| ADORA2B | 8.5409 | 9.4018 | 7.889 | 12.0965 | 12.5728 | 10.1885 |
| GAL | 8.5299 | 7.563 | 9.8052 | 11.6091 | 13.5259 | 11.9461 |
| MLF2 | 5.4214 | 5.3703 | 4.7528 | 6.9399 | 7.4268 | 6.2188 |
| CCDC68 | 15.5719 | 14.7526 | 12.5224 | 10.3608 | 9.1375 | 11.2386 |
| CHN2 | 12.8161 | 14.5951 | 14.0714 | 9.6126 | 8.2114 | 8.2818 |
| MTX3 | 13.3661 | 12.3769 | 13.1554 | 11.6266 | 10.8431 | 11.8683 |
| PLOD1 | 9.7611 | 9.614 | 8.8861 | 14.0936 | 15.1347 | 11.8948 |
| CAB39L | 9.3957 | 11.0627 | 9.0763 | 5.6099 | 5.5545 | 4.7136 |
| EPHB3 | 7.0914 | 6.6102 | 6.1493 | 12.1766 | 12.7979 | 13.1473 |
| FITM2 | 10.9248 | 9.2972 | 12.2453 | 6.0169 | 5.7689 | 7.0567 |
| FGB | 11.6373 | 9.4706 | 13.6293 | 8.2937 | 8.1858 | 7.8256 |
| LAYN | 8.8125 | 10.4147 | 7.2153 | 6.6672 | 5.8753 | 6.7344 |
| CRIP2 | 9.104 | 7.4419 | 8.2785 | 5.3672 | 4.4078 | 6.2915 |
| CTPS1 | 10.4594 | 11.9841 | 9.6513 | 12.8864 | 13.6863 | 11.8595 |
| C3orf52 | 13.4071 | 14.4425 | 12.1079 | 5.0756 | 5.9278 | 5.8584 |
| ETV6 | 5.7826 | 5.2365 | 5.6845 | 12.6182 | 10.8317 | 14.0753 |
| S100A6 | 7.1107 | 5.7829 | 6.0454 | 15.8733 | 16.0356 | 13.2347 |
| KNL1 | 6.635 | 6.6635 | 7.4987 | 10.5132 | 12.4279 | 12.1004 |
| MCM6 | 6.9239 | 5.613 | 8.0433 | 10.2596 | 8.9968 | 10.2205 |
| CITED1 | 8.3702 | 8.8486 | 9.4806 | 6.3072 | 6.1664 | 6.7139 |
| CYBRD1 | 14.3535 | 17.0066 | 13.3829 | 9.9386 | 8.5066 | 9.7828 |
| SHROOM2 | 11.9194 | 12.2121 | 10.322 | 3.912 | 4.4424 | 4.5677 |
| RBMS3 | 7.6277 | 8.9497 | 7.4241 | 5.0577 | 5.5122 | 4.9817 |
| CNTN4 | 11.7749 | 9.8839 | 12.284 | 5.6114 | 5.5735 | 6.3928 |
| FANCI | 7.4452 | 8.4887 | 6.1973 | 15.0743 | 15.1605 | 13.5321 |
| RHBDF2 | 6.9077 | 6.764 | 5.9225 | 14.5729 | 12.6457 | 16.2152 |
| DOK7 | 14.9839 | 17.342 | 14.408 | 4.4697 | 5.0745 | 4.9908 |
| RUNX3 | 5.066 | 4.7145 | 4.543 | 14.4772 | 15.6083 | 17.0519 |
| PKP1 | 13.0016 | 11.4898 | 13.2974 | 15.5596 | 13.6348 | 15.71 |
| TSHZ1 | 10.3086 | 9.1443 | 11.7598 | 4.8856 | 5.7244 | 4.2118 |
| AGO2 | 6.5173 | 7.6447 | 6.8567 | 9.3292 | 9.1868 | 9.1003 |
| MAP3K1 | 15.0834 | 17.374 | 13.8056 | 4.7126 | 3.8624 | 5.1376 |
| ZNF292 | 12.5224 | 11.43 | 10.8673 | 13.8504 | 15.1019 | 15.4319 |
| HID1 | 11.9598 | 13.5682 | 10.5319 | 5.7963 | 5.1275 | 5.8325 |
| SLC7A1 | 8.9219 | 9.0628 | 9.5286 | 11.7833 | 10.2073 | 14.0518 |
| ID4 | 5.425 | 4.429 | 4.6659 | 15.0312 | 16.1753 | 15.6571 |
| ART3 | 7.6079 | 6.4642 | 6.5287 | 11.6748 | 11.3518 | 13.9398 |
| KCNK5 | 7.6992 | 6.6454 | 6.5666 | 10.0928 | 10.0061 | 8.9224 |
| CTSK | 12.971 | 12.0387 | 15.3071 | 8.6704 | 10.3211 | 9.9031 |
| TNNI2 | 3.631 | 3.7936 | 4.0769 | 8.6916 | 8.875 | 9.915 |
| BAG6 | 6.1329 | 5.3729 | 5.3119 | 14.5391 | 16.7613 | 11.6774 |
| MFAP4 | 11.6939 | 12.6184 | 13.3982 | 7.5221 | 6.4617 | 7.5936 |
| RASD2 | 4.7648 | 3.8607 | 4.1092 | 12.946 | 14.8529 | 14.0382 |
| LGR6 | 3.5392 | 3.9386 | 3.7465 | 14.1914 | 16.7999 | 14.8014 |
| SERPINB7 | 5.1461 | 5.1882 | 6.0777 | 8.3585 | 8.0359 | 9.4765 |
| BBS4 | 10.0343 | 10.2792 | 8.9671 | 7.5003 | 6.2544 | 8.2802 |
| NDUFAF4 | 3.6572 | 4.2294 | 4.3621 | 6.1039 | 6.8208 | 7.2649 |
| SCD | 12.7726 | 14.0473 | 10.284 | 5.5725 | 5.5116 | 4.9975 |
| GPRIN2 | 6.4442 | 6.1061 | 7.064 | 12.5881 | 13.087 | 12.503 |
| ZNF367 | 8.8176 | 10.2263 | 8.1367 | 12.9981 | 15.0457 | 14.9183 |
| TRMT10A | 9.6892 | 10.1667 | 11.3992 | 7.9997 | 7.2695 | 9.3834 |
| USP1 | 7.278 | 7.7419 | 7.4981 | 12.7645 | 14.7686 | 13.6185 |
| MAGEA4 | 11.7072 | 10.0828 | 10.2381 | 13.9324 | 13.9141 | 13.9534 |
| TRAF3IP1 | 10.3951 | 9.9874 | 12.1559 | 6.8948 | 5.6216 | 5.7341 |
| ELF4 | 5.3926 | 4.8659 | 6.1001 | 6.8809 | 7.9984 | 6.3993 |
| TEX10 | 9.5769 | 7.8807 | 9.6182 | 15.8677 | 14.2279 | 15.7567 |
| ASAH1 | 13.5368 | 15.032 | 13.0879 | 5.3606 | 4.7482 | 4.6991 |
| YBX1 | 9.1116 | 8.9203 | 9.0173 | 14.8529 | 11.8931 | 13.0895 |
| MSLN | 3.7059 | 3.5478 | 3.7054 | 14.5087 | 13.6101 | 16.897 |
| CFAP53 | 14.412 | 16.3685 | 14.4125 | 4.3236 | 4.2165 | 4.6256 |
| ZMAT1 | 11.2408 | 9.3561 | 10.6104 | 4.8121 | 4.5777 | 5.0662 |
| SFT2D2 | 5.0022 | 5.4843 | 4.9301 | 13.3056 | 14.3354 | 11.146 |
| MOXD1 | 6.2068 | 5.0997 | 6.1564 | 10.5439 | 10.4438 | 11.3836 |
| PAPSS1 | 4.1344 | 4.8485 | 4.7819 | 7.6137 | 7.1876 | 7.2517 |
| ASNS | 3.5712 | 2.8672 | 3.0927 | 12.0713 | 9.7391 | 13.9643 |
| KIFC1 | 5.0438 | 4.3038 | 4.9575 | 13.4715 | 11.0003 | 14.1853 |
| PFDN2 | 7.0482 | 7.0471 | 8.0331 | 13.5127 | 13.4243 | 13.0302 |
| NUBPL | 14.6958 | 12.9192 | 16.4537 | 6.3541 | 7.479 | 6.9331 |
| AUH | 12.0317 | 12.9948 | 12.187 | 9.6151 | 7.8254 | 11.1593 |
| SCNN1A | 10.5316 | 11.57 | 9.3303 | 8.1862 | 7.3053 | 6.7843 |
| CDH3 | 3.9115 | 4.2334 | 3.4201 | 12.3117 | 11.5999 | 10.1608 |
| UEVLD | 8.2253 | 8.8503 | 8.6758 | 6.0164 | 4.903 | 4.8875 |
| CENPA | 5.647 | 5.7529 | 5.7322 | 12.1411 | 10.7574 | 10.0434 |
| MAK | 15.0524 | 16.9165 | 14.1486 | 3.2176 | 3.4468 | 2.7025 |
| RAP2A | 5.8057 | 6.7734 | 6.7365 | 15.9771 | 15.9005 | 13.7589 |
| ENPP1 | 12.954 | 15.1592 | 12.5326 | 5.5764 | 4.9368 | 4.9179 |
| TET3 | 5.3945 | 5.3497 | 4.8146 | 15.7654 | 17.2 | 18.8467 |
| RRAGD | 11.0314 | 10.1764 | 10.7612 | 13.9145 | 14.3223 | 15.1518 |
| RAVER2 | 10.1076 | 8.6351 | 10.0437 | 13.5036 | 13.0989 | 14.3597 |
| POLR3C | 9.9606 | 10.1719 | 9.0569 | 12.4286 | 11.7887 | 14.6225 |
| OGN | 14.8797 | 12.9516 | 13.6807 | 4.1275 | 3.5256 | 4.807 |
| BACE2 | 8.7334 | 7.6284 | 10.4398 | 10.1834 | 11.8119 | 10.8586 |
| RBM47 | 10.7565 | 8.9503 | 10.5838 | 6.46 | 7.6772 | 7.0553 |
| LRP12 | 3.9355 | 3.19 | 4.0076 | 6.815 | 6.17 | 6.9657 |
| RPP25 | 4.2229 | 3.4867 | 4.7874 | 15.4204 | 13.1828 | 15.9605 |
| OCA2 | 7.356 | 7.4879 | 6.6739 | 13.3726 | 11.0341 | 14.4483 |
| SLC18B1 | 5.0939 | 5.6191 | 5.6561 | 14.7768 | 14.9336 | 13.0453 |
| NSMCE1 | 11.9902 | 11.0657 | 10.99 | 3.2786 | 2.6926 | 3.0941 |
| ZNF385B | 12.436 | 13.1965 | 10.2436 | 8.6782 | 10.05 | 8.8008 |
| UTP4 | 4.9962 | 5.8919 | 5.9323 | 7.677 | 8.4836 | 7.5173 |
| LY6K | 11.4949 | 12.0838 | 9.7979 | 13.8112 | 12.0351 | 15.3413 |
| PODXL | 10.1216 | 8.504 | 11.6833 | 14.2702 | 16.5337 | 12.0161 |
| RPIA | 8.4446 | 7.9959 | 8.4817 | 15.7809 | 16.7724 | 17.1599 |
| CDCA3 | 3.6843 | 4.1911 | 3.1433 | 12.7236 | 12.7722 | 11.2339 |
| ATP11C | 8.3742 | 8.9968 | 8.2689 | 10.6524 | 12.4229 | 11.9827 |
| COL22A1 | 9.5307 | 7.9422 | 8.1057 | 10.5159 | 12.4835 | 11.3027 |
| KERA | 14.8398 | 14.127 | 15.3458 | 7.5131 | 6.1599 | 6.4231 |
| ITGB5 | 7.7711 | 8.1485 | 9.0961 | 3.9925 | 3.867 | 4.6607 |
| CNKSR3 | 6.3351 | 6.2239 | 5.3831 | 7.9524 | 8.8231 | 6.7177 |
| LMNA | 4.2218 | 4.0204 | 4.1772 | 7.8635 | 7.7098 | 7.3187 |
| SRPX2 | 15.1182 | 17.1053 | 13.6338 | 9.7318 | 9.9132 | 10.8368 |
| ZNF667 | 4.1641 | 4.7219 | 3.7879 | 11.0642 | 11.713 | 12.0159 |
| POU2AF1 | 4.4316 | 5.2201 | 3.8245 | 6.9496 | 7.0925 | 7.7032 |
| TSPAN13 | 8.898 | 9.4975 | 7.3436 | 4.2571 | 4.6515 | 4.7222 |
| CDC42EP5 | 3.5533 | 3.8012 | 2.9588 | 8.7669 | 7.497 | 8.0087 |
| C17orf58 | 14.1392 | 13.2632 | 11.7113 | 11.6984 | 9.5321 | 9.7995 |
| PRKCI | 6.0178 | 6.9769 | 5.4997 | 12.6664 | 13.1334 | 13.7538 |
| CHEK2 | 4.5268 | 5.0893 | 4.912 | 13.4406 | 14.0561 | 11.4458 |
| CYP27C1 | 7.2893 | 8.0195 | 6.0012 | 12.9602 | 12.1144 | 10.8917 |
| POGK | 7.2532 | 7.7509 | 7.3645 | 10.2479 | 9.9441 | 10.1739 |
| STK39 | 11.5643 | 12.9012 | 13.0482 | 6.9197 | 6.1689 | 7.9215 |
| NUSAP1 | 10.204 | 9.9642 | 9.8068 | 13.9456 | 14.6718 | 11.9581 |
| MRPL51 | 8.4109 | 7.1647 | 9.5027 | 11.4349 | 9.2728 | 11.1146 |
| CP | 8.3576 | 9.9954 | 6.6866 | 15.9189 | 13.205 | 13.2806 |
| NUF2 | 5.6098 | 6.2168 | 6.5438 | 11.1921 | 10.7905 | 13.4171 |
| DPT | 12.1013 | 14.1556 | 10.9905 | 8.8409 | 10.2199 | 8.401 |
| SLC18A2 | 10.8308 | 10.1147 | 12.7476 | 7.786 | 9.2589 | 8.8404 |
| AK6 | 13.1005 | 11.0825 | 11.1697 | 7.4262 | 8.608 | 7.7885 |
| TTC30A | 7.3981 | 7.868 | 6.1385 | 3.8631 | 4.4794 | 4.1613 |
| REEP5 | 11.2716 | 10.8028 | 12.7584 | 3.4926 | 3.7342 | 4.1273 |
| THEMIS2 | 3.9076 | 3.6924 | 4.5487 | 7.1185 | 7.3702 | 8.2617 |
| HJURP | 6.4359 | 7.2271 | 5.9411 | 8.8737 | 8.2245 | 9.6469 |
| ORAI3 | 7.5375 | 7.6016 | 8.651 | 4.7628 | 4.5565 | 4.323 |
| EPN3 | 10.3653 | 11.0658 | 8.4427 | 6.3678 | 7.0796 | 7.1135 |
| OSR1 | 10.2412 | 9.5768 | 9.6317 | 14.5842 | 12.8345 | 13.9695 |
| C5orf49 | 14.8292 | 13.499 | 13.527 | 10.3255 | 11.3605 | 10.8024 |
| MAP9 | 13.6224 | 12.8964 | 15.8549 | 3.3116 | 3.8725 | 3.5478 |
| MPZL2 | 7.4599 | 8.4765 | 6.2122 | 13.2809 | 14.663 | 10.9415 |
| PELI1 | 7.2291 | 6.2794 | 7.859 | 12.6681 | 12.8752 | 14.9034 |
| TRIM8 | 8.2503 | 9.7389 | 6.9168 | 4.28 | 3.5068 | 4.3898 |
| PDE7A | 9.0509 | 7.7905 | 9.9621 | 14.3347 | 13.3887 | 14.8909 |
| MDM1 | 14.4071 | 13.1922 | 16.378 | 10.2296 | 10.6252 | 10.7717 |
| ANP32E | 7.6502 | 7.426 | 6.2863 | 15.1776 | 12.3412 | 14.2212 |
| BARD1 | 8.6685 | 9.1305 | 7.3895 | 13.1251 | 13.4502 | 15.5959 |
| GATM | 15.0268 | 12.1583 | 16.9422 | 12.1345 | 12.6675 | 9.9145 |
| PDE8B | 11.4277 | 11.3098 | 13.4629 | 9.6459 | 10.019 | 10.6865 |
| MCM10 | 10.1528 | 8.5857 | 9.3328 | 15.3056 | 15.4348 | 14.3955 |
| TK1 | 8.6258 | 8.4392 | 7.6152 | 15.1642 | 16.6408 | 12.6653 |
| HELLS | 4.2227 | 4.1831 | 3.718 | 5.633 | 6.0587 | 6.3442 |
| HEPACAM2 | 15.2963 | 18.2249 | 17.1101 | 10.2455 | 8.6855 | 9.4419 |
| LONRF2 | 12.6244 | 10.1518 | 11.4492 | 7.2519 | 7.2669 | 8.0923 |
| BLM | 7.1215 | 6.2112 | 8.3491 | 13.2724 | 12.9007 | 11.6564 |
| PDZK1 | 10.0313 | 9.7598 | 9.3917 | 6.0395 | 6.3019 | 6.4878 |
| FBL | 5.7607 | 5.2437 | 6.359 | 9.2413 | 9.9266 | 9.3621 |
| CXXC5 | 9.9026 | 9.6725 | 9.825 | 5.3441 | 5.8309 | 4.8068 |
| SYNDIG1 | 15.4511 | 13.3892 | 18.3332 | 9.7102 | 8.9398 | 9.447 |
| UVSSA | 14.2455 | 11.6469 | 15.6367 | 10.3175 | 9.7691 | 12.236 |
| ICA1 | 13.5091 | 14.7248 | 11.5211 | 4.0473 | 3.6269 | 3.5756 |
| ALG13 | 11.977 | 12.8631 | 11.6749 | 9.2816 | 8.4443 | 8.5297 |
| PDXK | 6.846 | 6.6163 | 7.8228 | 13.3761 | 15.9521 | 11.6934 |
| TCF7L1 | 7.5204 | 6.5623 | 6.3847 | 10.5497 | 9.9089 | 11.7399 |
| TIMM50 | 12.3328 | 12.2547 | 13.656 | 15.6506 | 16.3323 | 17.1042 |
| ETNK2 | 15.3542 | 15.8981 | 12.5535 | 10.4074 | 11.1663 | 9.0535 |
| APOBEC3B | 7.1877 | 6.9915 | 7.0561 | 13.8078 | 16.3106 | 12.8737 |
| FOS | 7.4055 | 6.7908 | 6.8366 | 3.0752 | 3.2086 | 3.0008 |
| ITGA2 | 11.3408 | 9.4205 | 12.488 | 3.8809 | 4.3454 | 3.1974 |
| GCFC2 | 13.4067 | 11.1818 | 11.811 | 15.4482 | 17.6731 | 17.4114 |
| TBCK | 14.2456 | 13.8699 | 14.657 | 9.1669 | 8.195 | 9.2495 |
| SPOCK1 | 9.036 | 7.2431 | 8.5159 | 3.2757 | 3.6815 | 3.8576 |
| BORCS7 | 6.1353 | 5.5832 | 7.3422 | 3.4418 | 3.4897 | 3.7617 |
| KDM1B | 3.8328 | 4.1583 | 3.8597 | 8.878 | 10.0006 | 8.2444 |
| DNAJC9 | 4.6512 | 4.5595 | 5.4499 | 15.4218 | 14.4757 | 17.4367 |
| SPIN4 | 6.2437 | 6.0946 | 5.6591 | 14.8066 | 13.1092 | 15.5616 |
| NAA10 | 5.2592 | 6.0931 | 5.7575 | 11.9123 | 13.1402 | 13.2453 |
| CERS6 | 8.6726 | 10.1213 | 7.2843 | 6.2376 | 7.2564 | 6.0176 |
| BMPR1B | 15.3144 | 13.8068 | 16.9477 | 4.9285 | 5.8729 | 4.0877 |
| IQCG | 3.5508 | 3.1036 | 3.609 | 6.6807 | 5.5192 | 5.4617 |
| ROPN1 | 12.5169 | 14.2775 | 10.4269 | 13.7642 | 15.4772 | 14.0529 |
| POPDC3 | 9.2126 | 8.9526 | 8.1585 | 15.5091 | 16.8714 | 14.0063 |
| DIAPH3 | 7.5047 | 7.6381 | 7.7995 | 15.6831 | 17.145 | 16.9507 |
| ZNF703 | 9.5692 | 7.7385 | 9.772 | 3.7335 | 4.1295 | 3.4403 |
| PRCC | 6.3946 | 6.6536 | 6.2147 | 10.6245 | 10.0492 | 10.6764 |
| CENPN | 6.6802 | 7.8036 | 6.296 | 10.2737 | 11.8774 | 9.9361 |
| GRHL1 | 11.436 | 10.2783 | 10.3668 | 13.2653 | 12.2659 | 11.3226 |
| ISG15 | 5.725 | 4.7569 | 6.0139 | 15.4257 | 17.2792 | 16.8614 |
| CDKN2C | 6.5327 | 5.3529 | 6.4057 | 8.1086 | 8.1695 | 7.8852 |
| TAF5 | 5.1137 | 4.9756 | 5.0397 | 13.0112 | 10.6139 | 11.0685 |
| CIAO1 | 7.8677 | 9.0968 | 6.8434 | 12.5532 | 12.6665 | 14.2534 |
| COL14A1 | 7.6406 | 7.7327 | 8.3227 | 6.139 | 5.9195 | 5.4411 |
| EZH2 | 9.6224 | 8.5377 | 7.7981 | 10.8443 | 9.6283 | 10.6644 |
| FBLN1 | 11.441 | 13.5735 | 11.4317 | 7.8887 | 6.4139 | 8.6629 |
| CCDC170 | 12.9433 | 12.9828 | 13.1124 | 3.4816 | 3.3628 | 3.467 |
| CDC123 | 12.0772 | 10.1382 | 10.4736 | 13.0665 | 15.109 | 15.1776 |
| FAM174B | 14.6048 | 12.8617 | 16.6284 | 3.4718 | 3.5175 | 3.491 |
| DNMBP | 10.3335 | 10.1328 | 9.0731 | 7.2014 | 6.1977 | 6.3469 |
| ANGPT1 | 12.7359 | 10.6927 | 11.8563 | 15.2579 | 13.3952 | 15.1432 |
| UPP1 | 7.3194 | 8.6216 | 7.193 | 10.7631 | 12.4074 | 10.1362 |
| PIP | 9.579 | 11.344 | 8.8095 | 5.749 | 5.8554 | 6.1257 |
| FBN3 | 6.9503 | 7.3462 | 7.98 | 14.5305 | 14.4789 | 12.5803 |
| FGFR3 | 12.6142 | 14.4774 | 10.9907 | 7.1381 | 6.3663 | 6.2736 |
| MTFR2 | 8.3583 | 7.0784 | 9.6259 | 11.0252 | 12.2118 | 10.942 |
| SYT17 | 10.4236 | 12.1339 | 12.005 | 9.518 | 9.2388 | 10.9938 |
| SLC16A5 | 9.015 | 10.2703 | 7.7821 | 5.6173 | 5.3552 | 5.683 |
| TBC1D7 | 6.0164 | 6.3718 | 5.1342 | 10.1358 | 9.9163 | 9.4485 |
| SAMD12 | 15.0337 | 12.1125 | 16.9515 | 8.4302 | 10.0318 | 9.0445 |
| BAIAP2L1 | 5.7222 | 5.8694 | 5.4238 | 9.368 | 8.2439 | 11.0802 |
| GPC4 | 7.6934 | 8.7265 | 8.217 | 3.9836 | 3.6312 | 4.4265 |
| CCNA2 | 13.0505 | 12.5237 | 12.6572 | 13.3955 | 15.6803 | 15.1334 |
| ABCC3 | 13.4864 | 11.4465 | 11.0032 | 5.4974 | 6.2099 | 6.4914 |
| KCND2 | 15.5343 | 15.7377 | 15.4956 | 7.518 | 8.7918 | 7.9556 |
| CFB | 14.825 | 16.4475 | 17.0879 | 10.1341 | 11.7365 | 10.1385 |
| NCAPG | 5.3113 | 5.3475 | 4.2817 | 9.2699 | 10.8343 | 9.2325 |
